# A Comparison of Neural Decoding Methods and Population Coding Across Thalamo-Cortical Head Direction Cells

**DOI:** 10.3389/fncir.2019.00075

**Published:** 2019-12-10

**Authors:** Zishen Xu, Wei Wu, Shawn S. Winter, Max L. Mehlman, William N. Butler, Christine M. Simmons, Ryan E. Harvey, Laura E. Berkowitz, Yang Chen, Jeffrey S. Taube, Aaron A. Wilber, Benjamin J. Clark

**Affiliations:** ^1^Department of Statistics, Florida State University, Tallahassee, FL, United States; ^2^Department of Psychological and Brain Sciences, Center for Cognitive Neuroscience, Dartmouth College, Hanover, NH, United States; ^3^Department of Psychology, Program in Neuroscience, Florida State University, Tallahassee, FL, United States; ^4^Department of Psychology, University of New Mexico, Albuquerque, NM, United States

**Keywords:** spatial behavior, navigation, memory, anterior thalamus, parahippocampal, parietal

## Abstract

Head direction (HD) cells, which fire action potentials whenever an animal points its head in a particular direction, are thought to subserve the animal’s sense of spatial orientation. HD cells are found prominently in several thalamo-cortical regions including anterior thalamic nuclei, postsubiculum, medial entorhinal cortex, parasubiculum, and the parietal cortex. While a number of methods in neural decoding have been developed to assess the dynamics of spatial signals within thalamo-cortical regions, studies conducting a quantitative comparison of machine learning and statistical model-based decoding methods on HD cell activity are currently lacking. Here, we compare statistical model-based and machine learning approaches by assessing decoding accuracy and evaluate variables that contribute to population coding across thalamo-cortical HD cells.

## Introduction

Animals can navigate by monitoring an online record of their spatial orientation in an environment and using this information to produce direct trajectories to hidden goals (Cullen and Taube, 2017; Epstein et al., 2017; Moser et al., 2017). Head direction (HD) cells, which fire action potentials whenever an animal points its head in a particular direction, are thought to subserve the animal’s sense of spatial orientation (Taube et al., 1990a, b; Taube, 1995, 2007). The direction of maximum response, or the preferred firing direction, varies between cells, such that a small population of HD cells can encode the full range of possible HDs. HD cells are found prominently in anterior thalamic nuclei (ATN), including the anterodorsal, anteroventral, and anteromedial thalamic nuclei (Taube, 1995; Tsanov et al., 2011; Jankowski et al., 2015; for review see Clark and Harvey, 2016); in parahippocampal regions such as the postsubiculum (PoS) (Taube et al., 1990a), medial entorhinal cortex (MEC), and parasubiculum (PaS) (Sargolini et al., 2006; Boccara et al., 2010); and in dorsal cortical regions such as the parietal cortex (PC) (Chen et al., 1994a, b; Wilber et al., 2014; reviewed in Clark et al., 2018).

Several studies have reported that simultaneously recorded populations of HD cells tend to maintain coherence across their preferred firing directions (Taube et al., 1990b; Taube, 1995; Johnson et al., 2005; Peyrache et al., 2015; Bassett et al., 2018). For example, Taube et al. (1990b) recorded pairs of HD cells in the PoS simultaneously and found that cells responded similarly, with the same angular relationship with one another, across a broad range of environmental manipulations and testing procedures. Coherence between HD cells has also been reported across the ATN and PoS (Peyrache et al., 2015) and between HD cells in the ATN and place signals within the hippocampal formation (Knierim et al., 1998; Hargreaves et al., 2007). However, a recent study suggests that the coherence of HD cell populations recorded from the mouse MEC and PaS may become uncoupled during some environmental cue manipulations (Kornienko et al., 2018). Another previous study subjectively noted that decoding accuracy by ATN HD cell populations is superior to PoS HD cell ensembles (see Supplementary Figure S1 in Viejo et al., 2018). We are unaware of other studies that have quantified the accuracy of HD cell population coding across thalamo-cortical circuitry.

Although a number of methods have been developed to assess the dynamics of thalamo-cortical HD signals (e.g., Rybakken et al., 2018; Viejo et al., 2018; Fresno et al., 2019), few studies have conducted a quantitative comparison of neural decoding. Statistical model-based approaches have generally been favored with respect to studying population activity of the HD cell system, however recent advances have stimulated new interest in using machine learning approaches for neural decoding. Model-based methods directly characterize a probabilistic relationship between neural firing and HD, while machine-learning approaches assume a “black-box” neural network to describe the relationship. Although machine learning methods can in general deal with complex relationships in datasets, they depend on a multi-layered structure and come with a significant time cost.

A central aim of the present study was to provide a comparison of the various methods used to assess the neural dynamics of spatial behavior. Specifically, we compare linear methods such as Kalman Filter, Vector Reconstruction, Optimal Linear Estimator, and Wiener Filter and non-linear methods such as Generalized Linear Models and Wiener Cascade. We compare these statistical model-based methods with several machine learning methods. In addition, we present a quantitative assessment of population coding by HD cells within the ATN, PoS, PaS, MEC, and PC and explore contributing variables to decoding accuracy such as the number of classified HD cells per dataset as well as the firing rate and tuning strength of HD cell populations.

## Materials and Methods

### Datasets

Neuronal recordings analyzed in the present report were presented in previous work (Wilber et al., 2014, 2017; Winter et al., 2015a, b; Butler and Taube, 2017). Briefly, for data collected in ATN, PoS, PaS and MEC, 4 female Long-Evans rats (3–6 months of age) were used (5 recording sessions or datasets/region; 1–2 rats/region). Rats were either surgically implanted with moveable microdrives containing four tetrodes targeting the PoS, PaS, or MEC (Winter et al., 2015a, b), or eight individually moveable stereotrodes targeting the ATN (Butler and Taube, 2017). Neural activity in PoS, PaS, or MEC was recorded while animals foraged for scattered food in a large square enclosure (120 × 120 cm; 50 cm in height; session duration: 10–20 min) and in the ATN while rats foraged in a small cylindrical environment (71 × 50 cm; session duration: 8 min).

For data collected in PC, 4 male Fisher-Brown Norway hybrid rats were used. Rats were 5–10 months of age at initial surgery and were implanted with an 18-tetrode electrode array targeting the PC (for details see Wilber et al., 2014). Recordings were conducted while rats performed a “random lights” task in which the animal visited one of 32 light/reward zone located along the perimeter of a large circular open field (4 ft in dia). Each zone was rewarded with medial forebrain stimulation. Animals made up to 900 light/reward zones visits in a single recording session (session duration: ∼45 min). Each visit to the light/reward zone consisted of the animal making a trajectory from one end of the open field to the other. Because the light/reward zones were presented in a random order, the animal’s cumulative path for each session resulted in wide spatial and HD coverage in the environment. These experiments were carried out in accordance with protocols approved by the University of Lethbridge Animal Welfare Committee or Dartmouth College’s Institutional Animal Care and Use Committee and conformed to the National Institutes of Health *Guide for the Care and Use of Laboratory Animals*.

For all datasets, electrical signals were pre-amplified on a headstage (HS18 or HS27) and were recorded using a Digital Lynx Data Acquisition System (Neuralynx, Bozeman, MT), and thresholded (adjusted prior to each session) spike waveforms (filtered 0.6–6 kHz, digitized at 32 kHz) and timestamps were collected for each session. Rat position and HD were tracked by either using red and green LEDs attached to the animal’s headstage (secured ∼8 cm apart) or by using colored domes of reflective tape which were created by covering 1/2 Styrofoam balls in reflective tape. A video tracking system provided x-y coordinates of each LED or Styrofoam ball position at a sampling rate of 30–60 Hz as interleaved video. However, for one animal included in the PC datasets, data was collected at 30 Hz (rat 4) and co-registered with spikes and stimuli.

For PoS, PaS, MEC, and ATN datasets, spike sorting was conducted using SpikeSort3D (Neuralynx, Bozeman, MT). First, waveform characteristics from each tetrode/stereotrode were plotted as scatterplots from one of the four tetrode wires and signal waveform characteristics (amplitude, peak and valley) were used for cell isolation. Individual units formed clusters of points in these plots and the boundaries were identified and manually “cut.” For PC datasets, spike data were automatically overclustered using KlustaKwik^1^ then manually adjusted using a modified version of MClust (A.D. Redish).

### HD Cell Categorization

#### ATN, PoS, PaS, and MEC Recordings

The HD of the animal was determined by the relative position of the red and green LEDs. The amount of time and number of spikes in each HD was sorted into sixty 6° bins. The firing rate for each 6° bin was determined by dividing the number of spikes by the amount of time. A firing rate by HD plot was constructed for each cell in the dataset and the directionality of each cell was quantified using a number of measures. First, we computed the mean vector length (Rayleigh r) for each cell. The mean vector length ranges between 0 and 1, with higher values indicating that spike occurrence is clustered around a particular direction. Second, we computed a stability score for each cell. Stability was calculated by dividing the recording session into four equal time bins and cross-correlating the 60 directional firing bins across each time bin and averaging these values (Directional Stability = (Q1:Q2 + Q1:Q3 + Q1:Q4 + Q2:Q3 + Q2:Q4 + Q3:Q4)/6). Because the mean vector length is susceptible to reporting high values when cells display low firing rates, we used a dual criterion for classifying neural activity as an HD cell. Cells were classified as an HD cell if the resulting mean vector length and directional stability scores exceeded the 95th percentile chance level generated by shuffling the neural data (see Boccara et al., 2010; Winter et al., 2015a). Briefly, each cell had its sequence of spikes time-shifted relative to the animal’s tracked location and HD (400 iterations for each cell) and the mean vector length and stability was calculated for each iteration. The 95th percentile value for each region was taken as the cut-off criteria for cell inclusion. In addition, cells with criteria values below the mean cutoff across brains regions, without clear directionality and repeat recordings were removed from further analysis. A sample of 5 recording sessions or datasets per brain region was selected. Each dataset contained at least 5 simultaneously recorded HD cells that met the criteria outlined above (*n* = 20 datasets from 4 rats).

#### PC

Cells not sufficiently active during maze sessions (< 250 spikes/session; session = ∼50 min) were excluded from all analyses (39 cells excluded so 339 putative pyramidal cells remained). Data from video frames in which HD tracking was lost or segments in which the rat was still for relatively long (60 s) periods (calculated from smoothed positioning data) were excluded. Occupancy data were binned per 6° of HD and converted to firing rate (spikes/s). Rayleigh statistics were calculated using a combination of custom Matlab scripts and the circular statistics toolbox (Berens, 2009). Because directionally modulated PC cells typically expressed low firing rates across behavioral testing, we adjusted the HD cell classification criteria to assess stability across a longer recording duration. Thus, neurons were classified as HD cells if (1) they had a significant Rayleigh test for unimodal deviation from a uniform distribution corrected for binned data on the collapsed-across-behavioral-sessions firing rate data (*p ≤* 0.05) and (2) they were stable (change in peak vector direction of < 7 bins) across behavioral sessions (or split 1/2 sessions when data were not available for two consecutively recorded sessions). All datasets for which at least 3 HD cells met these criteria were included in the present paper (*n* = 7 sessions from 3 rats; 2 session from rat #1; 2 sessions from rat #3; 3 sessions from rat #4).

### Neural Decoding Methods

Twelve decoding methods were applied. Six are statistical model-based methods: Kalman Filter, Generalized Linear Model, Vector Reconstruction, Optimal Linear Estimator, Wiener Filter and Wiener Cascade. The remaining six are machine learning methods: Support Vector Regression, XGBoost, Feedforward Neural Network, Recurrent Neural Network, Gated Recurrent Unit, and Long Short-Term Memory. The python code for Wiener Filter, Wiener Cascade and the machine learning methods is from the freely available Neural Decoding package from Glaser et al. (2017)^2^. Head direction data were transformed using directional cosines, then fed into the decoding algorithm, then transformed back to polar coordinates (Gumiaux et al., 2003; Wilber et al., 2014, 2017). For better explanatory power, a four-fold cross-validation is applied in this paper. Since the data have a time series structure and so do the models, it was not appropriate to use a middle portion as testing where the training data is not continuous. Thus, we only included two cases: upper 3/4 of the dataset to be training (UT) and lower 3/4 of the dataset to be training (LT).

#### Statistical Model-Based Methods

##### Kalman Filter

The Kalman Filter model (Kalman, 1960) is a hidden Markov chain model that uses HD (trigonometric) as the states and spike counts as the observations.

**FIGURE 1:**
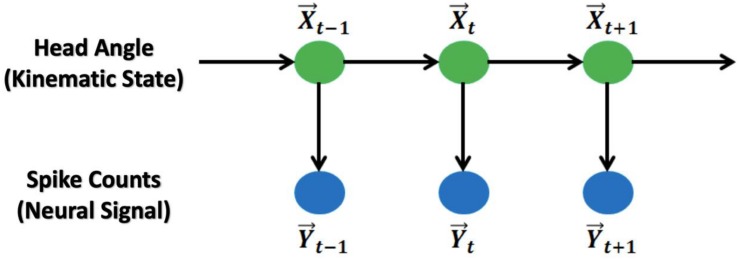
Graphic representation of the Kalman Filter and Generalized Linear Model: The main model is a hidden Markov chain structure. HDs follow a Markov chain and spike counts at the current time bin are independent from the counts from previous time bins.

The relationship between these variables is shown in Figure 1.

The model assumes that the HD follows a first-order auto-regression structure with additive Gaussian noise. The model is given as:

$$\left\{ \begin{array}{c} {\vec{X}}_{t+1}=A\,{\vec{X}}_{t}+{\vec{w}}_{t}\\ {\vec{Y}}_{t}=H\,{\vec{X}}_{t}+{\vec{q}}_{t} \end{array}\right.$$

where ${\vec{X}}_{t}$ is the centralized trigonometric HD vector (centralized [cos, sin] vector) at time *t*; ${\vec{Y}}_{t}$ is the centralized spike counts vector for all observed brain cells at time *t*; ${\vec{w}}_{t}$, ${\vec{q}}_{t}$ are the random noises where ${\vec{w}}_{t}∼N\,\left(0,W\right)$, ${\vec{q}}_{t}∼N\,\left(0,Q\right)$, and ${\vec{w}}_{t},{\vec{q}}_{t}$ are independent. The Kalman Filter method assumes a mean of zero for the noise model, so, the mean spike count must be subtracted from the neural data, i.e., we “centralized the spike counts.” Note that since ${\vec{X}}_{t}$ and ${\vec{Y}}_{t}$ are centralized, no intercept term is included in the model.

For parameter fitting, the classical approach, maximum likelihood method (MLE) is used to obtain the values of *A*,*W*,*H*,*Q* (see Supplementary Material S1). For decoding, the Kalman Filter algorithm (Wu et al., 2006) is applied to predict ${\vec{X}}_{t}$ given ${\vec{Y}}_{t}$ after the estimation of model parameter (see the algorithm in Supplementary Material S2).

##### Generalized linear model

Similar to the Kalman Filter model, the generalized linear model is also a hidden Markov Chain model with HD (trigonometric) as the states and spike counts as the observations (Figure 1). The model assumptions are: (1) the HD itself is a first-order autoregression model with additive Gaussian noise; (2) the HD and spike counts at the same time point follow a Poisson log-linear model; and (3) the spike counts from each observed brain cell are conditional independent given the HD at the same time point. The model is:

$$\left\{ \begin{array}{c} {\vec{X}}_{t+1}=A\,{\vec{X}}_{t}+{\vec{w}}_{t}\\ Y_{t,c}|{\vec{X}}_{t}∼P\,o\,i\,s\,s\,o\,n\,\left(\lambda_{t,c}\right) \end{array}\right.$$

Where ${\vec{X}}_{t}$ is the centralized trigonometric HD vector at time *t*; *Y*_*t*,*c*_ is the spike counts for brain cell *c* at time *t* and ${\left\{Y_{t,c}\right\}}_{c=1}^{C}$ is independent given ${\vec{X}}_{t}$; ${\vec{w}}_{t}$ is random noise with ${\vec{w}}_{t}∼N\,\left(0,W\right)$, $\lambda_{t,c}=e^{\mu_{c}+{\vec{a}}_{c}^{T}\,{\vec{X}}_{t}}$. Similarly, there is no intercept term in the autoregression model because ${\vec{X}}_{t}$ has been centralized.

To fit the model parameters $A,W,\mu_{c},{\vec{a}}_{c}$, we again use the maximum likelihood method (Lawhern et al., 2010; see Supplementary Material S3). For decoding, the Point Process Filter method (Eden et al., 2004) is applied to predict the HD given the spike counts (see the algorithm in Supplementary Material S4). Based on the model, the mean of *Y*_*t*,*c*_ given ${\vec{X}}_{t}$ can be numerically approximated by ${\hat{\lambda }}_{t,c}=e^{{\hat{\mu }}_{c}+{\hat{\vec{a}}}_{c}^{T}\,{\vec{X}}_{t}}$ after parameter estimation, so the mean curve of spike counts among different HDs can be obtained.

##### Vector reconstruction

Since the training dataset contains the HD and spike counts at each observed time bin, we can make an estimation of the preferred direction for each cell (Georgopoulos et al., 1983). The estimation is done by fitting a cosine curve to the plot of firing rate and HD from the training data. The angle at the peak of the curve, which is the phase of the cosine function, is treated as the angle of the fitted preferred direction for the cell. In other words, ${\hat{\vec{L}}}_{c}={\left[\cos\,\hat{\theta },\sin\,\hat{\theta }\right]}^{T}$ will be the fitted unit direction vector for cell *c*. The prediction of the HD given firing rates can then be obtained by computing the average of the direction vectors, weighted by the corresponding cell’s firing rate, as in Johnson et al. (2005).

$$\phi_{\mathrm{est}}\,\left(t\right)=\mathrm{angle}\,\left[\sum_{c=1}^{C}f_{c}\,\left(t\right)*{\hat{\vec{L}}}_{c}\right]$$

Where *f*_*c*_(*t*) is the given firing rate of cell *c* at time *t*; ${\hat{\vec{L}}}_{c}$ is the fitted preferred direction vector for cell *c*; ϕ_est_(*t*) is the predicted HD at time *t*;angle(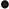) returns the angle of the input vector (see the computation in Supplementary Material S5).

**FIGURE 2:**
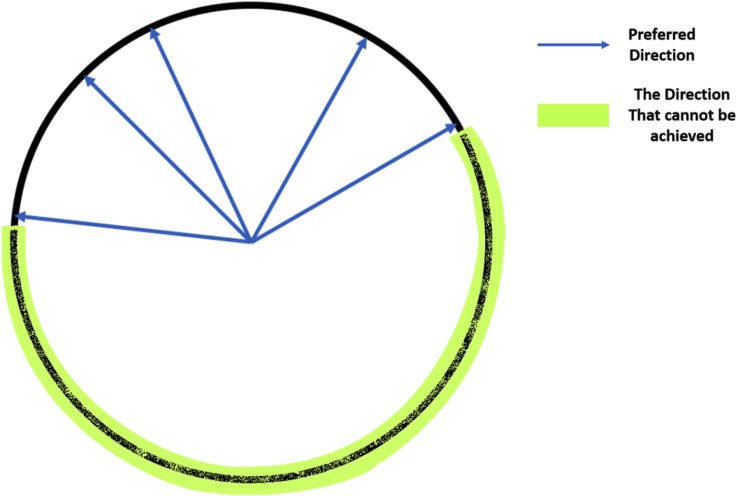
Illustration of the coverage of the full range of HDs: If all the preferred direction vectors cover only half of the possible HDs, then the vectors in the other half circle cannot be achieved by a non-negative weighted linear combination of these vectors, so the predicted angles will not cover all values between 0° and 360°.

To achieve an accurate reconstruction with this method, there are several critical criteria for the training dataset. First, the data should have a sufficiently strong unimodal peak for a specific HD and firing rate, or else the estimation of preferred directions will be poor. Second, the preferred direction vectors must cover the full range of directions from 0° to 360°. Without input data covering some HDs, some predicted HDs may never be achieved (see Figure 2).

##### Optimal linear estimator

The Optimal Linear Estimator (OLE) method (Salinas and Abbott, 1994) is similar to the vector reconstruction method that estimates a direction vector for each cell and uses the weighted average over those vectors with firing rates as the weights to make a prediction of the HD. Since Vector Reconstruction and Optimal Linear Estimator both depend on the preferred direction vector (unlike other decoding methods), these methods are especially susceptible to inhomogeneity of preferred directions as illustrated in Figure 2. The vector, ${\hat{\vec{D}}}_{c}$ for cell *c*, unlike the preferred direction vector, is obtained by finding the optimal solution that minimizes the squared error between the estimated and true HD vector, averaged over firing rates and true direction vectors, i.e.

$$\hat{D}=\underset{D}{a\,r\,g\,m\,i\,n}\int {\left(\vec{V}-{\vec{V}}_{\mathrm{est}}\right)}^{2}*P\,\left(\vec{r}|\vec{V}\right)\,d\,\vec{r}\,d\,\vec{V}$$

Where $\vec{V}$ is the true HD vector; ${\vec{V}}_{\mathrm{est}}=\sum_{c=1}^{C}r_{c}*{\vec{D}}_{c}$ is the estimated HD vector; $\vec{r}={\left[r_{1},r_{2},\ldots \,r_{C}\right]}^{T}$ is the firing rate for each brain cell; $D=\left[{\vec{D}}_{1},{\vec{D}}_{2},\ldots \,{\vec{D}}_{C}\right]$ is the matrix of all the vectors to find.

The solution for $\hat{D}$ can be computed by taking the derivative of the formula above, which results in:

$${\hat{\vec{D}}}_{c}=\sum_{i=1}^{C}{\left({\hat{Q}}^{-1}\right)}_{\mathrm{ci}}*{\hat{\vec{L}}}_{c}$$

Where ${\hat{\vec{L}}}_{c}$ is the numerical approximation of the center of mass vector for the tuning curve function of cell *c*; $\hat{Q}$ is the numerical approximation of the correlation matrix of firing rates for all cells (see details in Supplementary Material S6).

With fitted $\hat{D}$, the prediction of the HD at time *t*, as stated above, is

$$\phi_{\mathrm{est}}\,\left(t\right)=\mathrm{angle}\,\left[\sum_{c=1}^{C}r_{c}\,\left(t\right)*{\hat{\vec{D}}}_{c}\right]$$

The OLE method also has the prerequisite on the training dataset like the vector reconstruction method that the non-negative linear combination of ${\hat{\vec{D}}}_{c}$ should cover all directions from 0° to 360°, or else the prediction can never achieve some angles (see Figure 2).

##### Wiener Filter

The Wiener Filter model (Wiener, 1949) is a classical regression method that builds a multiple linear regression relation between the HD and the firing rate from every observed cell at the corresponding time bins. This model is the basis of all the statistical filtering methods. Computational details are given in Supplementary Material S7.

##### Wiener cascade

The Wiener Cascade model (Hunter and Korenberg, 1986) is a direct extension of the Wiener Filter model that first applies a multiple linear regression model on the HD vs. the firing rate from each cell, and then builds a non-linear model on the fitted values from the linear model vs. the true firing rate values. In the present paper, the order of the polynomial in the non-linear component was searched by Bayesian Optimization (see section Machine Learning Methods below). Computational details are also given in Supplementary Material S7.

#### Machine Learning Methods

To conduct HD decoding, we also used the following 6 machine learning methods. The selection on input-output is consistent for each method. In these methods, together with Wiener Cascade, there exists some free parameters that are not tuned during training. Instead they are set before the optimization process. These values are called “hyper-parameters.” In this paper, hyper-parameter selection was based on Bayesian Optimization (Snoek et al., 2012, freely available python package^3^). It searched over a range of values for the hyper-parameters and chose the optimal one. Further detail is provided in Supplementary Material S8.

##### Support vector regression

The support vector regression (Drucker et al., 1996) is a machine learning tool that uses a non-linear kernel to project the input to another space and then builds a linear model on the projected input and the output. In this manuscript, a radial basis function kernel was applied. The penalty parameter of the error term and the maximum number of iterations were searched by Bayesian Optimization.

##### XGBoost

XGBoost (Chen and Guestrin, 2016) is a machine learning algorithm that implements the idea of gradient boosted trees. It builds a sequence of regression trees. The first tree is for predicting the HDs using the firing rates, while each subsequent tree is built on the firing rate vs. the residual of the previous fit. In this manuscript, the total number of trees, maximum depth of each tree and the learning rate were all searched by Bayesian Optimization.

##### Feedforward neural network

**FIGURE 3:**
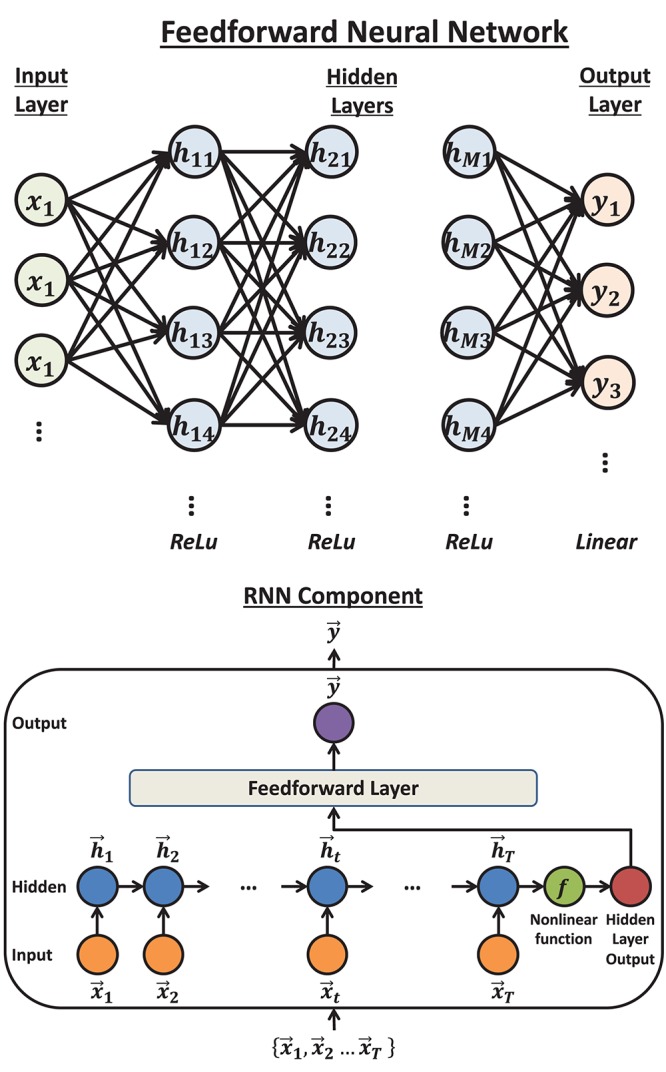
The structure of feedforward neural network and Recurrent Neural Network (RNN): **Top:** The typical structure of a feedforward neural network. Each unit will calculate a weighted sum of the units in the previous layer that connect to it by an arrow. Then by adding an intercept term and transforming the value by an activation function, the unit obtains the value it sends out. **Bottom:** the structure of a Recurrent Neural Network component. The input vectors are connected by a chain hidden layer. Each hidden unit is the transformed value of the linear combination of the corresponding input unit and previous hidden unit. The last hidden unit value (vector) will be transformed by another non-linear function and sent to the dense layer to compute the output.

The feedforward neural network (Haykin, 1994), also called dense neural network or multi-layer perceptron, is the basic structure in deep learning. In this method, each two consecutive layers are fully connected, which means that every unit in the subsequent layer will be computed by a linear function on the values from all the units in the previous layer, followed by an activation function (Figure 3 *Top*). In the present paper, 3 hidden layers were used. The activation functions were rectified linear unit, abbreviated as ReLU (Glorot et al., 2011), for all the hidden layers and linear for the output layer. To avoid overfitting, we applied the dropout method (Srivastava et al., 2014). The optimization algorithm was *Adam* (Kingma and Ba, 2014). The number of units in the layers, the dropout rate and the number of epochs were all searched by Bayesian Optimization.

##### Recurrent Neural Network

The Recurrent Neural Network is the basic neural network structure designed for time series data (Haykin, 1994). A Recurrent Neural Network component includes one hidden layer, where each unit is a linear combination of the values from the corresponding input unit and the previous hidden unit. The last hidden unit value is then transformed by a non-linear function and finally fully connected to the output layer (Figure 3 *Bottom*). In this paper, a series of Recurrent Neural Network components were applied so that each component predicts the HD in one time bin, given the firing rates. The non-linear function was set to ReLU. Similar to feedforward neural network, the dropout method was applied. The optimization algorithm was chosen to be RMSprop (Tieleman and Hinton, 2012). The dimension of the hidden unit, the dropout proportion and the number of epochs were searched by Bayesian Optimization.

##### Gated recurrent unit

**FIGURE 4:**
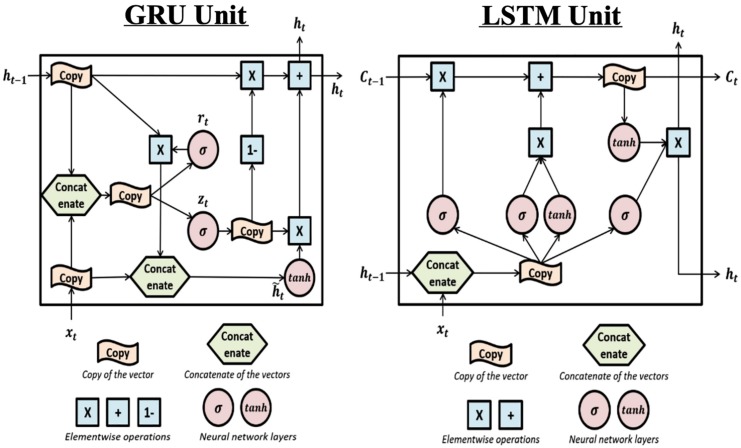
The structure of Gated Recurrent Unit and Long Short-Term Memory units: **Left:** this is the structure of the Gated Recurrent Unit (GRU). The “update gate” z_*t*_ is used to determine if the update ${\tilde{\text{h}}}_{t}$ will be applied to h_*t*_. r_*t*_ is the “reset gate” and is used to determine if the previous hidden value (also the output value) h_*t*__–__1_ will be kept in the memory. The effects of the two gates are achieved by sigmoid activation functions which can be learned during training. **Right:** The structure of the Long Short-Term Memory (LSTM) unit. LSTM is complex and includes one more hidden value C_*t*_ and more gates compared to GRU. Each gate can be seen in the plot where the **σ** sign appears (i.e., sigmoid activation function). The first **σ** is the “forget gate” which controls whether previous hidden value, C_*t*__–__*v*_ will be used to calculate current output and kept in the memory. The second **σ** is the “input gate” which controls whether the new input will be used to calculate current output. The third **σ** is the “output gate” which filters the output, i.e., controls what part of the output values to send out as h_*t*_.

The gated recurrent unit (Cho et al., 2014) is a complex recurrent neural network unit. Its structure (shown in Figure 4 *Left*) is similar to the Recurrent Neural Networks but includes gated units which can better memorize the long-term history.

In this paper, the use of Gated Recurrent Unit (GRU) was the same as the hidden units in the Recurrent Neural Network (RNN) component. The GRU component was a chain structure of several gated units and it was applied to predict HD in one time bin. The model also applied the dropout method to avoid overfitting and used RMSprop to be the optimization algorithm. Same as the RNN methods, the dimension of gated units, the dropout proportion and the number of epochs were searched by Bayesian Optimization. An implementation difference was that the activation function between the output from the recurrent part and input to the feedforward layer was hyperbolic tangent (tanh) instead of ReLU since the former is the standard choice for Gated Recurrent Unit.

##### Long short-term memory

The Gated Recurrent Unit and Long Short-Term Memory (Hochreiter and Schmidhuber, 1997) were developed from Recurrent Neural Network and can better handle the long-term dependencies (the structure is shown in Figure 4 *Right*).

Compared to the Gated Recurrent Unit, the Long Short-Term Memory unit has a more complex structure which includes more parameters. In the present paper, the use of Long Short-Term Memory was just a replacement of the Gated Recurrent Unit with the same settings: optimization algorithm = RMSprop; activation non-linear function = tanh; dimension of LSTM components, dropout proportion and number of epochs were searched by Bayesian Optimization.

### Statistical Analyses

Data were analyzed using two-way repeated measures ANOVAs (e.g., Decoding Method or Brain Region). In order to avoid large numbers of pairwise post-tests, we determined which factors were contributing to significant ANOVA results by removing factors one at a time. We started with the factor that was furthest from the mean, removed it, and reran the ANOVA. We repeated this process until the ANOVA was no longer significant. We also explored factors that may contribute to variability in decoding accuracy including the number of classified HD cells per dataset, cell firing rate, HD tuning strength, and angular head velocity (described in section Factors Influencing Variability Across Decoding Method, Brain Region, and Datasets). Linear regression was used to compare decoding accuracy to each of these factors. For all statistical analyses, *p* < 0.05 was considered significant and Matlab statistics toolbox was used for statistical analyses (Mathworks). Rayleigh statistics were calculated using a combination of custom Labview and Matlab scripts using the circular statistics toolbox (Berens, 2009).

## Results

As described in section Neural Decoding Methods, cross-validation has been applied. There are two cross validation approaches: UT and LT. After running the code for all datasets, the results of the two cases is consistent. For brevity, only the results for UT are displayed. The output for LT can be seen in Supplementary Material S13.1–S13.12.

### Neural Decoding

#### Modeling Tuning Properties

**FIGURE 5:**
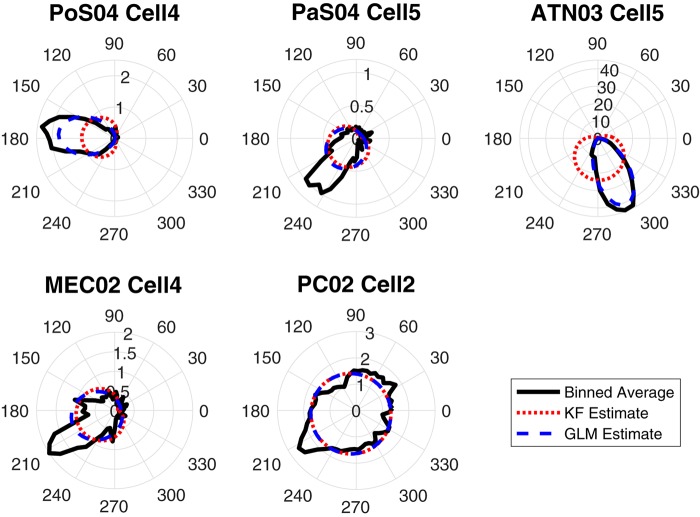
The true-vs.-estimated tuning plots in 6-degree bins for one HD cell in each brain region: The polar plots show firing rates vs. HD. The black curves are the true tuning functions, smoothed by a Gaussian kernel function. The red curves are the estimated functions using the Kalman Filter (KF) method and the blue curves are the estimated functions using the Generalized Linear Model (GLM) method.

Some decoding approaches use a likelihood model (i.e., firing rate given HD) in a Bayesian framework to represent individual single units. Two of the twelve methods we used, the Kalman filter and Generalized Linear Model, use likelihood representations. An examination of the likelihood representations is useful for understanding successful (and unsuccessful) decoding of HD. Thus, to compare the approaches, we first produced tuning curve plots (i.e., polar plots) showing the relationship between the cells firing rate and the animal’s HD (Figure 5; black curves). The modeling result is overlaid on the firing rate polar plots (blue curves: Generalized Linear Model estimation, red curves: Kalman Filter estimation). One can roughly assess the model fitness for these two methods by visually comparing the similarity between the estimated tuning curve and the true tuning curve. By comparing the black (true), blue (Generalized Linear Model) and red (Kalman Filter) curves, it is apparent that Generalized Linear Model estimations are more similar to the true curves compared to the Kalman Filter estimations. The poorer performance of the Kalman Filter is likely a consequence of the model assumption. Specifically, the Generalized Linear Model proposes a Poisson distribution on the discrete spike count, which is more appropriate than the Gaussian distribution assumed by the Kalman Filter model.

#### Decoding Output

**FIGURE 6:**
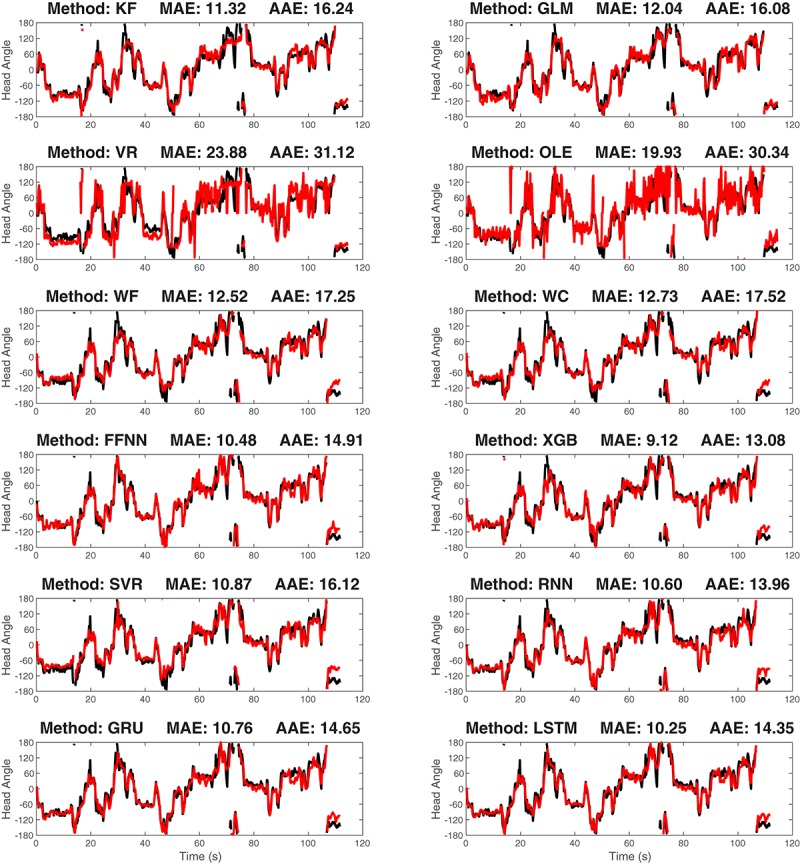
The true-vs.-predicted head angle plotted as a function of time for a representative ATN dataset for each of the 12 decoding methods: The black curves are the true curves and the red curves are the predicted curves. Test data is shown. Predicted curves are constructed using a model generated from a separate training segment of the data. The method name and decoding accuracy measured as median absolute error (MAE) are shown on the title of each plot (average absolute error, AAE, is also shown). KF, Kalman Filter; GLM, Generalized Linear Model; VR, Vector Reconstruction; OLE, Optimal Linear Estimator; WF, Wiener Filte, and WC, Wiener Cascade. The remaining six are machine learning methods: SVR, Support Vector Regression; XGB, XGBoost; FFNN, Feedforward Neural Network; RNN, Recurrent Neural Network; GRU, Gated Recurrent Unit; LSTM, Long Short-Term Memory.

After training the model, we decoded the HD for the validating data and contrasted the decoding result with the true values. As a first-step, we visually compared the true and reconstructed HD as a function of time (Figure 6). This revealed that while some approaches are more accurate than others, all approaches were capable of producing at least moderately accurate decoding.

Next, we quantified decoding accuracy by calculating the median absolute error (*MAE*) and comparing this measure across datasets and brain regions. The median absolute error is computed by taking the circular difference between the predicted and true angle, rescaling the angle difference to be within [−180°, 180°], taking the absolute value of this angle, and then calculating the median value. For instance, if the true angle is 10° and the predicted angle is 350°, then the absolute difference after rescaling is 20°. The median absolute error, *MAE*, is:

$$\mathrm{MAE}=\underset{t=1,2\,\ldots \,T}{m\,e\,d\,i\,a\,n}\left|\mathrm{rescale}\,\left[\phi \,\left(t\right)-\phi_{\mathrm{est}}\,\left(t\right)\right]\right|$$

where ϕ(*t*) is the true valid HD at time *t*, ϕ_est_(*t*) is the predicted HD at time *t*, rescale(θ) is the function that changes the angle θ to be within [−180°, 180°].

For comparison, we also computed the average absolute error (*AAE*). Compared to the median, the average is much more sensitive to outliers and extreme values, so the *AAE* values turn out to be larger and not as stable as the *MAE*. As a result, we used the *MAE* as the measure of decoding performance for the main text in this paper. The result associated with *AAE* can be seen in Supplementary Material S14.1–S14.16.

*MAE* is negatively related to prediction accuracy such that a smaller *MAE* indicates better prediction accuracy. Twenty-seven datasets from 5 brain regions were decoded using each of the 12 methods. The *MAE* was calculated for each dataset and method. All values and dataset details are shown in the Supplementary Material S9.1.

**FIGURE 7:**
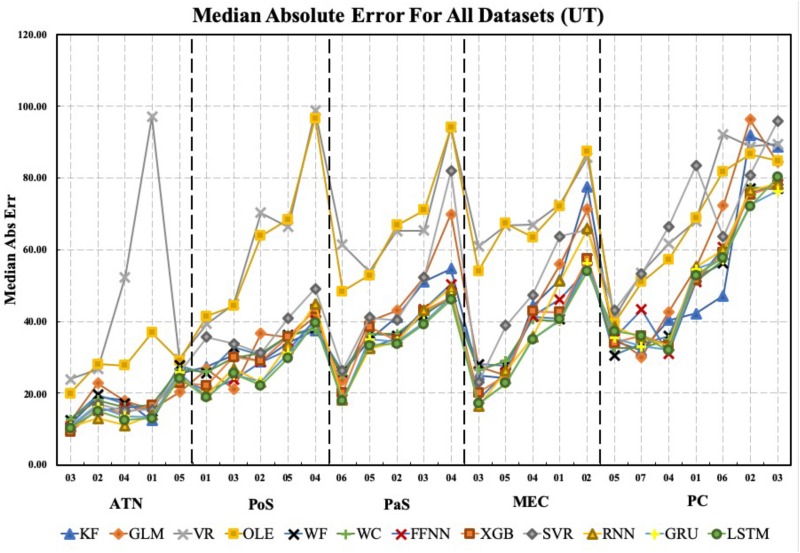
The median absolute error is shown for each brain region, each dataset, and each decoding method. Datasets for each brain region are sorted from lowest to highest median absolute error (i.e., from best to worst decoding accuracy). Note that median absolute error varies considerably within regions and on average increases from ATN to parahippocampal and PC regions. KF, Kalman Filter; GLM, Generalized Linear Model; VR, Vector Reconstruction; OLE, Optimal Linear Estimator; WF, Wiener Filter; WC, Wiener Cascade; SVR, Support Vector Regression; XGB, XGBoost; FFNN, Feedforward Neural Network; RNN, Recurrent Neural Network; GRU, Gated Recurrent Unit; LSTM, Long Short-Term Memory.

Figure 7 shows the *MAE* for each method, brain region, and dataset. Notably, the Vector Reconstruction method and Optimal Linear Estimator methods produced larger *MAE* compared to other methods. The LSTM method had the smallest average *MAE* value (34°). Regardless of the decoding method, *MAE* varied dramatically across datasets and brain regions. The average decoding accuracy was greatest in ATN, with datasets from this region expressing the lowest measures of *MAE*. For parahippocampal cortex and PC, *MAE* was greater relative to ATN, and progressively increased in a topographical manner from POS < PaS < MEC < PC. Finally, within each brain region, *MAE* values varied substantially for different datasets. For example, PC datasets PC_02 and PC_03 have much larger *MAE* values than the other PC datasets. This suggests that other factors in addition to regional differences may contribute to variability in decoding accuracy.

### Decoding Accuracy as a Function of Computational Method and Brain Region

#### Decoding Accuracy Across Computational Methods

**FIGURE 8:**
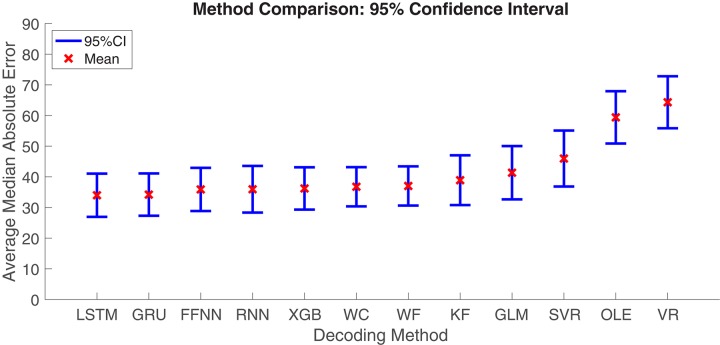
Mean ± 95% Confidence-Interval (CI) Median Absolute Error (MAE) for each decoding method. Data from different brain regions and datasets were pooled. KF, Kalman Filter; GLM, Generalized Linear Model; VR, Vector Reconstruction; OLE, Optimal Linear Estimator; WF, Wiener Filter; WC, Wiener Cascade; SVR, Support Vector Regression; XGB, XGBoost; FFNN, Feedforward Neural Network; RNN, Recurrent Neural Network; GRU, Gated Recurrent Unit; LSTM, Long Short-Term Memory.

Next, we aimed to quantify the variance observed across decoding methods. The Optimal Linear Estimator method and Vector Reconstruction method appear to have large error relative to the other 10 methods (see Figure 7). Therefore, we compared *MAE* values collapsed across datasets and brain regions. As expected, we found that decoding accuracy varied significantly across computational methods [*F*_(__11_, _312__)_ = 7.27, *p* < 0.001; Figure 8]. Next, to determine which methods were contributing to this variance, we removed data from one method at a time starting with the method furthest from the mean (Vector Reconstruction) and repeated the ANOVA until a non-significant result was obtained (see section Materials and methods). It was necessary to remove both Vector Reconstruction and Optimal Linear Estimator methods before decoding accuracy no longer varied significantly across method [*F*_(__9_, _260__)_ = 1.00, *p* = 0.44], suggesting that decoding accuracy is similar for the remaining 10 methods. Potential causes of the poor performance for the Vector Reconstruction and Optimal Linear Estimator methods are explored in the section Factors Influencing Variability Across Decoding Method, Brain Region, and Datasets.

#### Decoding Accuracy Varied Across Brain Regions

**FIGURE 9:**
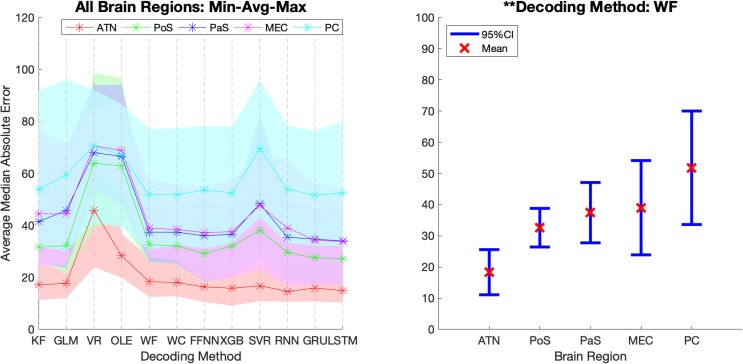
Decoding accuracy varies across brain regions. The average Median Absolute Error (MAE) for each area and each decoding method. The shading in the **left panel** represents the range of the MAE values while the error bars in the **right panel** represents the 95% Confidence-intervals of the average MAE values for a representative decoding method. 95% Confidence-interval plots for the remaining 11 methods are shown in Supplementary Material S10. KF, Kalman Filter; GLM, Generalized Linear Model; VR, Vector Reconstruction; OLE, Optimal Linear Estimator; WF, Wiener Filter; WC, Wiener Cascade; SVR, Support Vector Regression; XGB, XGBoost; FFNN, Feedforward Neural Network; RNN, Recurrent Neural Network; GRU, Gated Recurrent Unit; LSTM, Long Short-Term Memory; ATN, Anterior Thalamic Nuclei; PoS, Postsubiculum; PaS, Parasubiculum; MEC, Medial Entorhinal Cortex; PC, Parietal Cortex. ^∗∗^*p* < 0.01.

In addition to variability across decoding methods, we observed variance in *MAE* across the 5 brain regions (see Figure 7). It is visually apparent that *MAE* is topographically organized such that the measure progressively decreases from PC > MEC > PaS > PoS > ATN, however, there is considerable overlap between the decoding accuracy across these brain regions (Figure 9). We therefore quantified the effect of brain region for each decoding method and collapsed across datasets. We found that for most methods (11/12), accuracy significantly varied across brain region [Figure 9 and Supplementary Material S10; *F*_(__4_, _22__)_ > 2.82, *p* < 0.05], with the exception of Vector Reconstruction [*F*_(__4_, _22__)_ = 1.27, *p* = 0.31]. Further, for the 11 methods with significant variance across brain region, ATN accuracy was highest and furthest from the mean. For 9 of the methods, removing ATN resulted in a non-significant ANOVA [*F*_(__3_, _18__)_ < 3.16, *p* > 0.05]. The only exceptions were Support Vector Regression and Long Short-Term Memory. For these methods, it was necessary to also remove the brain region that was the second furthest from the mean, PC [*F*_(__2,12__)_ < 3.89, *p* > 0.05].

We also investigated whether our findings above could be influenced by variability in the animal’s movement characteristics. We first measured whether there were significant biases in the animal’s trajectory by determining the dwell time in each HD. Plotting the data in this way demonstrates that good coverage of the full range of HDs occurred for all datasets from each brain region (Supplementary Material S11). We next measured the animals angular head velocity (absolute angular velocity calculated across 0.2 s time bins). The ANOVA determined that the absolute angular head velocity varied significantly across brain region [*F*_(__4_, _22__)_ > 6.814, *p* < 0.001]. When PC was removed, the ANOVA was no longer significant [*F*_(__3_, _16__)_ < 2.462, *p* > 0.1; consistent for both UT and LT datasets]. On average, fewer high velocity head movements were performed by rats in the PC datasets (mean: 41°/s). This observation is not entirely surprising given that animals in the PC datasets performed a task involving direct trajectories to a goal location (Wilber et al., 2014), which contrasts with the varied head movements made during random foraging in the other datasets (see section Datasets). Finally, a linear regression found that the relationship between the absolute angular head velocity and *MAE* was not significant for Kallman Filter, General Linear Model, and Optimal Linear Estimator methods (absolute value of the *r*s ≤ 0.27, *p*s ≥ 0.08), but was for all other methods (absolute value of the *r*s ≥ 0.34, *p*s < 0.05).

It should be noted that there are at least three additional variables that could influence our findings above. First, the density of HD cells varies considerably across brain regions (reviewed in Taube and Bassett, 2003; Taube, 2007). So, it is possible that some of the variability in decoding accuracy across brain regions may be an indirect result of HD cell density. We directly assess the potential influence of the number of cells on decoding accuracy below (Factors Influencing Variability Across Decoding Method, Brain Region, and Datasets). Second, a number of studies have observed that HD cells can vary in their peak firing rate and other firing characteristics that can influence the cells signal-to-noise ratio. Again, we evaluate these variables in the section below (Factors Influencing Variability Across Decoding Method, Brain Region, and Datasets). Last, it is important to note that different recording procedures, numbers of HD cells, behavior testing, and a distinct set of criteria were used for classification of HD cells for PC datasets. So, the slightly weaker decoding accuracy in PC could be attributed to one or all of these variables. Finally, the inclusion criteria for HD cells do not exclude cell firing which may correlate with HD but additionally fire relative to other spatial features such as egocentric bearing (Wilber et al., 2014; Peyrache et al., 2017).

#### Factors Influencing Variability Across Decoding Method, Brain Region, and Datasets

Next, we set out to explore factors that could underlie the variability we observed across brain regions and datasets (Figure 7). We identified three factors that could influence the decoding accuracy: the number of observed cells, the HD tuning strength, and the response rate of the cells.

##### Number of observed cells

**FIGURE 10:**
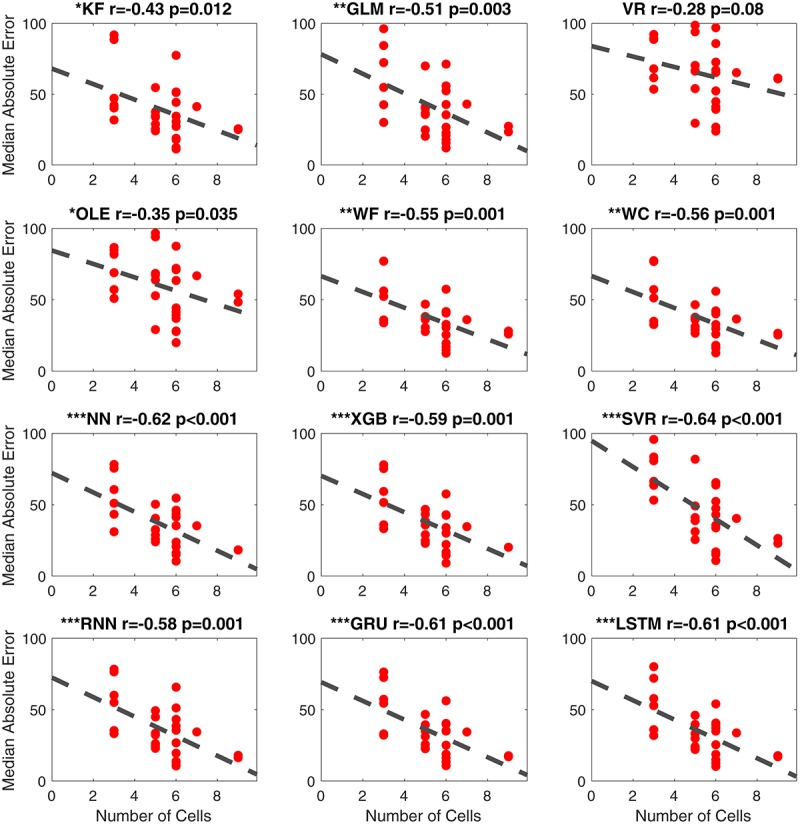
Scatterplots of median absolute error vs. number of cells for all 12 methods. The dashed line is the fitted linear regression. The correlation coefficient (r) and the corresponding *p*-value are shown on the top-right corner of each panel. The significance levels are shown with symbols on the top-left corner ^∗∗∗^*p* < 0.001; ^∗∗^*p* < 0.01; ^∗^*p* < 0.05. KF, Kalman Filter; GLM, Generalized Linear Model; VR, Vector Reconstruction; OLE, Optimal Linear Estimator; WF, Wiener Filter; WC, Wiener Cascade; SVR, Support Vector Regression; XGB, XGBoost; FFNN, Feedforward Neural Network; RNN, Recurrent Neural Network; GRU, Gated Recurrent Unit; LSTM, Long Short-Term Memory.

As noted above, the percentage of cells classified as HD cells varies among the different brain regions (Taube and Bassett, 2003; Taube, 2007). For instance, previous studies report that HD cells are most abundant in the ATN (∼60%; Taube, 1995) and slightly more sparse within PoS (∼25%; Taube et al., 1990a; Sharp, 1996) and in other cortical regions such as PC (∼12%; Wilber et al., 2014). Boccara et al. (2010) found large proportions of directionally modulated cell types in PoS, PaS, and MEC (53.7, 58.5, and 55.1%, respectively). In the present study, the density of HD cells varied from 3 to 9, which is within the range of cell densities reported in other studies using neural decoding methods (e.g., minimum of 3 cells/session in Johnson et al., 2005; a minimum of 9 cells/session in Bassett et al., 2018; a minimum of 6 cells/session in Peyrache et al., 2015). We used linear regression to assess the relationship between the number of identified HD cells and the accuracy of decoding (Figure 10). For 11 of the 12 computational methods, there was a significant negative correlation with *MAE* (absolute value of the *r* > 0.32, *p* < 0.05). The correlation between the number of HD cells and *MAE* for Vector Reconstruction failed to reach significance (*r* = −0.28, *p* = 0.08). It is possible the lack of significance for Vector Reconstruction is a consequence of generally poor decoding by this method. However, for all of the other decoding methods, the results suggest that as the number of classified HD cells increases, decoding accuracy improves (i.e., there is less error). The correlations (*r*-values) between *MAE* and head angular velocity were smaller than the correlations between *MAE* and the number of cells [*t*_(__22__)_ = 4.77, *p* < 0.001].

Given that the number of cells influences decoding accuracy, we next investigated whether the regional differences reported in the previous section can be explained by the number of cells per datasets. To address this question, we repeated our decoding analyses on datasets composed of a random subsample of at least 3 cells. For datasets with 6 or more cells, we split the datasets in half, each composed of 3 randomly selected cells (without repeats). Due to the higher computational demands of machine learning approaches, and the similarity in results between model-based and machine learning methods (see Figure 7), we only used model-based methods to investigate this question. In short, the results of this analysis again indicate superior decoding by ATN units relative to other regions, and similar decoding across parahippocampal and cortical cell populations (see Supplementary Material S15.1–S15.6). However, for some methods, MEC produced weaker decoding relative to other regions. For all methods, accuracy significantly varied across brain region [Supplementary Material S15.3; *F*_(__4,46__)_ > 2.57, *p* < 0.05]. For all model-based methods, ATN accuracy was greatest and furthest from the mean. For 3 of the methods, removing ATN resulted in a non-significant ANOVA [*F*_(__3_, _37__)_ < 2.86, *p* > 0.05]. For Generalized Linear Model, it was necessary to also remove the brain region that was second furthest from the mean, PoS [*F*_(__2_, _28__)_ < 3.34, *p* > 0.05], indicating that for this method, both PoS and ATN had significantly better decoding. Finally, for Vector Reconstruction and Optimal Linear Estimator, MEC was second furthest from the mean and removing MEC resulted in a non-significant ANOVA [*F*_(__2_, _27__)_ < 3.34, *p* > 0.05], indicating that for these methods ATN had significantly better decoding and MEC had significantly worse decoding. To summarize, subsampling the number of cells produced a similar outcome: while decoding is on average most accurate for ATN cell populations, accurate decoding is also possible for parahippocampal and cortical regions and is generally similar across PoS, PaS, MEC, and PC.

##### Tuning strength

**FIGURE 11:**
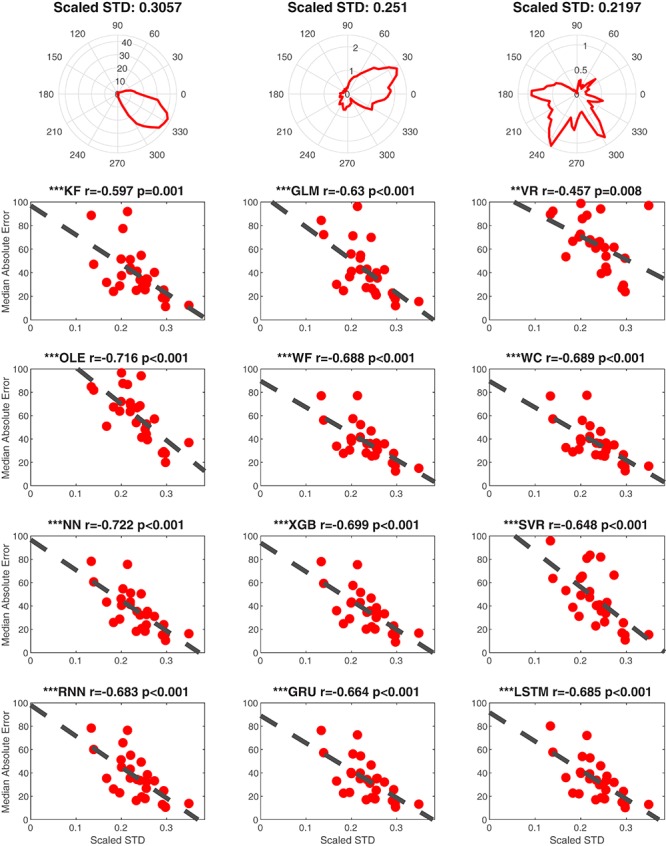
Tuning influences decoding accuracy. Top Row. Examples illustrating the relationship between scaled standard deviation (scaled STD) and tuning for single cells from ATN **(left)**, PoS **(middle),** and MEC **(right)**. The plots of tuning curves were smoothed by a Gaussian kernel function. The scaled STD is computed by taking standard deviation of the scaled (divided by maximum) firing rate. Bottom 4 Rows. Linear regression data is shown for each decoding method as a function of scaled STD (i.e., indicator of tuning strength). One cell was randomly selected from each dataset to avoid repeatedly sampling the same decoding score. ^∗∗∗^*p* < 0.001; ^∗∗^*p* < 0.01.

We additionally examined the contribution of the directional specificity of HD cell tuning to decoding accuracy. We first removed the influence of the cell’s firing rate by normalizing each cell’s tuning curve relative to the directional bin with the peak firing rate. We then calculated the standard deviation of the standardized firing rate by HD tuning function as a proxy for tuning strength (Figure 11 *Top*). Thus, a low standard deviation would reflect a flat tuning curve, and a high standard deviation would reflect a large peak in the preferred direction of the HD cell. Because this measure is independent of firing rate it is comparable to a measure of signal-to-noise. Finally, we performed linear regressions for each decoding method and a set of randomly selected cells from each dataset (Figure 11 *Bottom 4 rows*). This analysis indicated that each decoding method was significantly negatively correlated with *MAE* (absolute value of the *rs* > 0.4451, *ps* < 0.01). Thus, poorer tuning, independent of firing rate, is associated with lower decoding accuracy. Finally, the correlations (r-values) significantly varied across head angular velocity, tuning strength, and the number of cells [*F*_(__2_, _33__)_ = 36.83, *p* < 0.001]. Next head angular velocity was removed (furthest from the mean, see Statistical Methods) and tuning strength r-values were lower than the number of cells r-values [*F*_(__1_, _22__)_ = 10.74, *p* < 0.01]. Thus, the contribution of the number of HD cells had a larger impact than the tuning of individual cells which had a larger impact than the head angular velocity on decoding accuracy.

##### Firing rate

HD cell firing rates can vary between different HD cells (Taube, 2007) and on average the peak firing rate can differ between recording locations within cortical-limbic regions, approximately ranging from 2 spikes/s to 120 spikes/s (Blair and Sharp, 1995; Taube and Muller, 1998; Peyrache et al., 2015; Lozano et al., 2017). Notably, ATN HD cells on average express higher firing rates compared to those recorded in PoS (Blair and Sharp, 1995; Taube and Muller, 1998; Peyrache et al., 2015) and some preliminary work indicates that the firing rates of PaS and MEC HD cells are on average lower than ATN cells (Winter et al., 2015b). In addition, parahippocampal and cortical HD cells are often multi-dimensional or conjunctive for other spatial variables which can influence cell firing rates (Sargolini et al., 2006; Boccara et al., 2010; Wilber et al., 2014). Thus, because PoS HD cell populations are more sparsely active in a given recording session compared to ATN, it would be expected that decoding accuracy would be relatively low due to the limited spike information predicting the animals HD.

**FIGURE 12:**
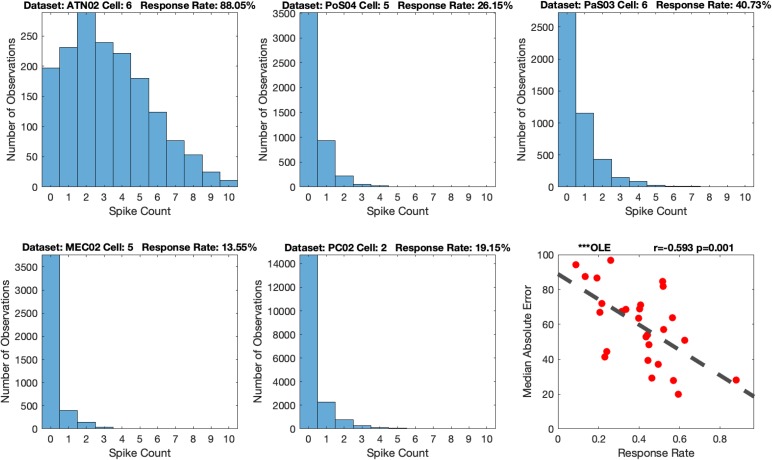
Example histograms of spike counts **(top three, bottom left and bottom middle)**, and an example Median Absolute Error (MAE) vs. response rate scatterplot **(bottom right)**: The dataset’s label and response rate are listed in the title. The example scatterplot illustrates the modest relationship between response rate and decoding accuracy. Scatterplots for all the 12 methods are shown in Supplementary Material S12. The dashed line in the scatterplot is the fitted linear regression. Optimal Linear Estimator (OLE).

Thus, to evaluate the relationship between decoding accuracy and firing rate, we created a measure that we refer to as the cell’s response rate, which is the proportion of video frames in which there was HD cell activity (i.e., cell spikes). As noted above, the number of cells per dataset can influence measures of *MAE*. We therefore subsampled one cell from each dataset, because some datasets had as few as 3 HD cells. We limited our analysis to the HD cell that expressed the greatest spike counts for each dataset. This allowed us to examine the response rate independent of the contribution of the number of cells. We next generated a histogram of the spike counts for the selected HD cell in each dataset and calculated the proportion of video frames in which spikes occurred (Figure 12). Thus, we hypothesized that a lower response rate, which is equivalent to a larger proportion of video frames with no spikes, should predict poorer decoding. The histograms suggest that, apart from ATN, parahippocampal and PC regions have very low response rates (less than half the ATN response rate). Importantly, as expected, *MAE* was negatively correlated with the response rate of the cells and was significant for every decoding method (absolute value of the *rs* > 0.3233, *ps* < 0.05; Figure 12 *Bottom Right and* Supplementary Material S12). Interestingly, the response rate seems to be the weakest contributor to decoding accuracy compared to number of cells and tuning. However, response rate was stronger than the absolute angular velocity [Omnibus ANOVA: *F*_(__3_, _44__)_ = 30.93, *p* < 0.001; Even after removing head angular velocity and tuning the *F*-test remained significant: *F*_(__1_, _22__)_ = 7.73, *p* ≤ 0.01]. Thus the strongest predictor of decoding accuracy was tuning strength which was significantly more predictive than the number of cells which was significantly more predictive than the response rate, which was significantly more predictive than the head angular velocity (i.e., Tuning > Number of Cells > Response Rate > Head Angular Velocity).

### Time Cost for Each Decoding Method

Time cost is an important indicator of the decoding method’s performance. Table 1 shows the mean elapsed time for training and testing for each decoding method.

**TABLE 1:** The average training time and testing time is shown for each decoding method grouped by category, model-based methods (above) and machine learning methods (below).

	**Method**	**Average training**	**Average testing**
		**time (seconds)**	**time (seconds)**
**Statistical**	VR	0.00	2.65
**model-based**	OLE	0.00	2.70
**methods**	KF	0.12	2.65
	WF	0.29	0.00
	GLM	2.88	2.63
	WC	31.58	0.00

**Machine**	SVR	126.88	0.46
**learning**	XGB	339.90	0.02
**methods**	FFNN	3213.17	1.94
	RNN	5274.18	2.23
	GRU	5548.07	4.15
	LSTM	6337.79	4.50

The full table can be seen in Supplementary Material S9.2. The testing time varies within a few seconds and is largely similar across the decoding methods. As for training, the time cost of using machine learning methods (mean: 3473.33 s or 57.88 min) was considerably greater than statistical model-based methods [mean: 34.87 s or 0.58 min; *t*_(__5__)_ = 3.13, *p* = 0.013].

## Discussion

The general aim of the present study was to compare statistical model-based and machine learning approaches for decoding an animal’s directional orientation from populations of HD cells. Overall, 12 computational models were evaluated using HD cell recordings from 27 datasets and from across 5 brain regions (PC, MEC, PaS, PoS, and ATN). Performance was similar for most methods (10 of the 12), but with significantly poorer performance by Vector Reconstruction and Optimal Linear Estimator methods. The generality of this result is supported by the fact that the findings were consistent across datasets from different laboratories (i.e., PC vs. other datasets), across different HD cell criteria (i.e., PC vs. other datasets), and across different behavioral testing procedures and recording environments (i.e., PC vs. ATN vs. all other datasets). For the Wiener Filter, Wiener Cascade and the machine learning methods, the prediction performance was highly accurate. One interesting result is that the Recurrent Neural Network model has a much simpler structure than the Gated Recurrent Unit model and Long Short-Term Memory model. In other words, the Recurrent Neural Network model has fewer parameters. The decoding result, however, indicates that these three methods do not have much performance difference. This result suggests that the more complex models may be overfitting the data, while the simpler, Recurrent Neural Network model may capture the critical parameters.

Both Kalman Filter and Generalized Linear Models are based on the hidden Markov chain framework. They make use of a Bayesian framework, assuming that firing rate is distributed according to HD. These two approaches model the activity of single cells as a function of HD. As a result, we can obtain the function curve generated by the model for spike count with HD as the input, which can be used as an estimation of the count-angle curve and the tuning curve. As shown in Figure 5, the Generalized Linear Model provides a more accurate model of the single cell tuning curves. Surprisingly, as shown in Figure 7, the more biologically accurate model of firing rate as a function of HD does not make the Generalized Linear Model more accurate than the Kalman Filter model. Instead, the latter has slightly lower error on average than the former. The decoding method in Generalized Linear Model, the point process filter, may account for this behavior. It uses Gaussian distribution for approximation, which greatly reduces the computation cost on the non-linear model prediction, but on the other hand may introduce more errors.

We found significantly poorer performance by Vector Reconstruction and Optimal Linear Estimator methods. There are several possible reasons for this inferior performance. For these methods, there are two critical criteria for the training dataset. First, the data should have a sufficiently strong unimodal peak for a specific HD between HD and firing rate, or else the estimation of preferred directions will be poor. This limitation may further explain poor decoding performance, particularly for cortical datasets, as classification of HD cells could include cells that are stable yet have low mean vector length. Second, the preferred direction vectors must cover the full range of directions from 0° to 360°. Without input data covering some HDs, some predicted HDs may never be achieved (see Figure 2). Both Vector Reconstruction and Optimal Linear Estimator methods are more sensitive than other approaches to violations of these criteria.

In general, the machine-learning methods displayed similar decoding accuracy to 4 of the model-based methods (Kalman Filter, Generalized Linear Models, Wiener Filter, Wiener Cascade). This indicates that the relationship between neural firing and HD is well captured by the 4 methods and do not differ from more complicated networks, which may have the problem of over-fitting the data. While it is possible that machine-learning methods would provide a benefit when dealing with larger scale recordings and high dimensional inputs, a large advantage of the model-based methods is their efficiency and robustness. All parameters can be efficiently estimated, and the linear methods can even have closed-form estimation. Related to these points, we also compared decoding accuracy with the elapsed time of training and testing decoding methods (time cost). All methods, with the exception of Vector Reconstruction and Optimal Linear Estimator, did not significantly differ with respect to *MAE*. However, the time cost was much greater for machine learning methods. This finding is not entirely surprising given the fact that machine learning methods include several parameters to be optimized and require Bayesian Optimization to tune the hyper-parameters. Thus, these features likely multiply the time cost of machine learning approaches. In sum, when considering the trade-off between accuracy and time, Kalman Filter, Generalized Linear Models, Wiener Filter and Wiener Cascade would be preferred methods for neural decoding of HD. Thus, for the datasets in the present study, machine-learning methods do not result in a better decoding and cost more with greater computation time.

We also contrasted the accuracy of HD cell decoding between 5 brain regions, including ATN, PoS, PaS, MEC, and PC. From these comparisons, we found that decoding performance varied considerably across datasets and brain regions (see Figure 9 and Supplementary Material S15.3). Specifically, decoding accuracy was greater for ATN when compared to parahippocampal cortex (PoS, MEC, PaS). Our initial analyses indicated that decoding accuracy was weakest for PC units. However, after controlling for the numbers of cells, our analyses indicated that decoding accuracy was similar across parahippocampal regions and PC, and for some decoding methods, was weaker for MEC populations. Our observations are consistent with a previous report suggesting greater decoding accuracy by ATN HD cell populations compared to PoS HD ensembles (see Supplementary Figure S1 in Viejo et al., 2018).

Greater decoding accuracy by ATN populations support the hypothesis that the ATN has a pivotal role in processing the HD cell signal (Cullen and Taube, 2017). Notably, damage to the ATN is known to disrupt HD signals in the parahippocampal cortex (Goodridge and Taube, 1997; Winter et al., 2015a); thus, a high precision readout of ATN HD signals may be critical for “downstream” networks (Wilber et al., 2014, 2015; Peyrache et al., 2015). HD cells in the ATN express some unique firing characteristics that may provide an advantage for neural decoding. For instance, previous studies have reported that HD cells in the ATN have higher peak firing rates compared to the PoS (Blair and Sharp, 1995; Taube and Muller, 1998; Peyrache et al., 2015) which, as described in the present study, has a significant impact on decoding accuracy. In addition, HD cells in the ATN can exhibit anticipatory firing characteristics, which can also influence the accuracy of HD decoding. Specifically, during a given head movement, ATN HD cells tend to fire maximally ∼25 ms before the animal’s head reaches the cell’s preferred firing direction (Blair and Sharp, 1995; Taube and Muller, 1998). A recent study by Zirkelbach et al. (2019) determined that anticipatory firing can improve decoding of the animals current HD by compensating for sensory or motion-induced decoding errors. In contrast to the ATN, experimental work has found that anticipatory firing is limited in the PoS (Taube and Muller, 1998). In PC, anticipatory firing has been reported by HD cells for action anticipation but not HD anticipation and the timescale of this anticipatory firing varies (Wilber et al., 2014). Anticipatory firing by HD cells in other regions of the parahippocampal cortex has not been well characterized (Winter et al., 2015b). Thus, it is possible that anticipatory firing by HD cells may have a critical influence over decoding accuracy across the entire HD cell circuit. Future studies should provide a quantitative comparison of these features of HD cell firing across thalamo-parahippocampal and cortical regions.

We considered several variables that may have contributed to the observed regional differences in decoding accuracy. These included the population firing rate (response rate), tuning strength, and cell density. Our analyses found that measures of tuning strength and cell density were significantly related to *MAE*. Notably, differences in the tuning strength of individual cells was the strongest predictor. The number of HD cells was the next strongest predictor and the overall response rate was comparatively the weakest predictor of decoding accuracy. However, all three methods were still significantly predictive of decoding accuracy. Thus, variance in *MAE* may be a consequence of differences in recording location, spike counts, tuning strength and HD cell density.

In summary, the present study suggests three general conclusions regarding the use of statistical model-based and machine learning approaches for neural decoding of HD: first, our comparison of different computational models suggests limitations in decoding accuracy by Vector Reconstruction and Optimal Linear Estimator methods. Second, we found that decoding accuracy is variable across the HD cell system, with superior decoding in ATN compared to parahippocampal and cortical regions. Last, we found that decoding accuracy can be influenced by variables such as tuning strength, the response rate, and the recording density of HD cells. Thus, the present study provides a framework for the use of these computational approaches for future investigation of the neural basis of spatial orientation.

## Data AvailabilIty Statement

The datasets generated for this study are available on request to the corresponding authors.

## Ethics Statement

The animal study was reviewed and approved by Dartmouth College IACUC and University of Lethbridge Animal Welfare Committee.

## Author Contributions

All authors contributed to the preparation of the manuscript.

## Conflict of Interest

The authors declare that the research was conducted in the absence of any commercial or financial relationships that could be construed as a potential conflict of interest.

## Footnotes

1 http://klustakwik.sourceforge.net

2 https://github.com/KordingLab/Neural_Decoding

3 https://github.com/fmfn/BayesianOptimization
