# Supplementary material for: A Comparison of Neural Decoding Methods and Population Coding Across Thalamo-Cortical Head Direction Cells
Source: Front Neural Circuits. 2019 Dec 10;13:75. doi: 10.3389/fncir.2019.00075 (PMC6914739; doi:10.3389/fncir.2019.00075)
Supplement: Supplementary file 1 [file Data_Sheet_1.pdf]

Supplementary Materials

S1. Kalman Filter model: parameter estimation (MLE) formulas.

$$\begin{cases} \hat{A}=(\sum_{k=2}^{M}\vec{X}_k\vec{X}_{k-1}^T)(\sum_{k=2}^{M}\vec{X}_{k-1}\vec{X}_{k-1}^T)^{-1} \\ \widehat{W}=\frac{1}{M-1}(\sum_{k=2}^{M}\vec{X}_k\vec{X}_k^T-\hat{A}\sum_{k=2}^{M}\vec{X}_{k-1}\vec{X}_k^T) \\ \hat{H}=(\sum_{k=1}^{M}\vec{Y}_k\vec{X}_k^T)(\sum_{k=1}^{M}\vec{X}_k\vec{X}_k^T)^{-1} \\ \hat{Q}=\frac{1}{M}(\sum_{k=1}^{M}\vec{Y}_k\vec{Y}_k^T-\hat{H}\sum_{k=1}^{M}\vec{X}_k\vec{Y}_k^T) \end{cases};$$

S2. Kalman Filter model: the algorithm for head angle prediction.

i. Initialize: $\widehat{\vec{X}}_0 = 0$ and $\boldsymbol{P_0} = 0$;

ii. Loop k from 1 to M:

At time k with all the parameters before time k obtained,

a) Time Update:

$$\begin{cases} \widehat{\vec{X}}_k^- = \hat{A}\widehat{\vec{X}}_{k-1} \\ P_k^- = \hat{A}P_{k-1}\hat{A}^T + \widehat{W} \end{cases};$$

b) Measurement Update:

$$\begin{cases} K_k = P_k^-\hat{H}^T(\hat{H}P_k^-\hat{H}^T + \hat{Q})^{-1} \\ \widehat{\vec{X}}_k = \widehat{\vec{X}}_k^- + K_k\left(\vec{Y}_k - \hat{H}\widehat{\vec{X}}_k^-\right) \\ P_k = (I - K_k\hat{H})P_k^- \end{cases};$$

iii. $\left\{\widehat{\vec{X}}_t\right\}_{t=1}^{M}$ is the predicted values for $\left\{\vec{X}_t\right\}_{t=1}^{M}$;

S3. Generalized Linear Model: the algorithm for estimation of $\boldsymbol{\mu_c}$ $\vec{\boldsymbol{a}}_c$ (Newton's method and MLE).

Let $\vec{\theta}_c^T = [\mu_c, \vec{a}_c^T]$ and $\vec{Z}_t^T = [1, \vec{X}_t^T]$, then:

Log-likelihood function: $LL = \sum_{t=1}^{M} Y_{t,c}\vec{\theta}_c^T\vec{Z}_t - e^{\vec{\theta}_c^T\vec{Z}_t} + const$;

1st and 2nd order derivative functions of the log-likelihood:

$$\begin{cases} \frac{\partial LL}{\partial\vec{\theta}_c} = \sum_{t=1}^{M}\left(Y_{t,c} - e^{\vec{\theta}_c^T\vec{Z}_t}\right)\vec{Z}_t \\ \frac{\partial^2 LL}{\partial\vec{\theta}_c\partial\vec{\theta}_c^T} = -\sum_{t=1}^{M} e^{\vec{\theta}_c^T\vec{Z}_t}\vec{Z}_t\vec{Z}_t^T \end{cases};$$

Recursive calculation of $\widehat{\vec{\theta}}_c$:

i. Initialize: $\left(\widehat{\vec{\theta}}_c\right)_0 = 0$;

ii. Loop until convergence:

$$\left(\widehat{\vec{\theta}}_c\right)_{i+1} = \left(\widehat{\vec{\theta}}_c\right)_i - \left(\frac{\partial^2 LL}{\partial\vec{\theta}_c\partial\vec{\theta}_c^T}\right)_i^{-1}\left(\frac{\partial LL}{\partial\vec{\theta}_c}\right)_i;$$

S4. Generalized Linear Model: the algorithm for Point Process Filter.

i. Initialize: $\vec{X}_{0|0} = 0$ and $W_{0|0} = 0$;

ii. Loop t from 1 to M:

At time t with all the parameters before time t obtained,

a) Time Update:

$$\begin{cases} \boldsymbol{W}_{t|t-1} = \widehat{\boldsymbol{A}}\boldsymbol{W}_{t-1|t-1}\widehat{\boldsymbol{A}}^T + \widehat{\boldsymbol{W}} \\ \vec{\boldsymbol{X}}_{t|t-1} = \widehat{\boldsymbol{A}}\vec{\boldsymbol{X}}_{t-1|t-1} \end{cases};$$

b) Measurement Update:

$$\begin{cases} W_{t|t}=\left(W_{t|t-1}^{-1}+\sum_{c=1}^{C} e^{\mu_c+\vec{a}_c^T\vec{X}_{t|t-1}}\vec{a}_c\vec{a}_c^T\right)^{-1} \\ \vec{X}_{t|t}=\vec{X}_{t|t-1}+W_{t|t}\sum_{c=1}^{C}\left(Y_{t,c}-e^{\mu_c+\vec{a}_c^T\vec{X}_{t|t-1}}\right)\vec{a}_c \end{cases};$$

iii. $\{\vec{\boldsymbol{X}}_{t|t}\}_{t=1}^{M}$ is the predicted values for $\{\vec{\boldsymbol{X}}_t\}_{t=1}^{M}$ of the testing set;

S5. Vector Reconstruction Method: the estimation of the preferred direction. Let $\boldsymbol{R_c}$ be the observed firing rate vector for cell $\boldsymbol{c}$, $\boldsymbol{H}$ be the observed head angle vector, $\boldsymbol{X} = [\mathbf{1}, \boldsymbol{cos(H)}, \boldsymbol{sin(H)}]$ to be the design matrix, then the model of cosine curve function fit is:

$$\boldsymbol{R_c = a * cos(H - \theta) + b}$$

$$\Rightarrow \boldsymbol{R_c = [a * cos(\theta)] * cos(H) - [a * sin(\theta)] * sin(H) + b}$$

$$\Rightarrow \boldsymbol{R_c = \beta_0 + \beta_1 * \ cos(H) + \beta_2 * sin(H) = X\vec{\beta}}$$

Where $\boldsymbol{\beta_0 = b}$; $\boldsymbol{\beta_1 = a * cos(\theta)}$; $\boldsymbol{\beta_2 = a * sin(\theta)}$; $\vec{\boldsymbol{\beta}} = [\boldsymbol{\beta_0, \beta_1, \beta_2}]^T$; $\boldsymbol{\theta}$ is the preferred direction angle. Note that in order to have one peak, the period should be 360º, so no multiplier for $\boldsymbol{H}$ is included in the model.

Therefore, the model is a linear regression problem on $\boldsymbol{R_c}$ and $\boldsymbol{X}$. The OLS solution is $\widehat{\vec{\boldsymbol{\beta}}} = (\boldsymbol{X^TX})^{-1}\boldsymbol{X^TR_c}$ and the fitted preferred direction vector is $\widehat{\vec{\boldsymbol{L}}}_c = \left[\frac{\hat{\beta}_1}{\sqrt{\hat{\beta}_1^2+\hat{\beta}_2^2}}, \frac{\hat{\beta}_2}{\sqrt{\hat{\beta}_1^2+\hat{\beta}_2^2}}\right]^T$.

For the function $\boldsymbol{angle}(\cdot)$, it is simply an arctan function applied on the two coordinates of the input vector, i.e. if vector $\vec{\boldsymbol{v}} = (\boldsymbol{v_x, v_y})$, then $\boldsymbol{angle}(\vec{\boldsymbol{v}}) = \mathbf{arctan}\left(\frac{\boldsymbol{v_y}}{\boldsymbol{v_x}}\right)$.

S6. Optimal Linear Estimator Method: the center of mass vector for the tuning curve function and the correlation matrix of firing rates for all cells.

- Center of mass vector: $\vec{\boldsymbol{L}}_c = \int \vec{\boldsymbol{V}} \boldsymbol{f_c}(\vec{\boldsymbol{V}}) d\vec{\boldsymbol{V}}$
- Correlation matrix: $\boldsymbol{Q_{ij}} = \int r_i r_j \boldsymbol{P}(\vec{\boldsymbol{r}} \mid \vec{\boldsymbol{V}}) d\vec{\boldsymbol{r}} d\vec{\boldsymbol{V}}$
- Model: $r_c | \vec{\boldsymbol{V}} \sim \boldsymbol{Normal}(\boldsymbol{f_c}(\vec{\boldsymbol{V}}), \sigma_c^2)$, r_i and r_j are independent

Where $\vec{\boldsymbol{V}}$ is the true head direction vector; $\vec{\boldsymbol{r}} = [r_1, r_2, \dots\ r_C]^T$ is the firing rate for each brain cells; $\boldsymbol{f_c}$ is the tuning function for cell $\boldsymbol{c}$.

Under the model, $\boldsymbol{Q_{ij}} = \sigma_i^2 * \boldsymbol{I}_{\{i=j\}} + \int \boldsymbol{f_i}(\vec{\boldsymbol{V}})\boldsymbol{f_j}(\vec{\boldsymbol{V}}) d\vec{\boldsymbol{V}}$ with $\boldsymbol{I}$ to be the indicator function.

S7. Wiener Filter, Wiener Cascade and all the machine learning methods: more details on the models and algorithms.

The training is to use the spike counts in the select bins to predict the trigonometric values (cosine and sine) of the head angle in the bin. Since all the time bins represent 0.2 second, using spike counts is the same as using firing rates with a constant multiplier, which will not affect the model on computing the head angles. In the paper, to compute the head angle, the spike counts used are from the bin of the head angle, together with 14 bins preceding to that bin, which corresponds to a 3 seconds period. In terms of formulas, suppose $\vec{\boldsymbol{X}}_t$ is the

centralized trigonometric head angle vector (centralized [cos, sin] vector) at time $\boldsymbol{t}$; $\vec{\boldsymbol{Y}}_{\boldsymbol{t}} = [\boldsymbol{Y}_{\boldsymbol{t},\boldsymbol{1}}, \boldsymbol{Y}_{\boldsymbol{t},\boldsymbol{2}}, \dots \boldsymbol{Y}_{\boldsymbol{t},\boldsymbol{C}}]^{\boldsymbol{T}}$ is the centralized spike counts vector for all observed brain cells at time $\boldsymbol{t}$ (i.e. $\boldsymbol{Y}_{\boldsymbol{t},\boldsymbol{c}}$ is the centralized spike counts for cell $\boldsymbol{c}$ at time $\boldsymbol{t}$), then the Wiener Filter, Wiener Cascade and all the machine learning methods will train a model that can predict $\vec{\boldsymbol{X}}_{\boldsymbol{t}}$ given $\vec{\boldsymbol{Y}}_{\boldsymbol{t}}, \vec{\boldsymbol{Y}}_{\boldsymbol{t-1}}, \dots \vec{\boldsymbol{Y}}_{\boldsymbol{t-14}}$.

For RNN, GRU and LSTM, the algorithms use $\vec{\boldsymbol{Y}}_{\boldsymbol{t}}, \vec{\boldsymbol{Y}}_{\boldsymbol{t-1}}, \dots \vec{\boldsymbol{Y}}_{\boldsymbol{t-14}}$ as the input to the component and compute the output $\vec{\boldsymbol{X}}_{\boldsymbol{t}}$. These three methods use the 15 input vectors separately and as a result, they are able to capture the time-dependencies in the data. For Wiener Filter, Wiener Cascade, feedforward neural network, SVR and XGBoost, the algorithms use the concatenated vector of $\vec{\boldsymbol{Y}}_{\boldsymbol{t}}, \vec{\boldsymbol{Y}}_{\boldsymbol{t-1}}, \dots \vec{\boldsymbol{Y}}_{\boldsymbol{t-14}}$, $[\boldsymbol{Y}_{\boldsymbol{t},\boldsymbol{1}}, \boldsymbol{Y}_{\boldsymbol{t},\boldsymbol{2}}, \dots \boldsymbol{Y}_{\boldsymbol{t},\boldsymbol{C}}, \boldsymbol{Y}_{\boldsymbol{t-1},\boldsymbol{1}}, \boldsymbol{Y}_{\boldsymbol{t-1},\boldsymbol{2}}, \dots \boldsymbol{Y}_{\boldsymbol{t-1},\boldsymbol{C}}, \dots \boldsymbol{Y}_{\boldsymbol{t-14},\boldsymbol{C}}]^{\boldsymbol{T}}$, to make prediction on $\vec{\boldsymbol{X}}_{\boldsymbol{t}}$. The settings for these five methods are the same as standard regression problems where the time-order information in the input is not considered.

S8. For Bayesian Optimization, the process is to set a searching range for each of the hyper-parameters in the model and then find the optimal value for each hyper-parameter based on a Gaussian process. The searching of the optimal values is an iterative procedure, where in each iteration, the model will be fitted once, i.e. the model parameters are optimized to get the lowest median absolute error (or mean absolute error, depend on which is used as the measurement). Therefore, it will cost a large amount of time and resources compared to assigning the values for hyper-parameters directly. In the paper, the searching ranges are set according to the understanding of the algorithm, behavior of the data and the available computer resources. The optimized hyper-parameter values are within the manually set range, not at the two boundaries, which suggests that our manually set ranges are reasonable. The hyper-parameters searched and their pre-determined searching ranges in each decoding method are listed below:

Decoding Method	Hyper-Parameter	Searching Range
WC	order of polynomial	2 to 11
SVR	number of iterations	200 to 501
	penalty parameter: C	1 to 4
XGB	maximum tree depth	5 to 16
	number of trees	50 to 101
	learning rate: eta	0.01 to 0.1
FFNN	layer 1 number of units	100 to 401
	layer 2 number of units	50 to 201
	layer 3 number of units	10 to 51
	dropout rate	0 to 0.5
	number of epochs	50 to 101
RNN	dimension of hidden layer	50 to 76
	dropout rate	0.1 to 0.3
	number of epochs	25 to 76
GRU	number of GRU units in hidden layer	20 to 31
	dropout rate	0.1 to 0.3
	number of epochs	20 to 31
LSTM	number of LSTM units in hidden layer	20 to 31
	dropout rate	0.1 to 0.3
	number of epochs	20 to 31

Median Absolute Error Table															
Datasets		# Cells	Method												Datasets Mean
Area	#		KF	GLM	VR	OLE	WF	WC	FFNN	XGB	SVR	RNN	GRU	LSTM	
PoS	01	6	27.27	26.68	39.32	41.39	25.44	25.96	20.74	22.16	35.59	19.51	18.84	18.75	26.80
	03	6	30.57	21.09	44.77	44.27	32.76	29.79	23.79	30.11	33.78	26.78	25.12	25.47	30.69
	02	5	28.87	36.61	70.35	63.84	30.51	31.10	28.70	28.99	31.13	22.94	23.27	22.08	34.87
	05	5	33.98	35.66	66.33	68.47	36.15	36.11	32.22	35.79	40.97	33.53	31.23	29.86	40.02
	04	5	37.50	41.15	98.75	96.67	38.20	37.75	40.51	43.33	49.15	44.94	39.44	39.66	50.59
PaS	06	9	25.58	23.46	61.39	48.37	25.95	25.28	18.36	20.17	26.37	18.00	17.74	17.79	27.37
	05	5	34.76	39.97	53.97	52.83	36.52	36.54	32.62	38.50	41.02	32.37	34.97	33.22	38.94
	02	7	41.25	43.00	65.22	66.80	36.04	36.49	35.26	34.64	40.39	34.42	34.39	33.75	41.80
	03	6	51.16	52.40	65.31	71.04	41.73	42.22	43.46	42.95	52.31	43.21	39.87	39.20	48.74
	04	5	54.69	69.94	94.09	94.09	46.90	46.52	50.34	46.74	81.88	49.31	46.72	46.09	60.61
ATN	03	6	11.32	12.04	23.88	19.93	12.52	12.73	10.48	9.12	10.87	10.60	10.76	10.25	12.87
	02	6	18.87	22.73	26.74	28.09	19.47	18.08	15.10	15.81	16.96	12.93	16.23	14.91	18.83
	04	6	17.96	17.85	52.32	27.70	17.03	16.05	15.91	14.52	15.06	11.04	13.60	12.43	19.29
	01	6	12.35	15.58	96.98	36.92	14.94	16.77	16.24	16.73	15.45	13.83	13.21	12.96	23.50
	05	5	25.36	20.29	29.52	29.09	27.69	26.57	23.99	22.90	25.54	24.72	25.69	24.17	25.46
MEC	03	9	25.02	27.37	60.86	54.02	28.06	26.49	18.24	20.14	22.94	16.35	17.05	17.03	27.80
	05	5	24.11	24.80	66.68	67.38	27.69	28.97	25.88	24.82	38.85	26.22	22.66	22.73	33.40
	04	6	44.25	42.73	66.99	63.49	41.31	40.52	41.41	42.81	47.38	35.62	35.04	34.96	44.71
	01	6	51.59	55.87	72.64	72.03	40.65	40.14	46.14	42.57	63.77	51.24	40.37	40.62	51.47
	02	6	77.46	71.27	85.79	87.52	57.37	55.93	54.65	57.55	65.51	65.80	56.23	54.05	65.76
PC	05	6	34.50	35.45	41.12	39.40	30.54	32.66	35.30	33.99	43.05	38.52	35.38	37.23	36.43
	07	3	31.80	30.08	53.54	50.94	33.84	32.61	43.34	35.95	53.21	35.37	33.01	36.01	39.14
	04	3	40.28	42.55	61.61	57.14	35.84	34.92	31.06	33.25	66.38	33.23	32.17	31.91	41.70
	01	3	42.14	54.83	67.94	68.96	52.32	51.27	51.03	51.57	83.45	55.14	54.52	52.86	57.17
	06	3	47.14	72.32	92.19	81.87	56.16	57.15	60.63	59.30	63.60	60.12	57.29	57.80	63.80
	02	3	91.88	96.24	88.73	86.69	77.09	77.41	75.61	75.35	80.82	76.50	72.57	72.05	80.91
	03	3	88.63	84.40	89.48	84.73	77.01	76.80	78.22	77.98	95.76	78.38	76.38	80.16	82.33
Methods Mean			38.90	41.35	64.31	59.40	37.03	36.77	35.90	36.21	45.97	35.95	34.21	34.00	

S9.1. *The data table of the **MAE** values for all the datasets with every method. The methods marked as dark blue are statistical methods and the methods marked as orange are machine learning methods. Within the data part, the cells highlighted by yellow are the smallest in the row and the cells highlighted by light red are the largest in the row (in "Datasets Mean" column, the marked cells are with respect to that column). Same as **Figure 5**, the abbreviations for the methods are: 'KF'=Kalman Filter, 'GLM'=General Linear Model, 'VR'=Vector Reconstruction, 'OLE'=Optimal Linear Estimator, 'WF'=Wiener Filter, 'WC'=Wiener Cascade, 'FFNN'=Feedforward Neural Network, 'XGB'=XGBoost, 'SVR'=Support Vector Regression, 'RNN'=Recurrent Neural Network, 'GRU'=Gated Recurrent Unit, 'LSTM'=Long Short-Term Memory.*

Training Time Cost (seconds)															
**Datasets**		**# Cells**	**Method**												**Datasets**
**Area**	**#**		**KF**	**GLM**	**VR**	**OLE**	**WF**	**WC**	**FFNN**	**XGB**	**SVR**	**RNN**	**GRU**	**LSTM**	**Mean**
**PoS**	**01**	6	0.28	2.23	0.02	0.05	0.59	22.14	1653.37	226.68	59.82	6844.09	7721.00	8411.04	2078.44
	**02**	5	0.11	2.08	0.01	0.01	0.03	50.08	1655.58	219.36	92.66	7311.72	7046.04	8145.76	2043.62
	**03**	6	0.09	2.33	0.00	0.00	0.07	22.92	1637.21	185.59	56.17	5904.34	6998.44	7933.86	1895.09
	**04**	5	0.10	1.87	0.00	0.00	0.07	16.71	1883.62	145.68	54.02	8252.32	7189.07	8246.62	2149.17
	**05**	5	0.09	1.60	0.02	0.01	0.07	17.21	1703.39	195.32	48.65	6144.99	2862.61	3147.19	1176.76
**PaS**	**02**	7	0.07	2.33	0.01	0.00	0.07	32.54	1484.02	185.69	89.86	7016.39	6435.97	2982.62	1519.13
	**03**	6	0.07	2.12	0.00	0.00	0.07	17.35	1562.27	202.76	71.53	6109.41	6706.81	3120.34	1482.73
	**04**	5	0.06	1.82	0.00	0.00	0.07	16.28	1529.37	124.50	53.51	8470.38	2559.95	6583.64	1611.63
	**05**	5	0.06	1.58	0.00	0.00	0.07	14.22	1686.50	176.31	47.98	2452.90	6702.53	7831.64	1576.15
	**06**	9	0.09	2.80	0.00	0.00	0.06	25.52	1623.37	282.32	65.92	5776.33	2617.98	3008.90	1116.94
**ATN**	**01**	6	0.03	0.24	0.00	0.00	0.14	39.48	1616.78	220.62	54.74	1947.33	1170.72	3989.91	753.33
	**02**	6	0.03	0.20	0.00	0.00	0.14	51.84	1454.38	183.67	86.14	933.30	1263.76	2950.63	577.01
	**03**	6	0.03	0.22	0.00	0.00	0.34	64.39	1322.14	228.36	177.97	953.28	1344.69	3143.05	602.87
	**04**	6	0.03	0.23	0.00	0.00	0.50	27.45	1559.96	229.80	72.79	2224.90	1096.08	3055.24	688.91
	**05**	5	0.02	0.16	0.00	0.00	0.37	26.86	1366.27	190.28	58.72	1864.19	2488.24	3594.30	799.12
**MEC**	**01**	6	0.06	1.96	0.00	0.00	0.34	28.23	3074.97	309.77	151.17	2149.45	5112.58	8761.71	1632.52
	**02**	6	0.06	1.89	0.00	0.00	0.30	50.11	2902.36	332.02	122.61	2060.36	6800.34	7933.49	1683.63
	**03**	9	0.09	3.08	0.01	0.00	0.44	28.37	3070.51	426.52	158.66	2309.48	7675.51	8249.33	1826.83
	**04**	6	0.06	1.99	0.00	0.00	0.42	32.79	2919.04	286.34	201.25	4988.33	6762.89	3260.77	1537.83
	**05**	5	0.06	1.75	0.00	0.00	0.32	39.82	3361.08	429.54	220.43	4251.21	9005.36	8190.85	2125.04
**PC**	**01**	3	0.19	5.88	0.00	0.00	0.78	18.34	4380.10	368.07	108.98	7001.04	6725.28	7888.68	2208.11
	**02**	3	0.34	11.44	0.00	0.00	0.36	14.62	5723.88	411.49	144.81	9675.37	9386.12	10635.40	3000.32
	**03**	3	0.24	4.47	0.00	0.00	0.68	21.62	4520.35	484.98	107.27	7075.30	6658.24	8127.01	2250.01
	**04**	3	0.19	5.44	0.00	0.00	0.66	37.64	3862.41	369.28	101.09	4813.49	6513.07	6951.66	1887.91
	**05**	6	0.20	9.10	0.01	0.00	0.28	77.79	9499.46	1177.01	529.30	5424.30	6022.78	7022.68	2480.24
	**06**	3	0.26	4.09	0.00	0.00	0.07	23.43	8396.41	771.30	232.89	13503.70	6707.88	7427.08	3088.93
	**07**	3	0.26	4.92	0.00	0.00	0.45	34.80	11306.73	813.89	256.87	6945.06	8223.91	10526.82	3176.14
**Methods Mean**			0.12	2.88	0.00	0.00	0.29	31.58	3213.17	339.90	126.88	5274.18	5548.07	6337.79	1739.57

Testing Time Cost (seconds)															
**Datasets**		**# Cells**	**Method**												**Datasets**
**Area**	**#**		**KF**	**GLM**	**VR**	**OLE**	**WF**	**WC**	**FFNN**	**XGB**	**SVR**	**RNN**	**GRU**	**LSTM**	**Mean**
**PoS**	**01**	6	3.76	2.64	2.96	2.20	0.00	0.00	1.29	0.01	0.21	2.25	4.81	5.88	2.17
	**02**	5	2.45	2.70	2.23	2.75	0.00	0.00	1.35	0.01	0.28	2.73	4.87	6.00	2.11
	**03**	6	2.33	2.32	2.20	2.40	0.00	0.00	1.18	0.01	0.31	2.46	6.35	5.82	2.12
	**04**	5	2.72	2.26	2.10	2.86	0.00	0.00	1.25	0.01	0.25	2.35	5.30	6.66	2.15
	**05**	5	2.84	2.25	2.49	2.35	0.00	0.00	1.18	0.01	0.23	2.28	2.45	2.74	1.57
**PaS**	**02**	7	2.33	2.23	2.28	2.48	0.00	0.00	1.18	0.01	0.53	2.59	4.86	2.68	1.76
	**03**	6	2.45	2.32	2.43	2.48	0.00	0.00	1.15	0.01	0.30	7.65	5.19	2.65	2.22
	**04**	5	2.23	2.28	2.20	2.42	0.00	0.00	1.41	0.01	0.25	6.74	2.14	5.32	2.08
	**05**	5	2.39	2.34	2.41	2.32	0.00	0.00	1.23	0.01	0.24	1.21	4.80	5.83	1.90
	**06**	9	2.30	2.46	2.26	2.33	0.00	0.00	1.11	0.02	0.34	2.33	2.19	2.66	1.50
**ATN**	**01**	6	2.20	2.44	2.32	2.37	0.00	0.00	2.22	0.01	0.09	2.08	2.12	5.50	1.78
	**02**	6	2.38	2.63	2.49	2.87	0.00	0.00	2.20	0.00	0.10	1.03	2.83	5.83	1.86
	**03**	6	2.27	2.56	2.44	2.33	0.00	0.00	2.62	0.01	0.12	1.03	2.82	5.11	1.78
	**04**	6	2.24	2.36	2.81	2.49	0.00	0.00	2.16	0.01	0.15	1.87	2.12	5.08	1.78
	**05**	5	2.39	2.54	2.55	2.68	0.00	0.00	2.45	0.00	0.07	2.39	4.25	6.17	2.12
**MEC**	**01**	6	2.33	2.64	2.81	2.64	0.00	0.00	2.15	0.01	0.45	1.11	4.42	5.82	2.03
	**02**	6	2.39	2.78	3.10	2.42	0.00	0.00	2.54	0.02	0.51	1.11	5.97	5.84	2.22
	**03**	9	2.91	2.78	2.91	2.95	0.01	0.00	2.33	0.03	0.77	1.15	4.74	5.74	2.19
	**04**	6	2.76	2.70	2.82	2.94	0.00	0.00	2.65	0.03	0.54	1.96	4.35	2.93	1.97
	**05**	5	2.65	2.72	2.70	2.89	0.01	0.00	3.15	0.01	0.51	2.31	17.62	6.14	3.39
**PC**	**01**	3	2.90	2.95	2.79	3.11	0.00	0.00	1.56	0.02	0.53	1.75	2.40	2.80	1.73
	**02**	3	3.19	3.03	2.93	3.41	0.01	0.00	1.86	0.03	0.75	1.48	2.55	3.22	1.87
	**03**	3	3.18	3.15	3.04	3.13	0.02	0.00	1.74	0.02	0.41	1.42	2.44	2.85	1.78
	**04**	3	2.95	2.82	2.91	3.18	0.01	0.00	1.59	0.02	0.44	1.52	2.68	3.13	1.77
	**05**	6	3.05	3.17	3.23	2.94	0.00	0.00	3.08	0.09	1.83	1.24	2.40	2.83	1.99
	**06**	3	2.90	3.02	3.12	3.08	0.00	0.00	2.64	0.03	1.17	2.92	2.71	3.13	2.06
	**07**	3	3.07	2.91	2.97	2.93	0.01	0.00	3.17	0.12	1.07	1.16	2.70	3.06	1.93
**Methods Mean**			2.65	2.63	2.65	2.70	0.00	0.00	1.94	0.02	0.46	2.23	4.15	4.50	1.99

S9.2. *The training time cost table and testing time cost table for all the datasets with every method. The methods marked as light blue are statistical methods and the methods marked as orange are machine learning methods. The cells are colored by: the smaller the value (time in second), the closer the color to light green; the larger the value, the closer the color to red.*

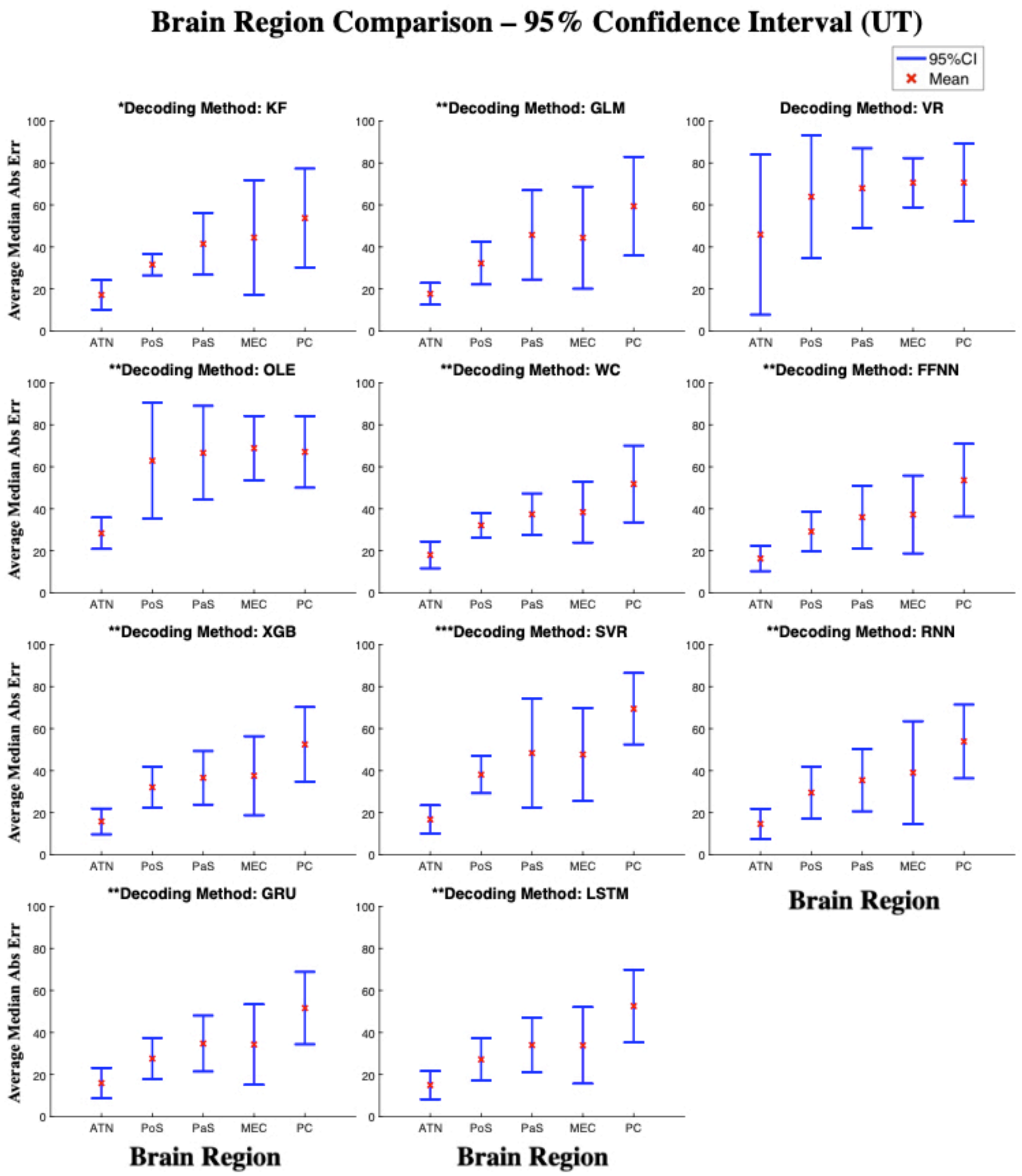

S10. *Median Absolute Error (MAE) values and error bars representing the 95% Confidence-intervals of the average MAE values for the 11 decoding methods not shown in Fig. 9. The significance levels are shown with symbols on the top-left corner (***: p-value < 0.001; **: p-value < 0.01; *: p-value < 0.05). Kalman Filter (KF), Generalized Linear Model (GLM), Vector Reconstruction (VR), Wiener Filter (WF), Wiener Cascade (WC), Support Vector Regression (SVR), XGBoost (XGB), Feedforward Neural Network (FFNN), Recurrent Neural Network (RNN), Gated Recurrent Unit (GRU), and Long Short-Term Memory (LSTM). Anterior Thalamic Nuclei (ATN), Postsubiculum (PoS), Parasubiculum (PaS), Medial Entorhinal Cortex (MEC), Parietal Cortex (PC).*

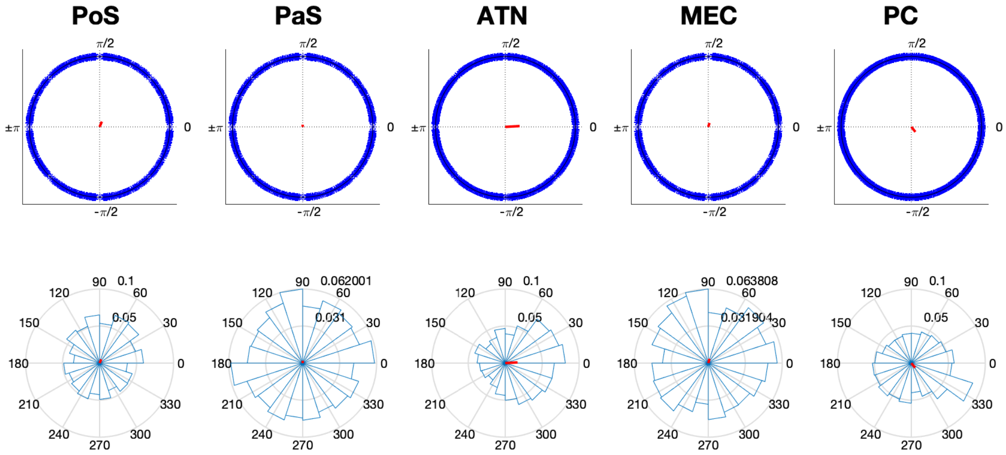

S11. *The time occupancy plots to check animal's trajectory biases. Top row: plot of all observed HDs on a unit circle. The red line is the mean resultant vector. Based on it, we can see that all the vectors are short in length, which indicates that the animal did not spend most of the time occupying a specific HD. Bottom row: circular histogram of the HDs. For each brain region, no direction has zero count, which suggests that the full range of HDs are well covered. Anterior Thalamic Nuclei (ATN), Postsubiculum (PoS), Parasubiculum (PaS), Medial Entorhinal Cortex (MEC), Parietal Cortex (PC).*

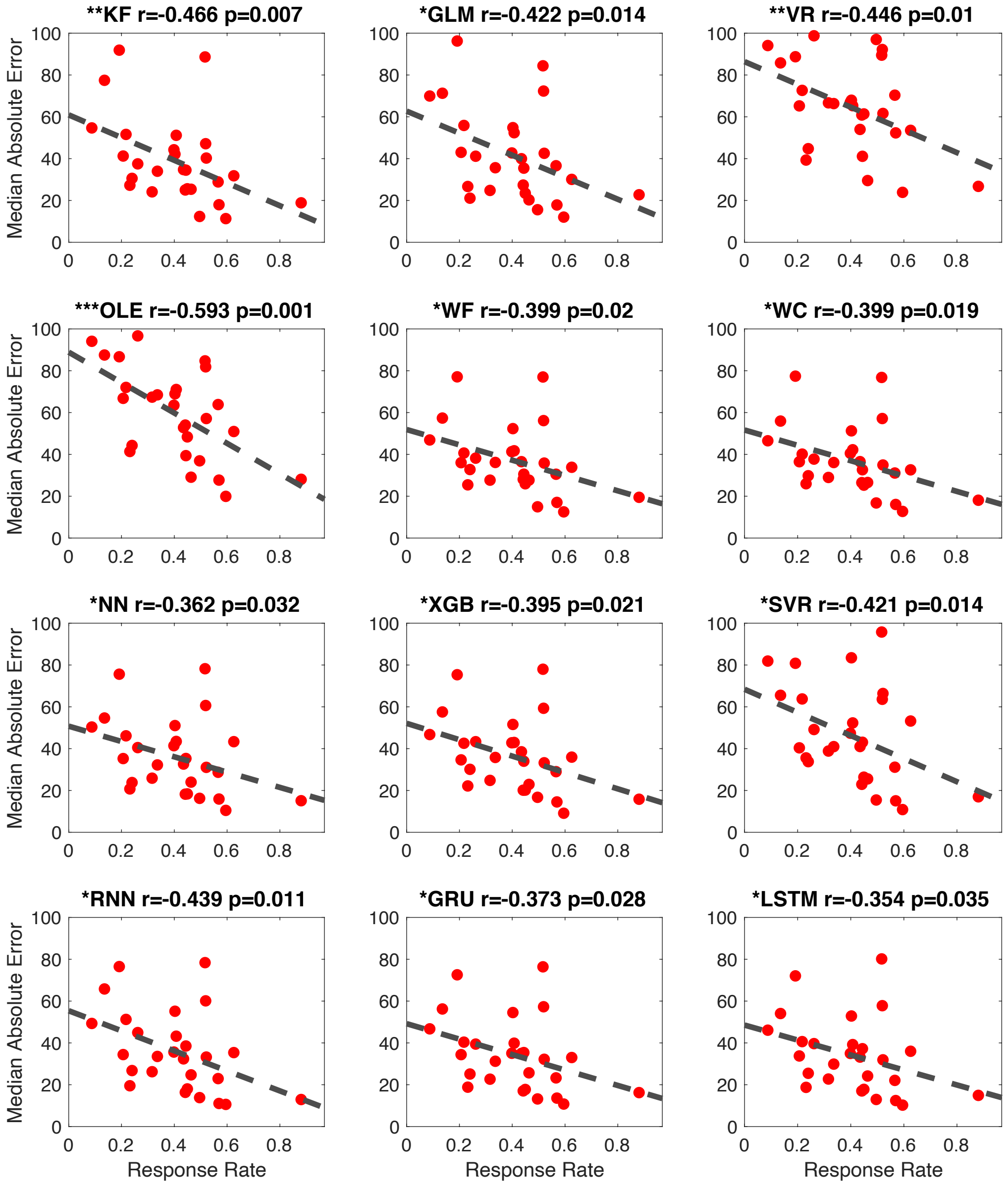

S12. *Scatterplots of median absolute error vs. response rate for all the 12 methods. The dashed line is the fitted linear regression. The correlation coefficient (r) and the corresponding p-value are shown on the top-right corner of each panel. The significance levels are shown with symbols on the top-left corner (***: p-value < 0.001; **: p-value < 0.01; *: p-value < 0.05). Kalman Filter (KF), Generalized Linear Model (GLM), Vector Reconstruction (VR), Wiener Filter (WF), Wiener Cascade (WC), Support Vector Regression (SVR), XGBoost (XGB), Feedforward Neural Network (FFNN), Recurrent Neural Network (RNN), Gated Recurrent Unit (GRU), and Long Short-Term Memory (LSTM).*

S13. The output for the lower 3/4 as training (LT) case in the cross-validation. Tables and charts are made following the same scheme as **S9** to **S12** and also **Fig. 5** to **Fig. 12** in the paper. Same as the UT case, the response rate seems to be the weakest contributor to decoding accuracy, while tuning seems to be the strongest, as there was significant variation in r-values across response rate, number of cells, and tuning (number of cells, tuning and response rate: $F_{(2, 33)}$=61.8, p<0.001; pairwise paired T-test: $|t_{(11)}|$>2.201, p<0.05)

Median Absolute Error Table

Datasets		# Cells	Method												Datasets
Area	#		KF	GLM	VR	OLE	WF	WC	FFNN	XGB	SVR	RNN	GRU	LSTM	Mean
PoS	03	6	25.93	26.59	37.21	35.00	24.57	24.16	24.15	23.48	38.13	22.77	22.51	21.22	**27.14**
	01	6	26.52	26.32	42.00	42.29	31.30	32.82	30.10	28.89	34.59	28.86	27.11	28.30	**31.59**
	02	5	33.71	35.54	72.90	65.23	37.73	36.74	32.40	34.06	37.77	31.13	31.82	29.67	**39.89**
	05	5	40.98	47.14	62.40	56.56	38.13	37.00	30.22	38.56	46.07	33.44	27.94	29.32	**40.65**
	04	5	43.58	49.89	72.50	71.91	50.80	51.62	52.34	47.41	60.20	50.92	47.51	50.63	**54.11**
PaS	06	9	30.53	31.66	60.29	40.26	30.85	31.36	22.20	23.26	25.78	22.77	23.27	23.02	**30.44**
	05	5	33.66	34.32	66.82	60.72	33.28	32.66	30.05	30.94	34.23	28.33	30.75	30.15	**37.16**
	03	6	50.04	48.93	67.79	69.43	41.61	40.47	38.98	41.06	45.19	42.83	39.50	39.08	**47.08**
	02	7	48.05	49.26	56.99	58.06	50.36	49.90	45.35	50.49	49.25	49.05	46.04	46.63	**49.95**
	04	5	58.55	63.09	68.20	70.01	66.06	66.18	65.33	65.62	94.09	64.32	64.21	62.51	**67.35**
ATN	03	6	13.69	15.88	25.93	20.30	13.11	13.02	11.85	11.42	14.31	10.73	10.07	11.07	**14.28**
	01	6	13.69	19.91	35.26	23.79	18.17	14.97	15.50	14.30	14.24	11.33	14.20	14.98	**17.53**
	02	6	19.22	21.32	29.02	28.73	18.90	20.12	18.10	16.84	17.94	16.15	16.94	16.37	**19.97**
	05	5	18.96	23.01	35.33	30.23	21.24	21.66	20.69	18.47	21.48	17.63	18.85	17.74	**22.11**
	04	6	21.83	31.58	50.87	31.09	22.94	22.72	15.11	16.21	20.21	14.60	14.68	13.86	**22.97**
MEC	03	9	24.15	24.06	42.90	36.75	21.89	22.35	17.01	19.00	26.05	15.95	15.69	15.78	**23.46**
	05	5	33.69	40.41	58.96	58.40	37.04	36.18	32.62	35.65	47.09	33.36	34.73	34.96	**40.26**
	04	6	31.19	31.73	60.16	56.24	40.51	40.40	40.45	39.30	47.67	36.17	35.45	35.59	**41.24**
	01	6	40.72	45.28	52.72	51.26	41.54	41.37	39.95	42.80	57.42	42.33	40.31	38.86	**44.55**
	02	6	58.65	58.62	63.07	63.15	52.93	55.21	60.43	52.30	74.81	55.85	52.73	53.73	**58.46**
PC	05	6	36.54	36.68	52.68	51.08	36.88	36.80	28.44	30.99	54.82	33.63	29.22	30.69	**38.20**
	04	3	44.01	47.19	63.07	61.19	43.59	42.38	38.91	41.42	72.08	41.57	39.86	39.40	**47.89**
	07	3	44.51	37.76	53.22	58.74	56.04	55.59	45.86	59.78	50.79	51.92	49.89	44.54	**50.72**
	06	3	48.24	45.49	78.43	64.89	50.67	50.13	51.61	50.85	59.96	52.37	49.01	48.08	**54.14**
	01	3	53.33	43.36	84.65	86.67	47.08	46.40	51.83	58.05	94.13	39.83	51.73	52.55	**59.13**
	02	3	86.12	86.79	89.10	87.00	80.17	79.77	75.84	79.14	94.37	79.52	76.59	75.15	**82.46**
	03	3	64.86	63.98	73.14	73.82	91.71	89.22	90.01	90.78	#####	91.85	88.82	93.38	**84.91**
**Methods Mean**			**38.70**	**40.22**	**57.61**	**53.81**	**40.71**	**40.41**	**37.97**	**39.30**	**49.63**	**37.75**	**37.02**	**36.94**	

S13.1. *The data table of the* ***MAE*** *values for all the datasets with every method. The methods marked as dark blue are statistical methods and the methods marked as orange are machine learning methods.*

Training Time Cost (seconds)															
Dataset		# Cells	Method												Datasets Mean
Area	#		KF	GLM	VR	OLE	WF	WC	FFNN	XGB	SVR	RNN	GRU	LSTM	
PoS	01	6	0.15	1.76	0.01	0.03	0.15	53.25	3879.26	516.30	406.13	6195.49	2812.56	8344.15	1850.77
	02	5	0.06	1.73	0.00	0.00	0.15	60.34	3794.30	403.35	274.32	2449.24	2551.08	6706.25	1353.40
	03	6	0.05	2.03	0.00	0.00	0.21	79.08	4220.17	460.11	194.89	5609.68	8026.83	3939.46	1877.71
	04	5	0.04	1.69	0.00	0.00	0.21	74.47	4053.98	326.30	235.53	2682.21	6902.43	9373.14	1970.83
	05	5	0.06	1.34	0.00	0.01	0.08	89.01	3826.20	316.47	153.33	2217.44	9507.07	2988.38	1591.62
PaS	02	7	0.04	1.82	0.00	0.00	0.08	37.16	3975.46	518.87	174.58	2509.92	8245.67	2975.75	1536.61
	03	6	0.04	1.88	0.00	0.00	0.09	49.81	4061.37	441.73	230.80	4317.33	2627.22	3518.89	1270.76
	04	5	0.05	1.51	0.00	0.00	0.09	64.38	4119.29	354.04	163.61	4159.22	2580.39	3128.42	1214.25
	05	5	0.05	1.39	0.00	0.00	0.12	83.45	3570.23	331.26	167.81	4012.77	2614.32	3112.62	1157.84
	06	9	0.08	2.39	0.00	0.00	0.09	105.50	4062.68	511.40	151.19	4339.69	5167.84	2962.04	1441.91
ATN	01	6	0.01	0.15	0.00	0.00	0.15	38.92	1571.45	224.01	157.58	1917.10	2550.94	3153.98	801.19
	02	6	0.01	0.14	0.00	0.00	0.15	33.45	1664.85	205.92	84.85	1954.71	2397.91	3927.18	855.76
	03	6	0.01	0.15	0.00	0.00	0.25	82.11	1717.14	195.78	75.80	1007.91	1378.93	3967.26	702.11
	04	6	0.01	0.15	0.00	0.00	0.22	31.26	1949.81	286.03	74.85	972.43	3095.10	3090.20	791.67
	05	5	0.01	0.12	0.00	0.00	0.21	24.53	1622.24	178.78	71.37	2209.06	2426.32	3755.84	857.37
MEC	01	6	0.03	1.70	0.00	0.00	0.11	41.84	3511.80	338.48	132.31	5194.87	5269.19	6415.07	1742.12
	02	6	0.03	1.67	0.00	0.00	0.10	41.19	3793.20	389.86	147.52	4834.42	8150.63	7785.08	2095.31
	03	9	0.04	2.67	0.00	0.00	0.05	59.33	3834.24	664.43	173.80	5642.42	6744.89	6389.21	1959.26
	04	6	0.04	1.77	0.00	0.00	0.03	54.42	3748.06	361.80	131.15	2311.59	10195.11	9097.60	2158.47
	05	5	0.03	1.39	0.00	0.00	0.03	78.90	3558.30	414.83	361.67	5695.06	6146.01	8106.56	2030.23
PC	01	3	0.10	5.11	0.00	0.00	0.05	34.30	9053.04	753.63	227.95	8227.18	7415.72	13029.98	3228.92
	02	3	0.13	10.45	0.00	0.00	0.66	22.94	5737.22	467.68	146.62	7179.07	10452.85	12866.82	3073.70
	03	3	0.10	3.88	0.00	0.00	0.10	56.08	9144.48	783.18	226.77	6120.27	6720.67	7667.50	2560.25
	04	3	0.08	4.80	0.00	0.00	0.04	50.00	8659.60	629.74	258.64	13157.57	5704.71	7793.81	3021.58
	05	6	0.09	8.19	0.00	0.00	0.07	81.08	8508.58	1035.25	320.95	5591.91	6048.60	7318.64	2409.45
	06	3	0.08	3.80	0.00	0.00	0.19	40.77	9327.87	767.19	218.27	13029.69	6312.61	7974.13	3139.55
	07	3	0.12	4.43	0.00	0.00	0.14	64.90	12229.88	875.94	289.34	6058.18	6996.23	7998.97	2876.51
Methods Mean			0.06	2.52	0.00	0.00	0.14	56.76	4784.99	472.31	194.51	4799.87	5520.07	6199.52	1835.90

Testing Time Cost (seconds)															
Dataset		# Cells	Method												Datasets Mean
Area	#		KF	GLM	VR	OLE	WF	WC	FFNN	XGB	SVR	RNN	GRU	LSTM	
PoS	01	6	2.74	2.30	1.86	1.72	0.00	0.00	2.76	0.02	0.72	2.65	2.17	5.72	1.89
	02	5	2.03	1.71	1.67	1.67	0.00	0.00	2.70	0.02	0.63	1.03	2.28	5.64	1.61
	03	6	1.97	1.67	1.66	1.68	0.00	0.00	2.53	0.04	0.38	2.68	4.52	2.90	1.67
	04	5	1.66	2.19	1.86	1.74	0.00	0.00	3.69	0.01	0.60	1.49	4.00	5.84	1.92
	05	5	2.49	1.74	1.65	1.63	0.00	0.00	2.55	0.03	0.40	1.12	4.22	2.48	1.52
PaS	02	7	1.79	1.63	1.65	1.71	0.00	0.00	2.45	0.04	0.76	1.09	4.13	2.56	1.49
	03	6	1.74	2.16	2.02	1.99	0.00	0.00	3.12	0.03	0.77	2.41	2.20	3.29	1.64
	04	5	2.01	2.02	2.00	2.07	0.00	0.00	3.13	0.01	0.72	2.24	2.13	2.79	1.59
	05	5	2.00	1.75	1.71	1.73	0.00	0.00	2.91	0.01	0.63	2.30	2.16	3.18	1.53
	06	9	2.07	1.67	1.72	1.71	0.00	0.00	2.50	0.05	0.67	2.54	4.28	2.73	1.66
ATN	01	6	1.68	1.84	1.71	1.68	0.00	0.00	2.47	0.01	0.12	2.03	4.24	5.53	1.78
	02	6	1.72	1.88	1.73	1.76	0.00	0.00	2.46	0.01	0.10	2.26	5.01	4.62	1.80
	03	6	1.69	1.84	1.76	1.70	0.00	0.00	2.61	0.01	0.12	1.03	2.34	6.54	1.64
	04	6	1.68	1.69	1.71	1.70	0.00	0.00	2.41	0.01	0.12	1.07	4.68	5.96	1.75
	05	5	1.75	1.81	2.01	1.81	0.00	0.00	2.36	0.01	0.12	2.33	4.94	4.88	1.84
MEC	01	6	1.83	1.86	1.96	1.79	0.00	0.00	2.56	0.01	0.69	1.89	4.39	5.61	1.89
	02	6	2.10	1.94	2.09	1.77	0.00	0.00	2.66	0.02	0.65	2.83	4.32	4.46	1.90
	03	9	1.79	1.81	2.04	1.85	0.00	0.00	3.25	0.04	0.54	2.49	4.92	6.14	2.07
	04	6	1.84	1.89	1.83	1.86	0.00	0.00	2.94	0.01	0.68	1.13	19.43	5.56	3.10
	05	5	1.82	1.87	1.84	2.21	0.00	0.00	2.95	0.03	0.55	2.58	5.36	6.81	2.17
PC	01	3	1.96	2.11	2.33	2.56	0.00	0.00	2.94	0.06	1.13	1.69	2.57	3.65	1.75
	02	3	2.03	2.07	2.51	2.37	0.00	0.00	1.72	0.03	0.71	1.61	3.26	3.16	1.62
	03	3	1.96	2.27	1.95	2.13	0.00	0.00	2.86	0.09	1.05	1.25	2.46	2.83	1.57
	04	3	1.97	2.36	2.34	1.99	0.00	0.00	3.70	0.03	1.12	3.63	2.38	3.07	1.88
	05	6	2.37	1.99	2.00	2.00	0.00	0.00	3.15	0.03	1.42	1.42	2.28	3.03	1.64
	06	3	2.08	1.97	1.96	1.97	0.00	0.00	3.18	0.14	0.58	3.07	2.32	3.29	1.71
	07	3	2.04	2.07	2.02	2.07	0.00	0.00	3.03	0.04	1.29	1.30	2.36	2.93	1.60
Methods Mean			1.96	1.93	1.91	1.88	0.00	0.00	2.80	0.03	0.64	1.97	4.05	4.27	1.79

S13.2. *The training time cost table and testing time cost table for all the datasets with every method. The methods marked as light blue are statistical methods and the methods marked as orange are machine learning methods. The cells are colored by: the smaller the value (time in second), the closer the color to light green; the larger the value, the closer the color to red.*

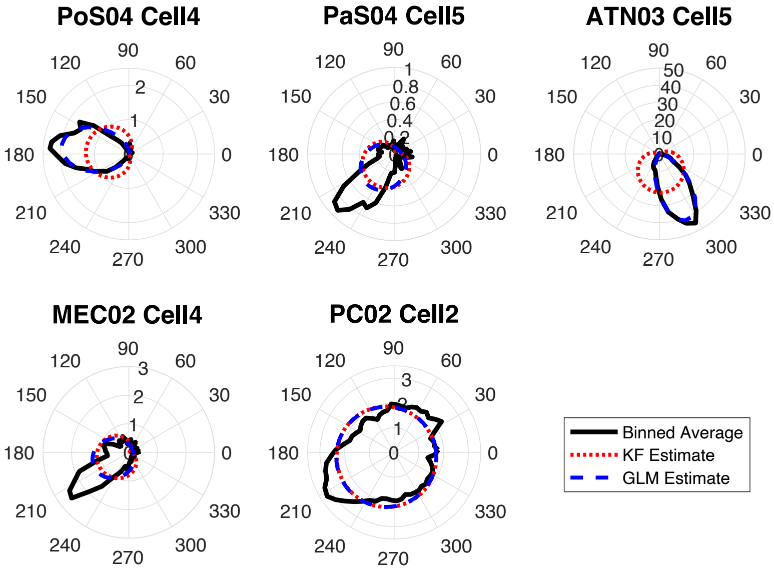

S13.3. *The true-vs-estimated tuning plots in 6-degree bins for one typical dataset in each brain region (Fig.5 LT version): The polar plots show firing rates vs. HD. The black curves are the true tuning functions, smoothed by a Gaussian kernel function. The red curves are the estimated functions using the Kalman Filter method and the blue curves are the estimated functions using the Generalized Linear Model method. Anterior Thalamic Nuclei (ATN), Postsubiculum (PoS), Parasubiculum (PaS), Medial Entorhinal Cortex (MEC), Parietal Cortex (PC).*

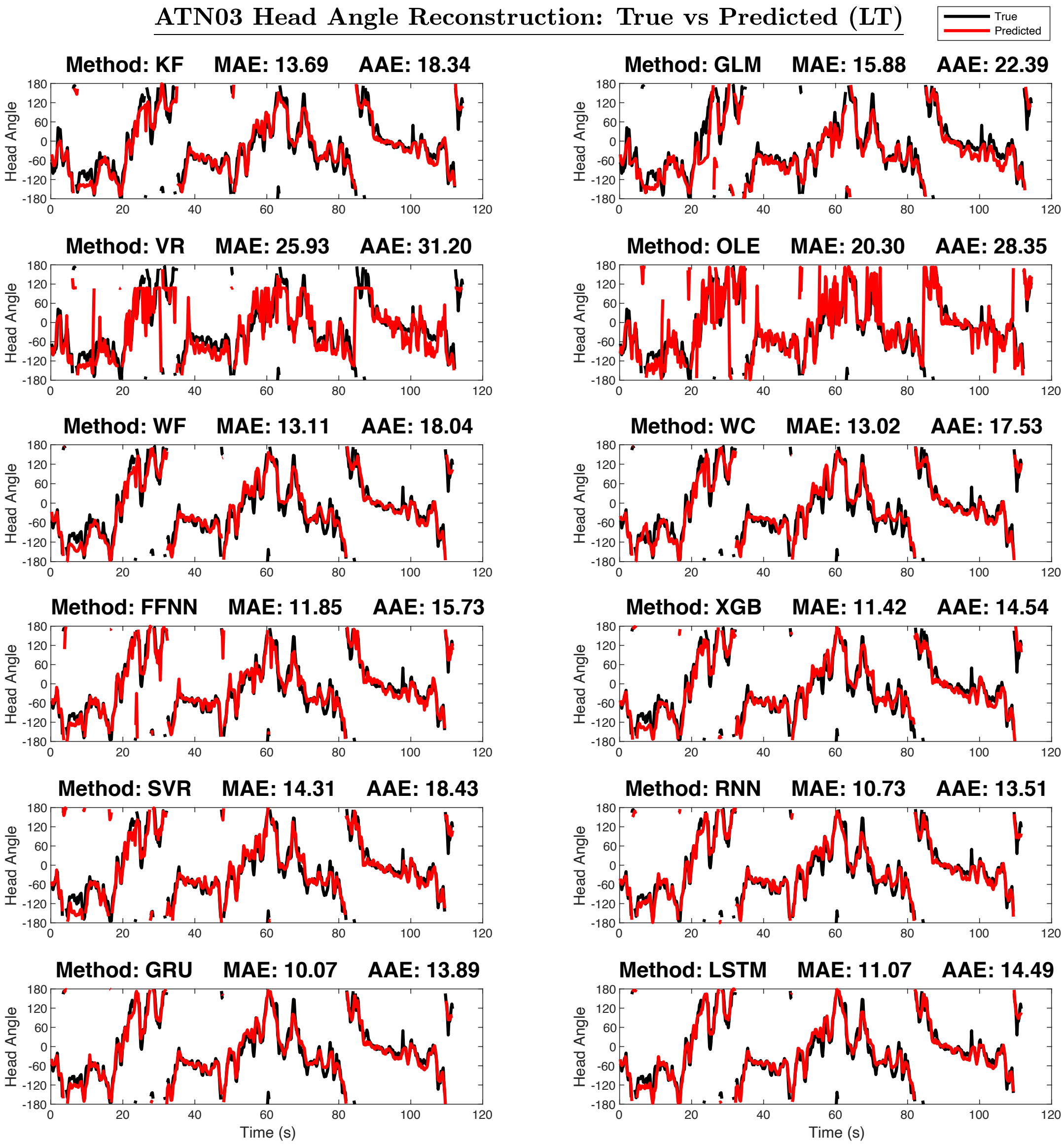

S13.4. *The true-vs-predicted head angle plotted as a function of time for a representative ATN dataset for each of the 12 decoding methods (Fig.6 LT version): The black curves are the true curves and the red curves are the predicted curves. Test data is shown. Predicted curves are constructed using a model generated from a separate training segment of the data. The method name and decoding accuracy measured as median absolute error (MAE) are shown on the title of each plot (average absolute error, AAE, is also shown).*

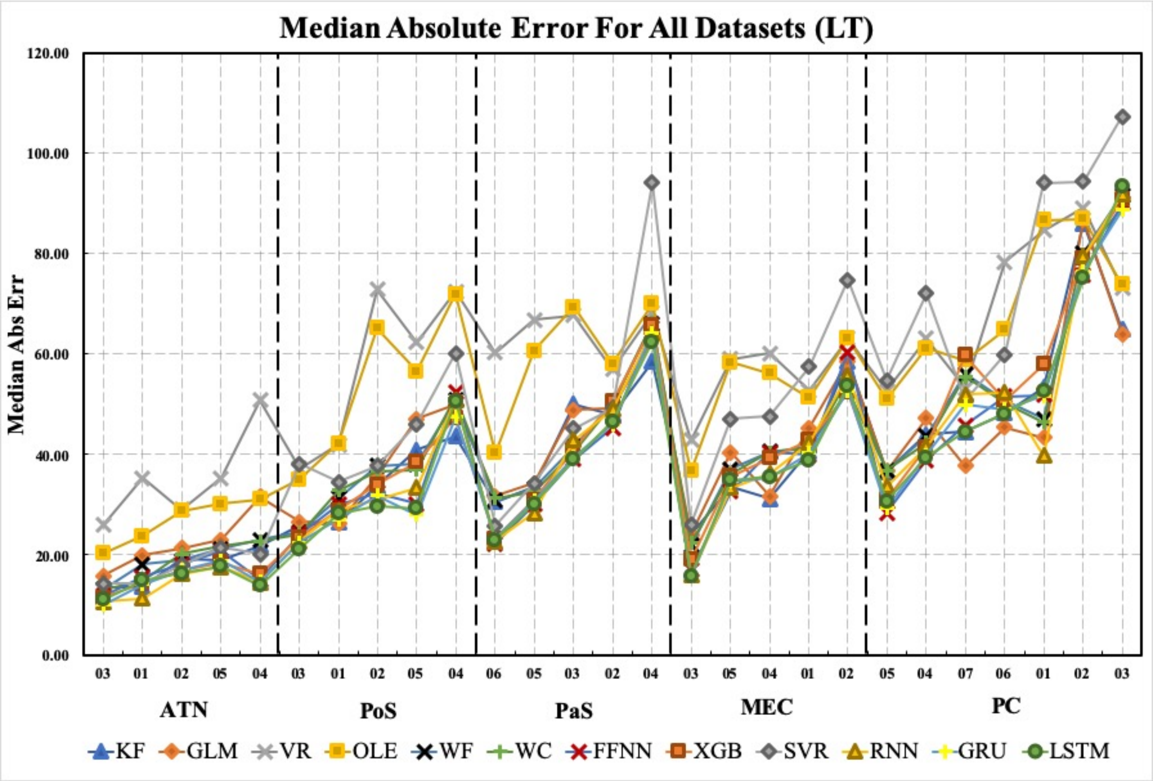

S13.5. *(Fig.7 LT version) The median absolute error is shown for each dataset and each decoding method. ATN, PoS, PaS and MEC each have 5 datasets while PC has 7 datasets. Datasets for each brain region are sorted from lowest to highest median absolute error (i.e., from best to worst decoding accuracy).*

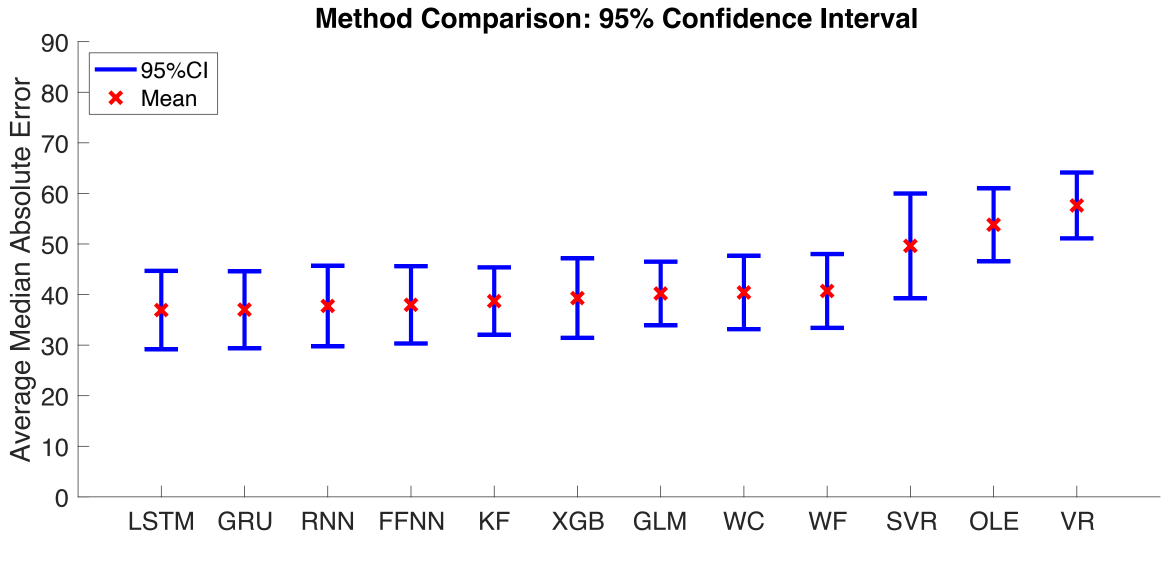

S13.6. *(Fig.8 LT version) Mean±95% Confidence-Interval (CI) Median Absolute Error (MAE) for each decoding method. Data from different brain regions and datasets were pooled. The ANOVA output: all 12 methods,* $F_{(11, 312)}$*=3.65, p<0.001; without VR and OLE,* $F_{(9, 260)}$*=0.96, p=0.47.*

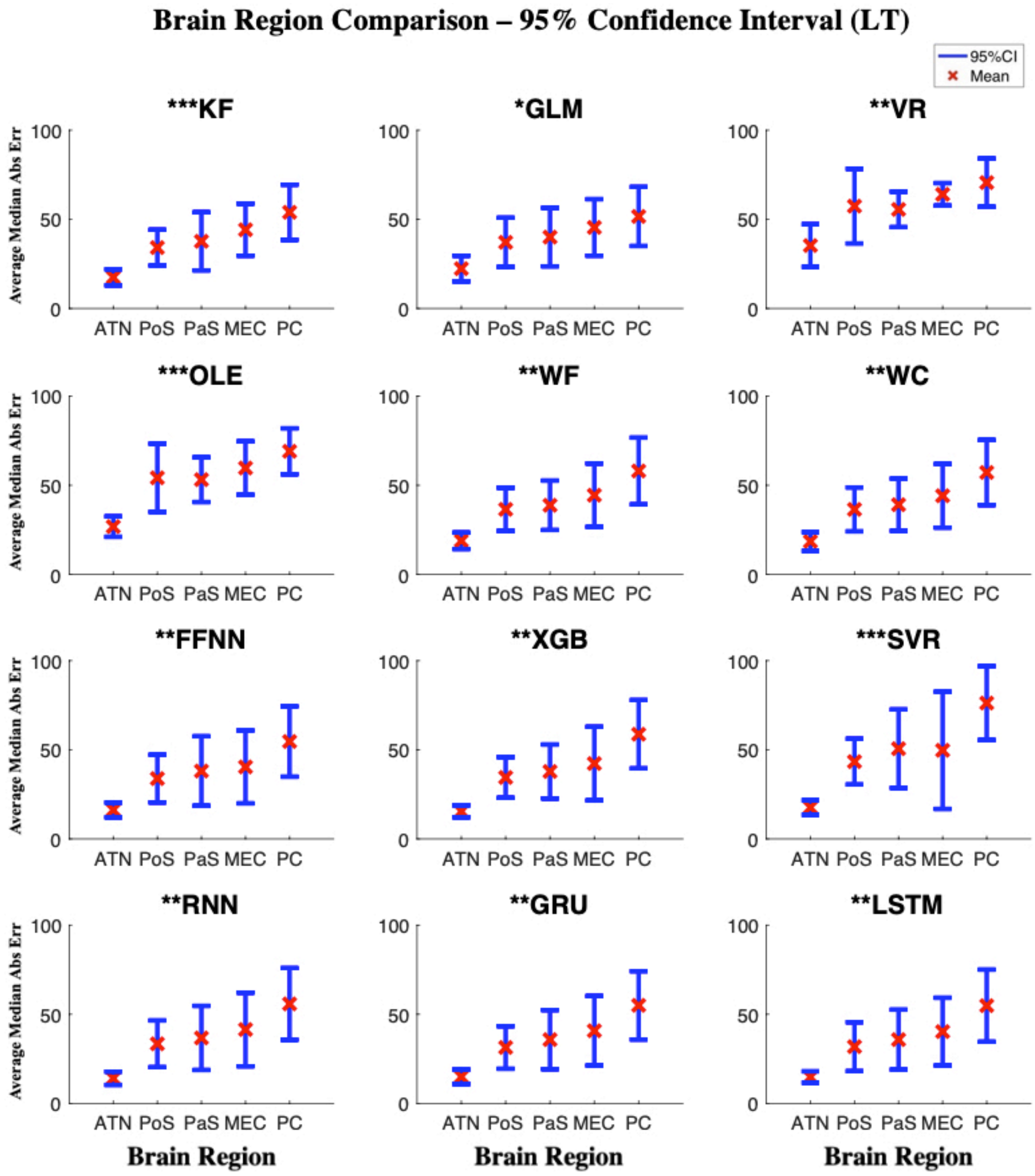

S13.7. *Mean Median Absolute Error (MAE) values and error bars representing the 95% Confidence-intervals of the average MAE values for the 12 decoding methods. For all the 12 methods, accuracy significantly varied across brain region ($F_{(4, 22)}>2.82$, $p<0.05$). For 11 of the methods, removing ATN resulted in a non-significant ANOVA ($F_{(3, 18)}<3.16$, $p>0.05$). The only exception was Support Vector for which it was necessary to also remove the brain region that was the second furthest from the mean, PC ($F_{(2,12)}<3.89$, $p>0.05$). The significance levels are shown with symbols on the top-left corner (***: p-value < 0.001; **: p-value < 0.01; *: p-value < 0.05). Kalman Filter (KF), Generalized Linear Model (GLM), Vector Reconstruction (VR), Wiener Filter (WF), Wiener Cascade (WC), Support Vector Regression (SVR), XGBoost (XGB), Feedforward Neural Network (FFNN), Recurrent Neural Network (RNN), Gated Recurrent Unit (GRU), and Long Short-Term Memory (LSTM).*

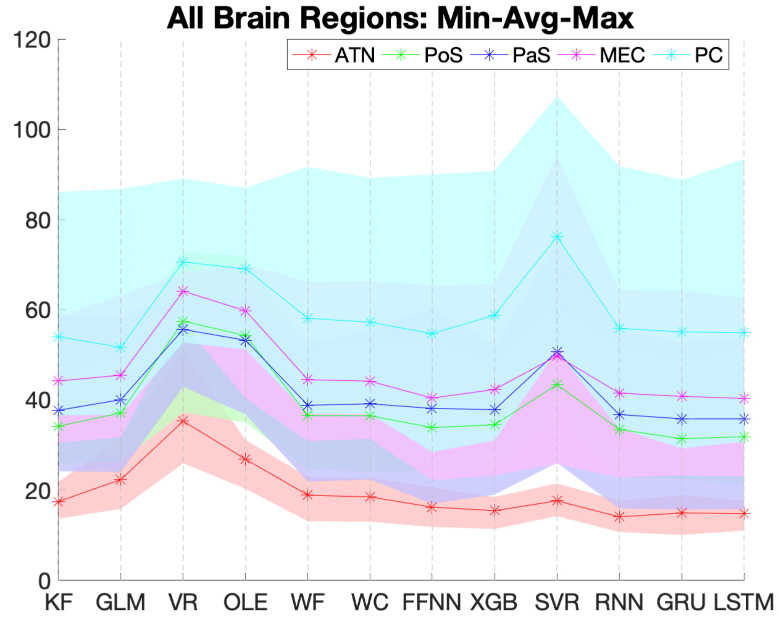

S13.8. *Decoding accuracy varies across brain regions (Fig.9 LT version).*

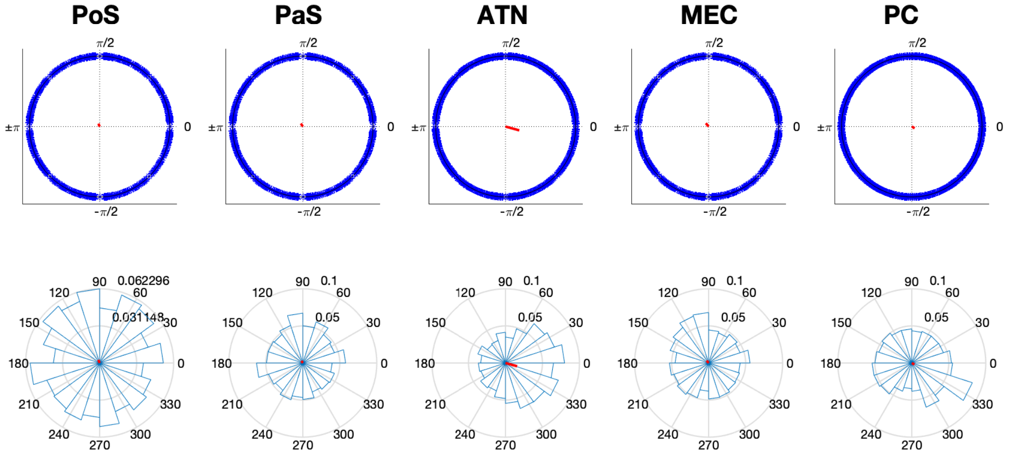

S13.9. *The time occupancy plots to check animal's trajectory biases, LT case. The observed features are similar to the UT case: in the upper row, the mean resultant vectors are all short in length, which indicates that the animal did not spend most of the time around a specific direction; in the bottom row, no direction has zero count, which suggests that the full range of HDs are well covered. Anterior Thalamic Nuclei (ATN), Postsubiculum (PoS), Parasubiculum (PaS), Medial Entorhinal Cortex (MEC), Parietal Cortex (PC).*

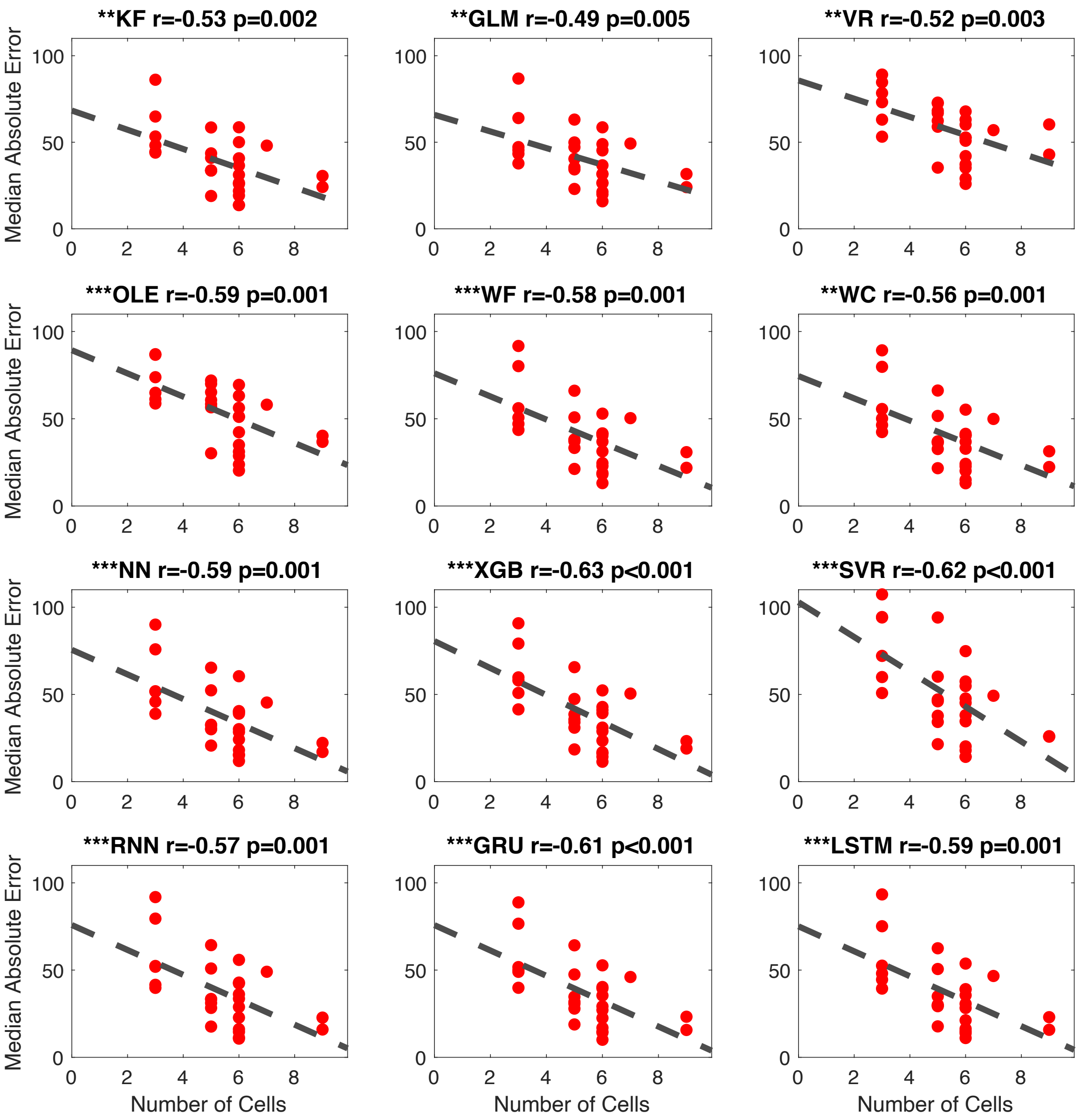

S13.10. *(Fig.10 LT version) Scatterplots of median absolute error vs. number of cells for all 12 methods. The dashed line is the fitted linear regression. The correlation coefficient (r) and the corresponding p-value are shown on the top-right corner of each panel. The significance levels are shown with symbols on the top-left corner (***: p-value < 0.001; **: p-value < 0.01; *: p-value < 0.05). All correlations are significant (p<0.05). Kalman Filter (KF), Generalized Linear Model (GLM), Vector Reconstruction (VR), Wiener Filter (WF), Wiener Cascade (WC), Support Vector Regression (SVR), XGBoost (XGB), Feedforward Neural Network (FFNN), Recurrent Neural Network (RNN), Gated Recurrent Unit (GRU), and Long Short-Term Memory (LSTM).*

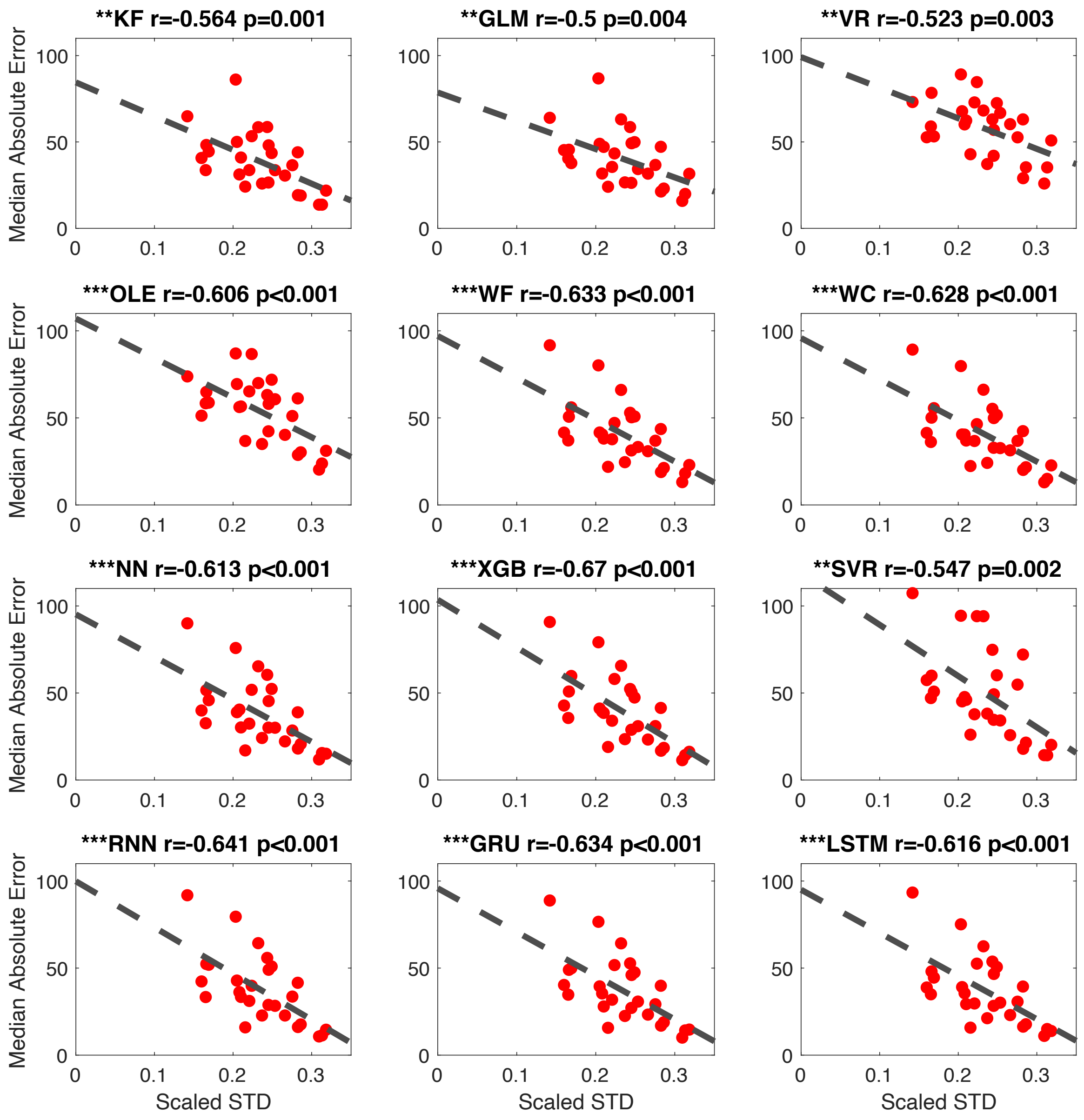

S13.11. *(Fig.11 LT version) Linear regression data is shown for each decoding method as a function of scaled STD (i.e. indicator of tuning strength). One cell was randomly selected from each dataset to avoid repeatedly sampling the same decoding score. All correlations are significant (p<0.01). The significance levels are shown with symbols on the top-left corner (***: p-value < 0.001; **: p-value < 0.01; *: p-value < 0.05). All correlations are significant (p<0.05). Kalman Filter (KF), Generalized Linear Model (GLM), Vector Reconstruction (VR), Wiener Filter (WF), Wiener Cascade (WC), Support Vector Regression (SVR), XGBoost (XGB), Feedforward Neural Network (FFNN), Recurrent Neural Network (RNN), Gated Recurrent Unit (GRU), and Long Short-Term Memory (LSTM).*

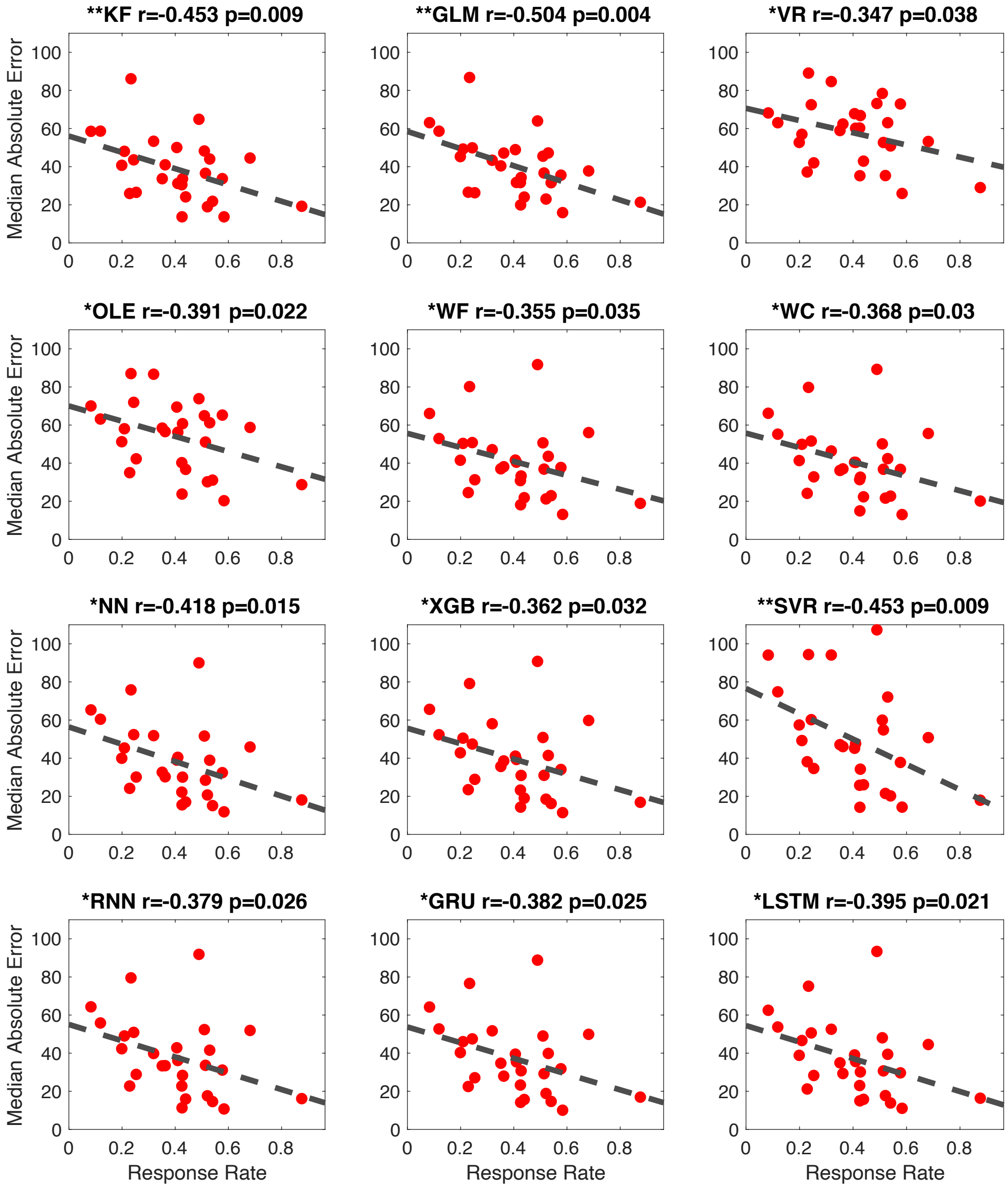

S13.12. *Scatterplots of median absolute error vs. response rate for all the 12 methods. The dashed line is the fitted linear regression. The correlation coefficient (r) and the corresponding p-value are shown on the top-right corner of each panel. The significance levels are shown with symbols on the top-left corner (***: p-value < 0.001; **: p-value < 0.01; *: p-value < 0.05). All correlations are significant (p<0.05). Kalman Filter (KF), Generalized Linear Model (GLM), Vector Reconstruction (VR), Wiener Filter (WF), Wiener Cascade (WC), Support Vector Regression (SVR), XGBoost (XGB), Feedforward Neural Network (FFNN), Recurrent Neural Network (RNN), Gated Recurrent Unit (GRU), and Long Short-Term Memory (LSTM).*

S14. The output for the case that using average / mean absolute error (AAE) as the measurement of the decoding performance. Tables and charts are made following the same scheme as **S9** to **S12**. Similarly, cross-validation is applied, so the results are separated into UT and LT two categories.

Mean Absolute Error Table															
Datasets		# Cells	Method												Datasets
Area	#		KF	GLM	VR	OLE	WF	WC	FFNN	XGB	SVR	RNN	GRU	LSTM	Mean
PoS	01	6	38.51	38.13	54.48	55.02	36.22	36.76	34.87	33.10	51.94	30.67	29.84	30.07	**39.13**
	03	6	38.38	37.15	58.10	57.57	45.15	42.72	38.21	44.07	44.20	40.78	38.18	39.41	**43.66**
	02	5	38.52	47.66	79.66	74.03	39.28	39.29	44.19	40.42	43.33	35.66	35.54	34.44	**46.00**
	05	5	48.19	48.76	79.52	79.34	49.84	50.18	46.98	49.09	55.58	49.32	44.25	44.05	**53.76**
	04	5	52.88	56.03	94.59	93.03	52.39	51.63	53.08	55.06	63.25	57.68	53.49	53.30	**61.37**
PaS	06	9	35.04	36.01	68.21	58.52	36.39	35.38	28.67	29.82	38.50	27.88	26.51	27.42	**37.36**
	05	5	46.91	54.01	68.44	67.96	48.70	48.54	45.19	49.37	55.71	44.11	45.63	44.79	**51.61**
	02	7	52.42	54.25	72.49	72.67	47.77	47.74	49.56	47.27	53.35	47.14	45.99	46.52	**53.10**
	03	6	64.82	65.42	76.48	77.58	55.39	55.55	59.43	56.86	62.50	57.33	54.36	54.44	**61.68**
	04	5	67.78	78.55	93.31	94.21	62.35	62.00	64.57	61.96	85.05	66.18	61.83	62.39	**71.68**
ATN	03	6	16.24	16.08	31.12	30.34	17.25	17.52	14.91	13.08	16.12	13.96	14.65	14.35	**17.97**
	02	6	24.19	27.20	41.11	38.67	26.03	24.75	20.79	20.64	22.23	17.48	21.10	20.31	**25.38**
	04	6	22.27	23.12	64.97	46.42	22.71	22.61	20.41	19.68	18.55	13.59	16.07	15.52	**25.49**
	01	6	16.40	19.04	87.77	61.28	17.53	18.97	19.17	19.06	17.84	15.81	16.05	15.55	**27.04**
	05	5	34.57	26.03	36.15	35.59	38.38	34.25	33.26	31.13	33.00	31.66	34.54	32.73	**33.44**
MEC	03	9	32.90	37.95	69.83	65.30	38.26	35.65	27.76	30.76	30.66	24.31	24.33	24.87	**36.88**
	05	5	37.85	37.85	76.61	76.85	42.70	41.81	40.87	40.84	54.66	39.48	37.21	39.41	**47.18**
	04	6	56.30	60.04	75.87	74.68	54.37	54.26	56.78	55.88	59.52	48.26	49.10	50.74	**57.98**
	01	6	66.79	67.01	80.29	79.69	55.04	54.86	58.44	55.91	70.88	61.12	53.08	53.86	**63.08**
	02	6	81.42	78.81	85.72	86.22	68.68	67.89	67.58	69.01	72.42	75.60	69.01	66.06	**74.03**
PC	05	6	46.56	46.18	52.87	51.10	41.16	42.11	42.67	41.49	51.23	45.26	42.43	42.73	**45.48**
	04	3	47.37	49.14	72.80	69.60	46.36	45.68	43.92	44.63	73.00	45.45	43.38	43.07	**52.03**
	07	3	44.47	46.31	65.06	62.52	51.74	49.93	58.11	51.61	64.50	54.35	49.20	51.02	**54.07**
	01	3	59.61	67.86	77.86	80.96	62.70	63.62	64.12	63.40	85.60	65.65	65.16	64.26	**68.40**
	06	3	63.86	76.91	93.72	84.52	68.69	69.50	71.73	70.39	73.77	70.93	69.89	70.79	**73.73**
	02	3	91.16	93.05	88.04	87.70	80.86	81.67	79.65	80.24	83.35	82.02	77.90	77.75	**83.61**
	03	3	87.65	84.43	89.15	86.91	81.22	80.89	82.30	82.06	94.01	83.25	81.46	82.72	**84.67**
**Methods Mean**			**48.63**	**50.85**	**71.64**	**68.46**	**47.67**	**47.25**	**46.93**	**46.55**	**54.62**	**46.11**	**44.45**	**44.54**	

S14.1. *The data table of the* ***AAE*** *values for all the datasets with every method, UT case. The methods marked as dark blue are statistical methods and the methods marked as orange are machine learning methods.*

Mean Absolute Error Table															
Datasets		# Cells	Method												Datasets Mean
Area	#		KF	GLM	VR	OLE	WF	WC	FFNN	XGB	SVR	RNN	GRU	LSTM	
PoS	03	6	37.15	37.45	52.09	49.92	34.66	34.34	35.41	32.76	45.12	31.95	31.74	30.77	37.78
	01	6	40.24	39.87	59.85	59.38	42.90	44.26	48.15	41.64	47.02	42.15	41.63	43.81	45.91
	02	5	47.59	50.65	79.70	76.00	50.85	49.81	49.97	48.77	53.64	46.17	48.32	47.42	54.08
	05	5	57.22	61.42	73.05	69.03	51.37	50.22	48.70	53.84	58.72	49.45	44.32	45.08	55.20
	04	5	59.01	62.62	79.29	79.02	63.64	65.44	63.15	59.21	69.95	64.39	59.95	59.48	65.43
PaS	06	9	39.39	41.65	69.55	56.91	42.26	43.03	34.06	33.87	36.97	34.27	32.21	32.48	41.39
	05	5	39.81	44.16	76.45	75.44	39.78	39.27	39.86	39.72	44.04	39.54	39.12	37.96	46.26
	03	6	57.05	58.44	77.80	76.60	53.33	53.47	51.95	53.53	54.37	54.88	53.61	52.17	58.10
	02	7	58.24	58.80	68.28	68.96	66.02	64.71	62.15	64.69	66.09	64.35	62.07	63.61	64.00
	04	5	71.18	73.51	74.38	73.89	76.08	76.04	75.32	76.21	91.70	74.05	74.60	75.16	76.01
ATN	03	6	18.34	22.39	31.20	28.35	18.04	17.53	15.73	14.54	18.43	13.51	13.89	14.49	18.87
	02	6	22.95	24.27	40.88	38.71	23.60	24.92	21.42	19.32	22.30	19.92	20.73	19.75	24.90
	01	6	19.37	35.82	52.53	40.92	26.03	21.56	19.53	18.70	22.24	16.06	17.54	18.02	25.69
	05	5	24.79	31.96	50.55	39.60	26.09	28.29	25.94	24.33	26.89	23.85	22.88	22.78	29.00
	04	6	37.93	51.37	68.83	55.90	35.95	37.64	25.53	29.76	36.39	26.98	26.89	23.76	38.08
MEC	03	9	31.72	32.98	62.36	55.76	30.60	31.30	26.69	27.40	33.50	24.08	24.32	24.05	33.73
	04	6	42.65	44.07	69.48	67.64	51.85	52.10	54.70	52.15	57.78	50.94	49.23	49.92	53.54
	05	5	47.56	56.18	71.34	71.10	50.58	49.47	47.31	49.21	60.61	46.77	47.52	47.39	53.75
	01	6	50.60	52.83	64.32	64.12	52.95	52.90	56.10	55.24	71.64	55.22	52.66	52.04	56.72
	02	6	70.43	70.58	72.30	72.26	67.96	69.12	70.13	66.18	76.50	68.91	66.94	65.79	69.76
PC	05	6	49.80	52.04	61.71	59.92	49.36	49.45	41.64	43.21	64.39	46.95	41.94	42.28	50.22
	04	3	55.53	58.41	73.59	71.09	57.74	56.49	54.39	55.79	77.49	55.24	53.44	53.94	60.26
	07	3	50.98	50.14	62.79	66.55	65.59	66.37	65.23	69.89	65.27	69.44	63.67	64.17	63.34
	06	3	59.68	60.62	85.44	73.56	63.24	62.20	63.85	62.65	70.31	64.64	62.10	61.46	65.81
	01	3	64.84	58.96	87.26	87.50	61.52	61.71	65.45	69.11	91.79	57.10	64.11	64.65	69.50
	02	3	89.43	86.60	88.77	88.45	83.84	83.82	80.81	83.27	91.16	83.27	80.71	80.15	85.02
	03	3	73.90	74.35	81.29	82.33	91.41	90.86	92.13	92.02	#####	91.49	91.03	94.78	88.11
Methods Mean			48.79	51.56	67.97	64.77	51.01	50.97	49.45	49.52	57.63	48.73	47.67	47.68	

S14.2. *The data table of the **AAE** values for all the datasets with every method, LT case. The methods marked as dark blue are statistical methods and the methods marked as orange are machine learning methods.*

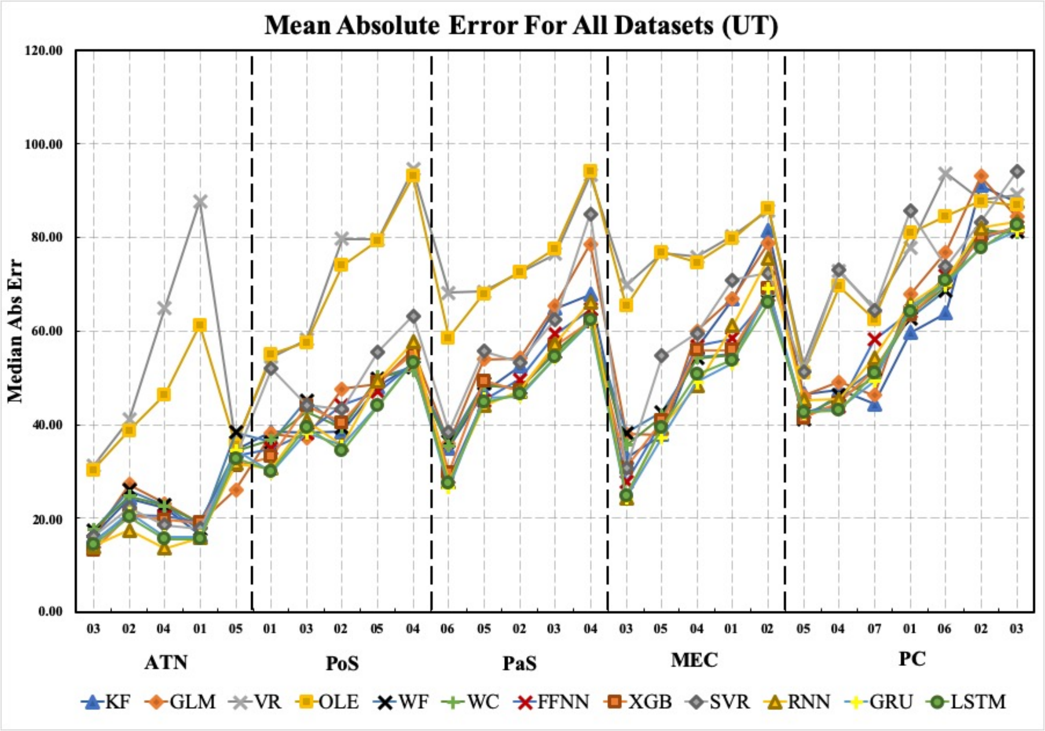

S14.3. *The average absolute error is shown for each dataset and each decoding method, UT case.*

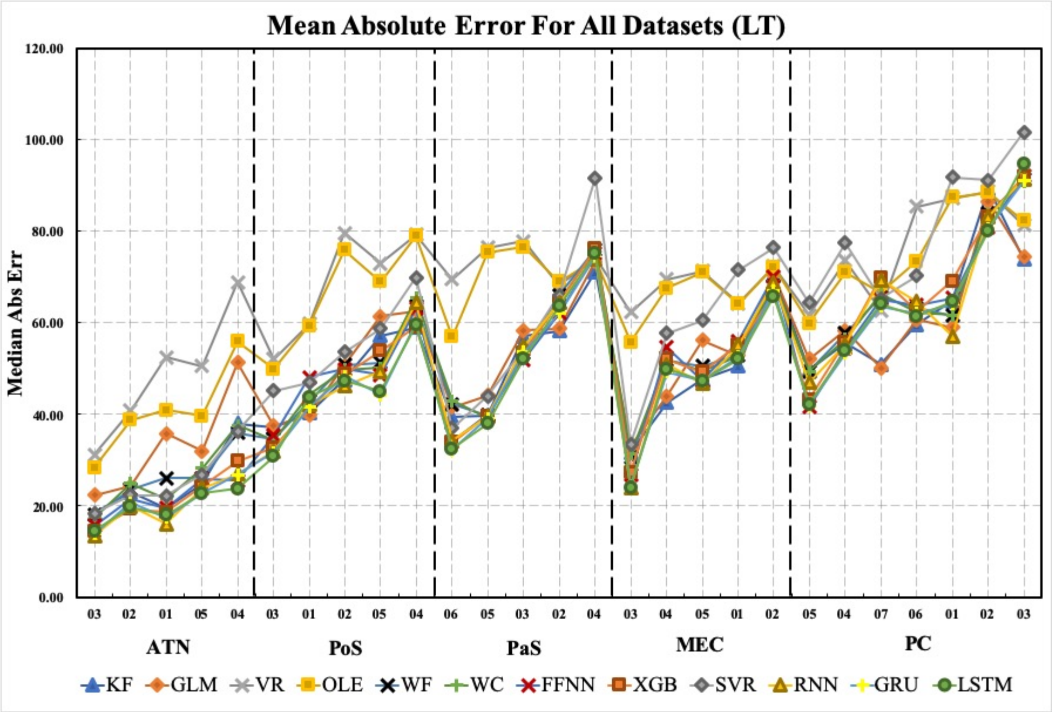

S14.4. *The average absolute error is shown for each dataset and each decoding method, LT case.*

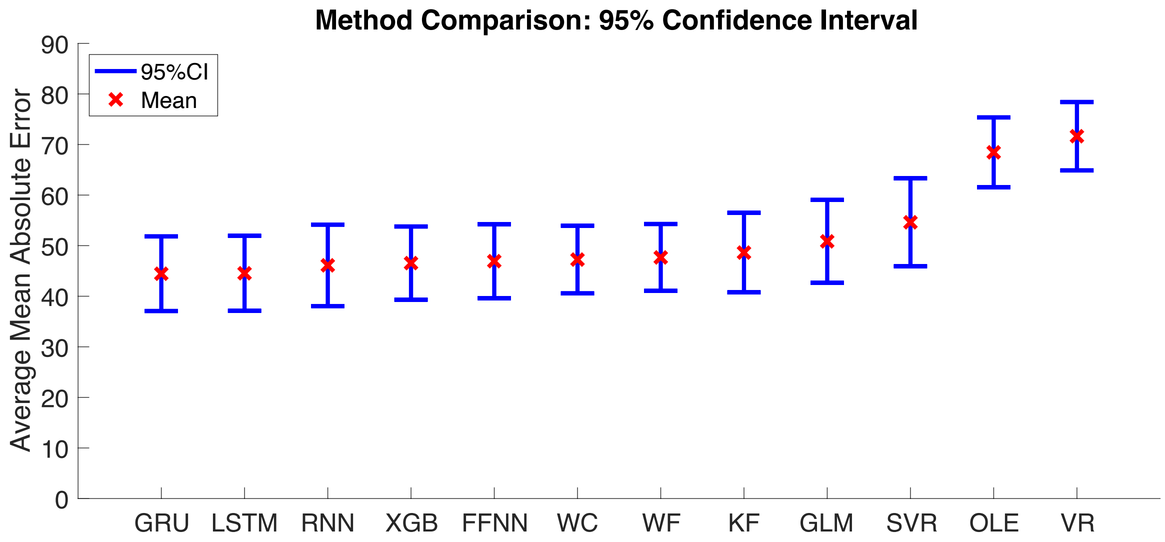

S14.5. *Mean±95% Confidence-Interval (CI) Average Absolute Error (AAE) for each decoding method, UT case. The ANOVA output: all 12 methods,* $F_{(11,\ 312)}$*=6.34, p<0.001; without VR and OLE,* $F_{(9,\ 260)}$*=0.69, p=0.72.*

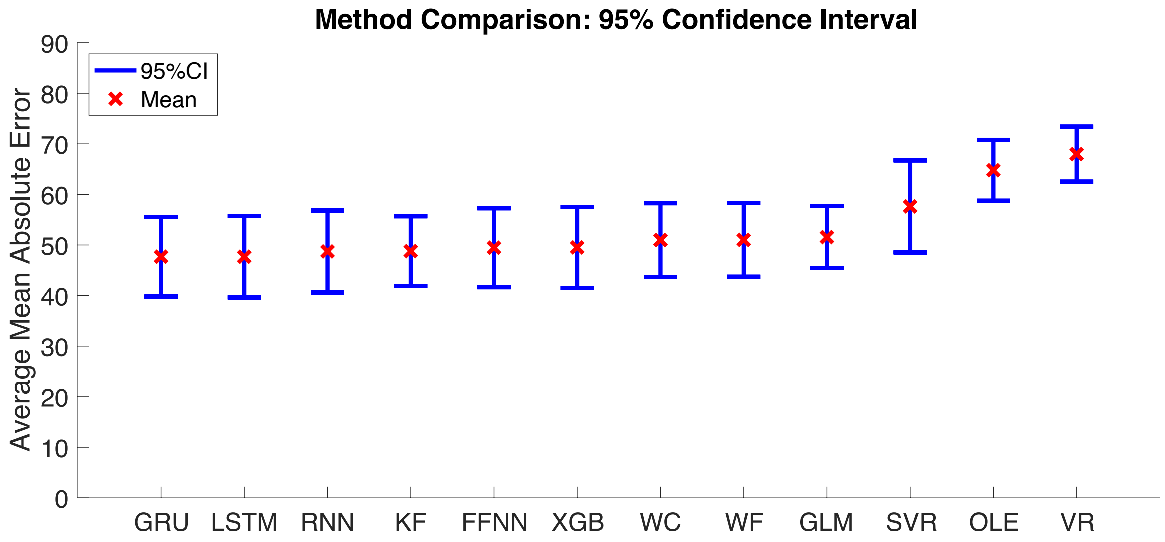

S14.6. *Mean±95% Confidence-Interval (CI) Average Absolute Error (AAE) for each decoding method, LT case. The ANOVA output: all 12 methods,* $F_{(11,\ 312)}$*=3.59, p<0.001; without VR and OLE,* $F_{(9,\ 260)}$*=0.60, p=0.79.*

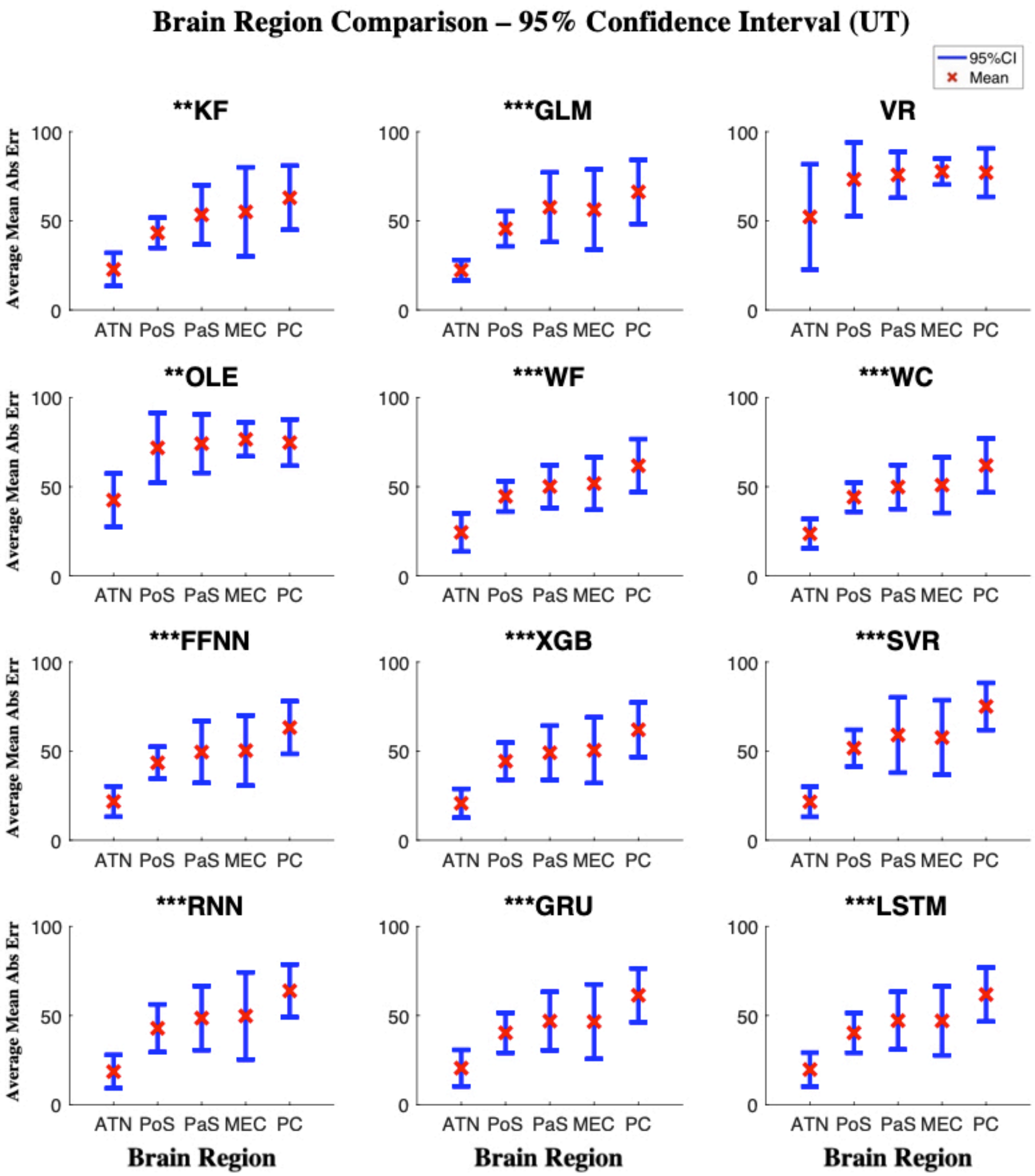

S14.7. *Average Median Absolute Error (AAE) values and error bars representing the 95% Confidence-intervals of the average AAE values for the 12 decoding methods, UT case. For 11 out of the 12 methods, accuracy significantly varied across brain region ($F_{(4, 22)}$>2.82, p<0.05). The exception, VR, has $F_{(4, 22)}$=2.49, p=0.07. For all the 11 significant methods, removing ATN resulted in a non-significant ANOVA ($F_{(3, 18)}$<3.16, p>0.05). The significance levels are shown with symbols on the top-left corner (***: p-value < 0.001; **: p-value < 0.01; *: p-value < 0.05). All correlations are significant (p<0.05). Kalman Filter (KF), Generalized Linear Model (GLM), Vector Reconstruction (VR), Wiener Filter (WF), Wiener Cascade (WC), Support Vector Regression (SVR), XGBoost (XGB), Feedforward Neural Network (FFNN), Recurrent Neural Network (RNN), Gated Recurrent Unit (GRU), and Long Short-Term Memory (LSTM).*

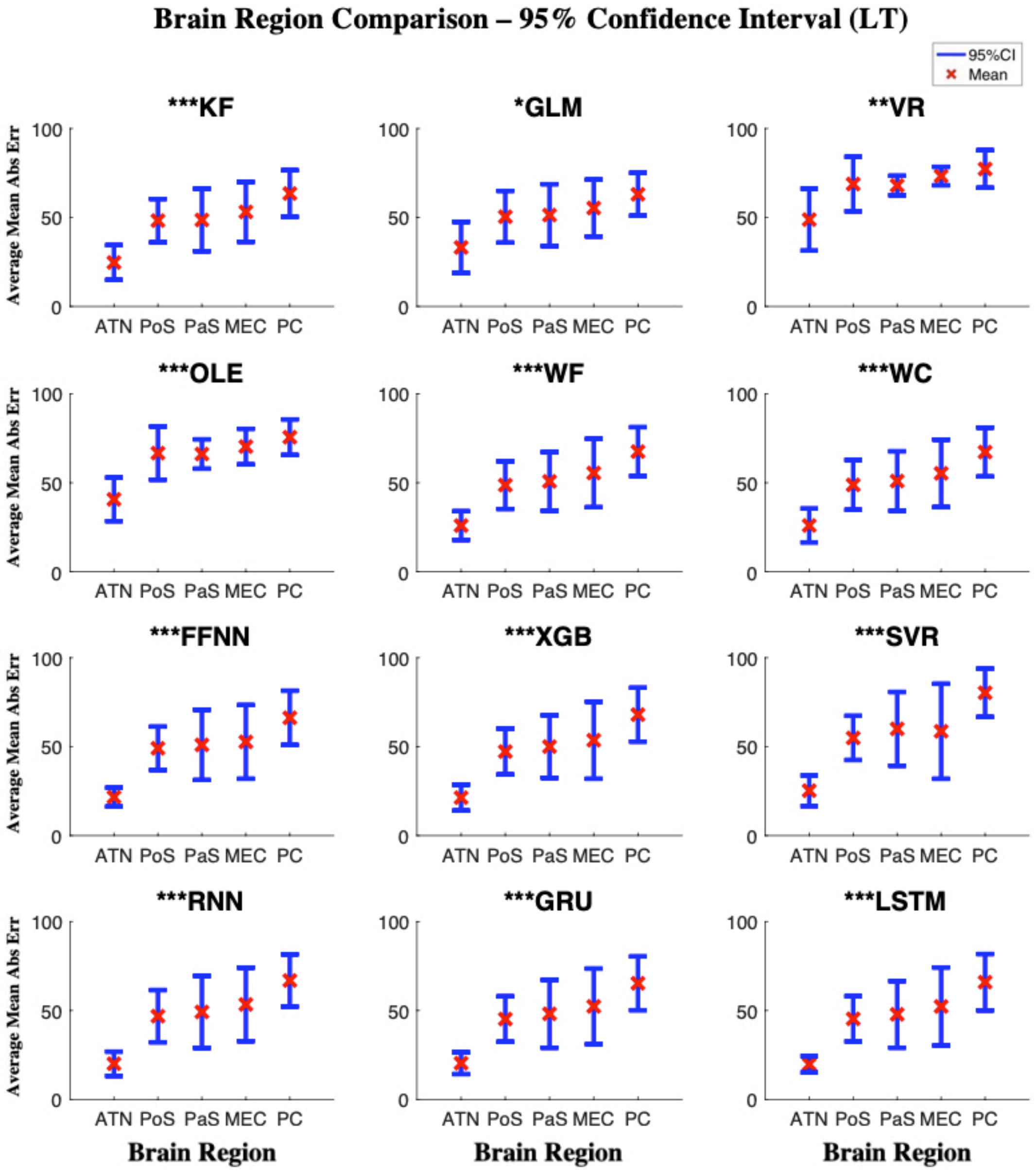

S14.8. *Average Median Absolute Error (AAE) values and error bars representing the 95% Confidence-intervals of the average AAE values for the 12 decoding methods, LT case. For all the 12 methods, accuracy significantly varied across brain region ($F_{(4, 22)}$>2.82, p<0.05). For all methods except SVR, removing ATN resulted in a non-significant ANOVA ($F_{(3, 18)}$<3.16, p>0.05). For SRV, it was necessary to also remove the brain region that was the second furthest from the mean, PC ($F_{(2,12)}$<3.89, p>0.05). The significance levels are shown with symbols on the top-left corner (***: p-value < 0.001; **: p-value < 0.01; *: p-value < 0.05). All correlations are significant (p<0.05). Kalman Filter (KF), Generalized Linear Model (GLM), Vector Reconstruction (VR), Wiener Filter (WF), Wiener Cascade (WC), Support Vector Regression (SVR), XGBoost (XGB), Feedforward Neural Network (FFNN), Recurrent Neural Network (RNN), Gated Recurrent Unit (GRU), and Long Short-Term Memory (LSTM).*

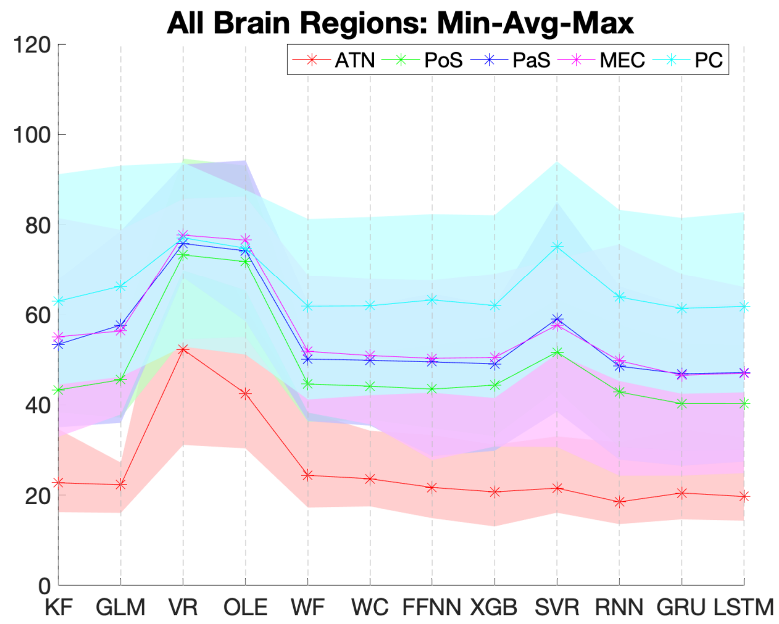

S14.9. *Decoding accuracy varies across brain regions, UT version.*

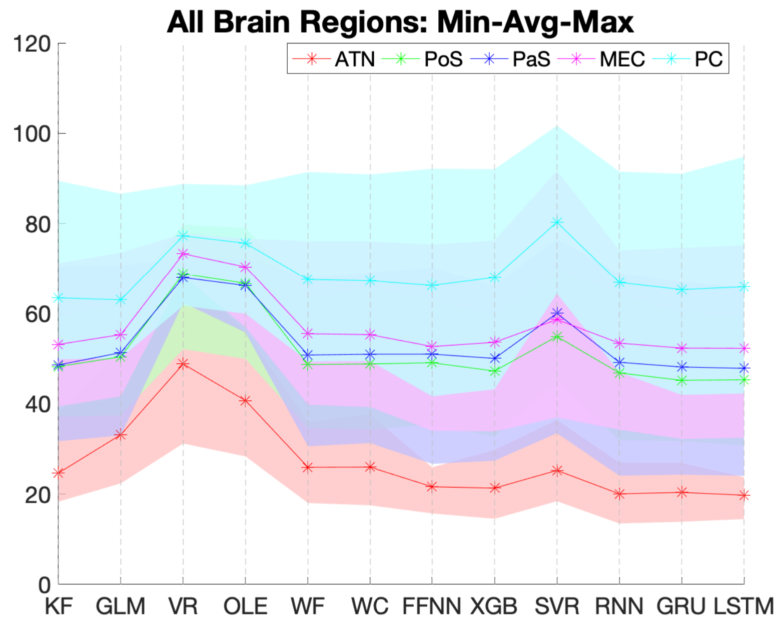

S14.10. *Decoding accuracy varies across brain regions, LT version.*

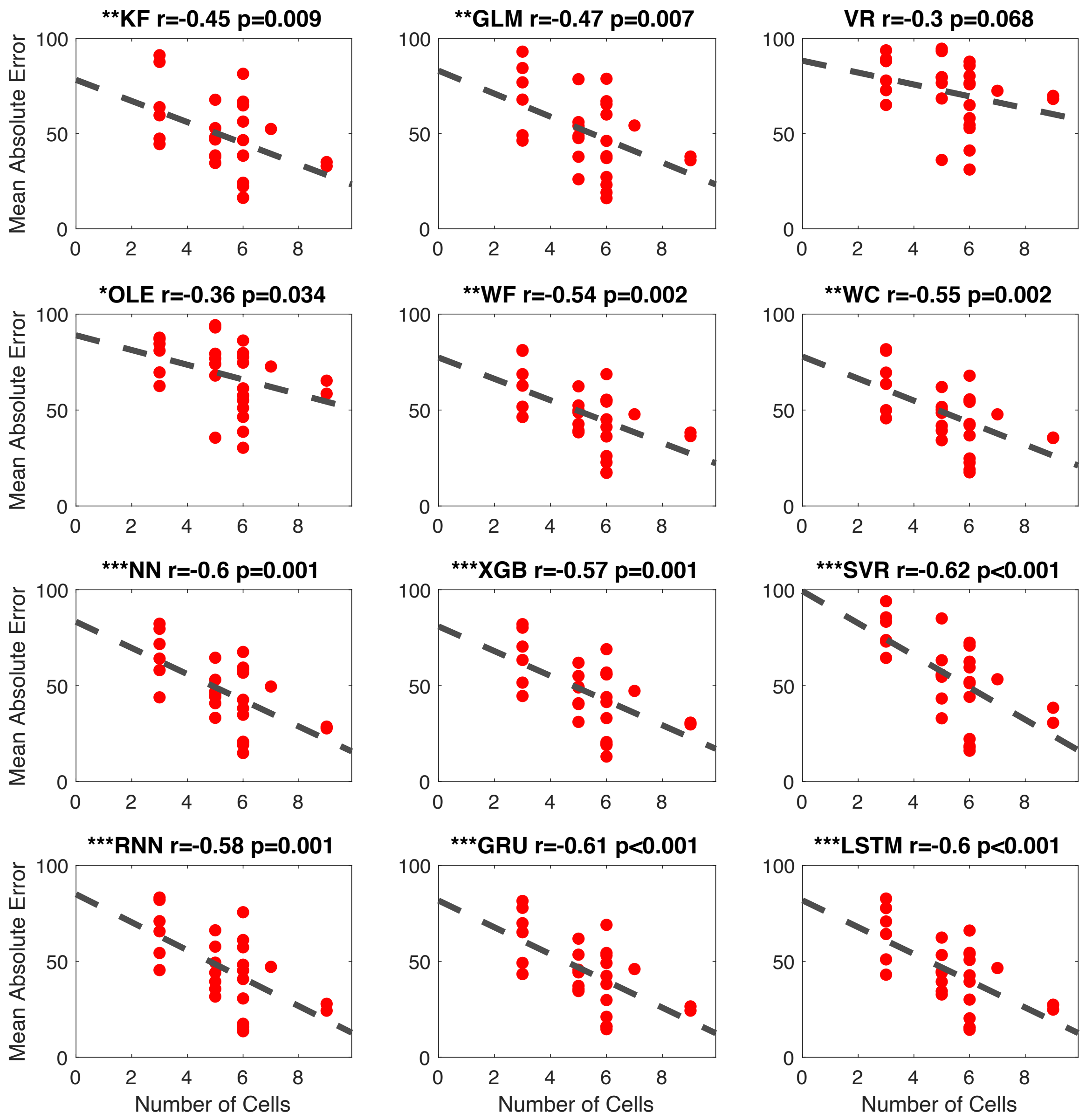

S14.11. *Scatterplots of average absolute error vs. number of cells for all 12 methods, UT version. The dashed line is the fitted linear regression. The correlation coefficient (r) and the corresponding p-value are shown on the top-right corner of each panel. The significance levels are shown with symbols on the top-left corner (***: p-value < 0.001; **: p-value < 0.01; *: p-value < 0.05). All correlations are significant (p<0.05). Kalman Filter (KF), Generalized Linear Model (GLM), Vector Reconstruction (VR), Wiener Filter (WF), Wiener Cascade (WC), Support Vector Regression (SVR), XGBoost (XGB), Feedforward Neural Network (FFNN), Recurrent Neural Network (RNN), Gated Recurrent Unit (GRU), and Long Short-Term Memory (LSTM).*

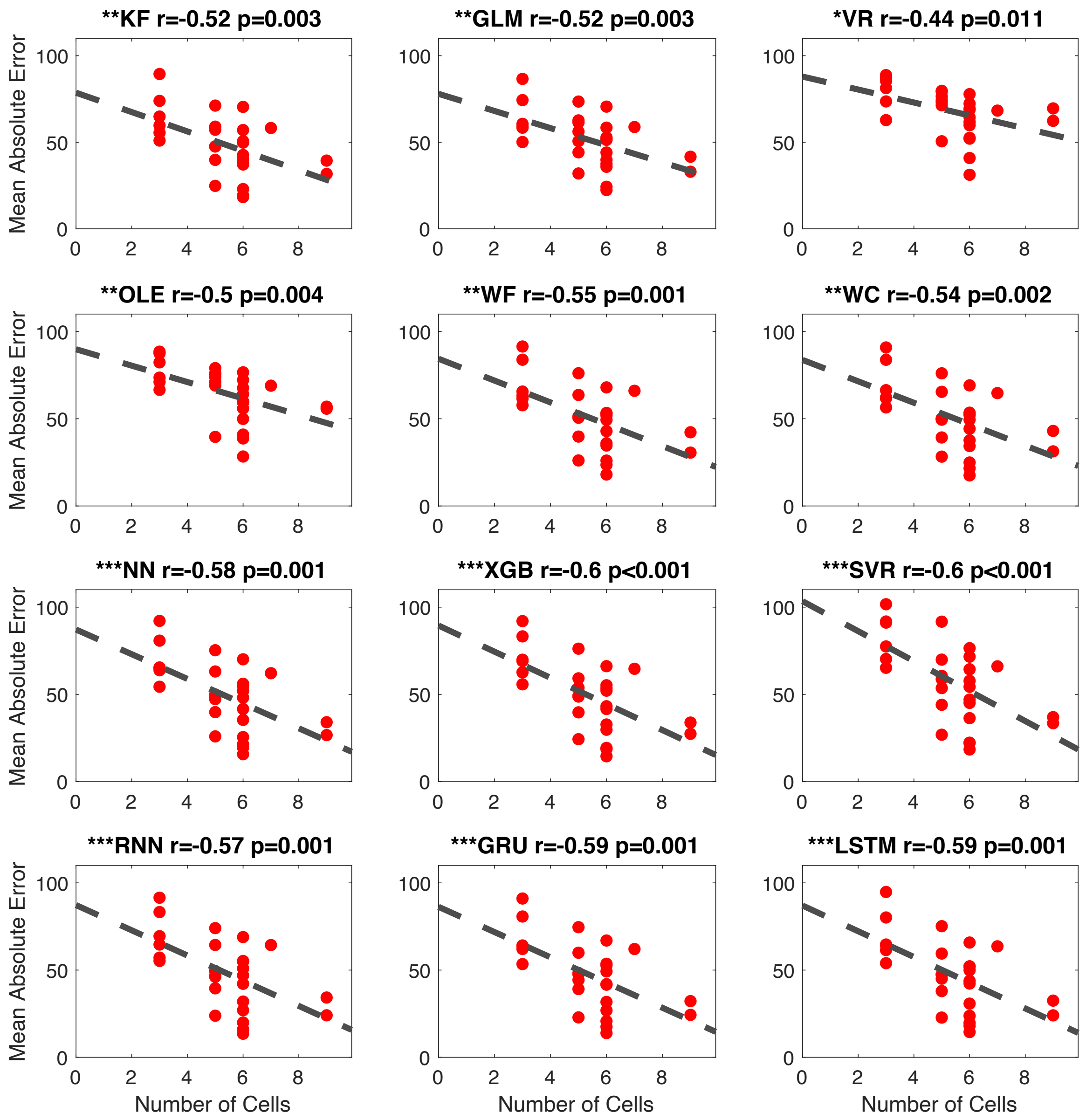

S14.12. *Scatterplots of average absolute error vs. number of cells for all 12 methods, LT version. The significance levels are shown with symbols on the top-left corner (***: p-value < 0.001; **: p-value < 0.01; *: p-value < 0.05). All correlations are significant (p<0.05). Kalman Filter (KF), Generalized Linear Model (GLM), Vector Reconstruction (VR), Wiener Filter (WF), Wiener Cascade (WC), Support Vector Regression (SVR), XGBoost (XGB), Feedforward Neural Network (FFNN), Recurrent Neural Network (RNN), Gated Recurrent Unit (GRU), and Long Short-Term Memory (LSTM).*

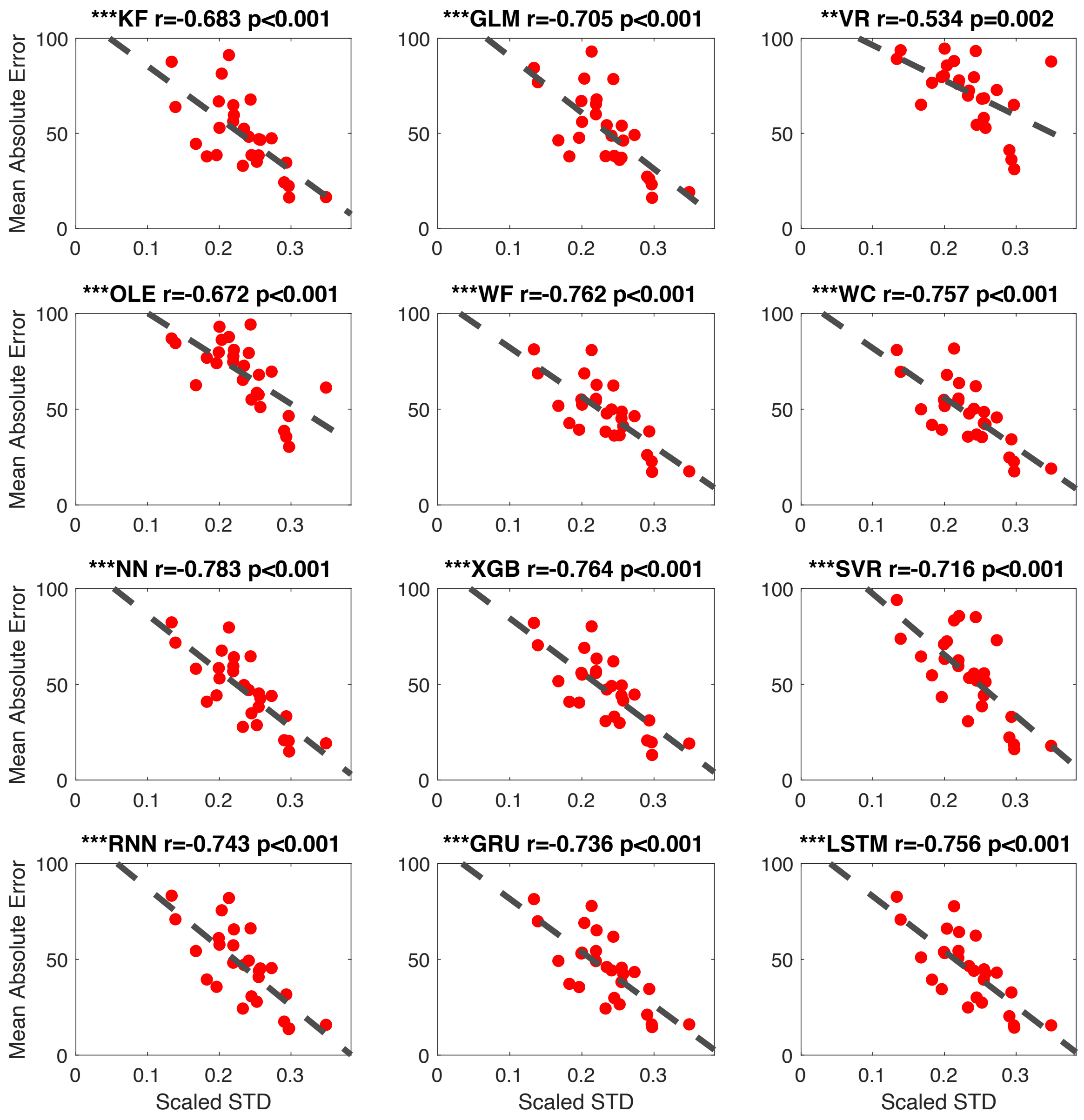

S14.13. *Linear regression data is shown for each decoding method as a function of scaled STD, UT version. One cell was randomly selected from each dataset to avoid repeatedly sampling the same decoding score. All correlations are significant (p<0.01). The significance levels are shown with symbols on the top-left corner (***: p-value < 0.001; **: p-value < 0.01; *: p-value < 0.05). All correlations are significant (p<0.05). Kalman Filter (KF), Generalized Linear Model (GLM), Vector Reconstruction (VR), Wiener Filter (WF), Wiener Cascade (WC), Support Vector Regression (SVR), XGBoost (XGB), Feedforward Neural Network (FFNN), Recurrent Neural Network (RNN), Gated Recurrent Unit (GRU), and Long Short-Term Memory (LSTM).*

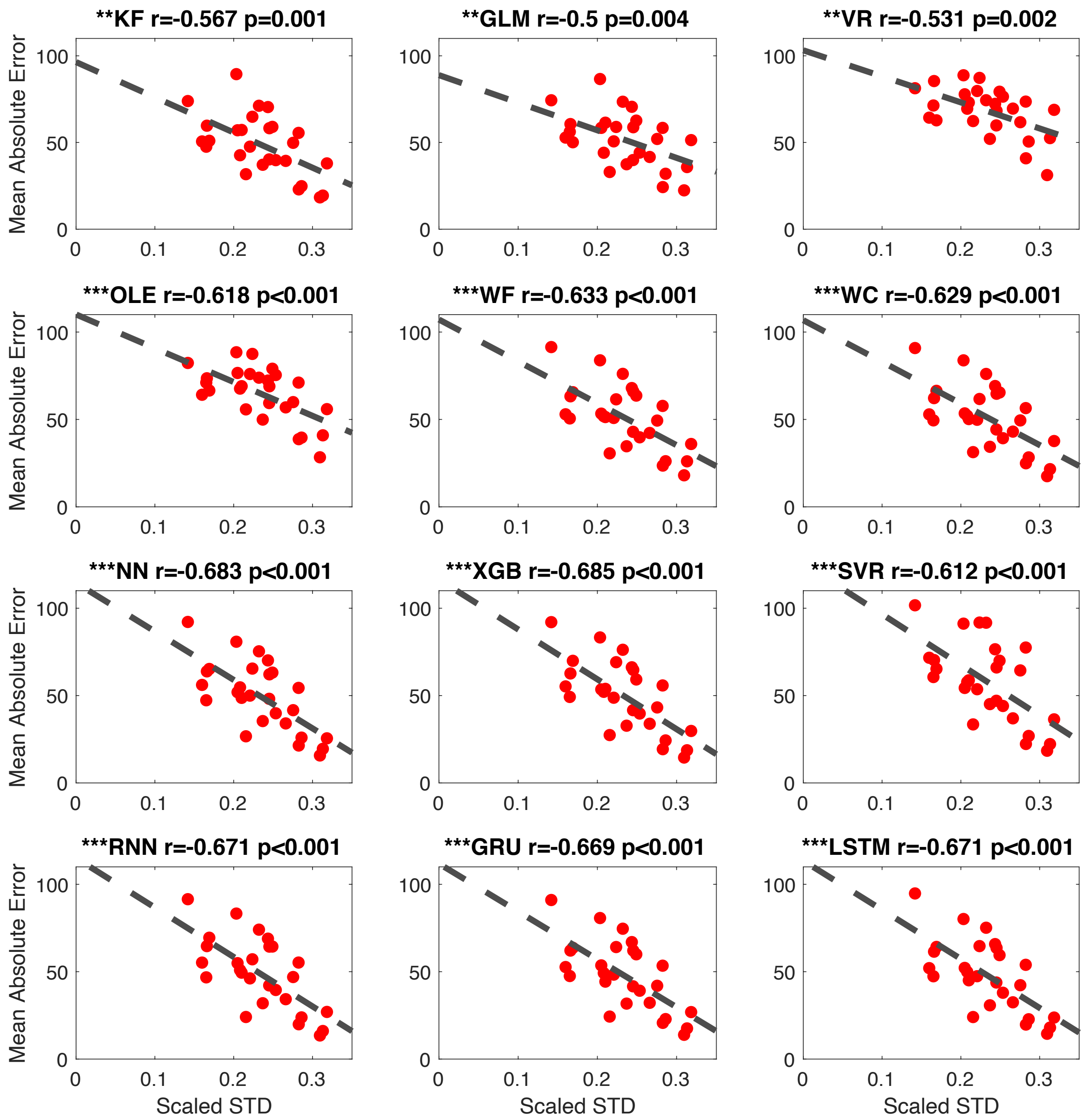

S14.14. *Linear regression data is shown for each decoding method as a function of scaled STD, LT version. All correlations are significant (p<0.01). The significance levels are shown with symbols on the top-left corner (***: p-value < 0.001; **: p-value < 0.01; *: p-value < 0.05). All correlations are significant (p<0.05). Kalman Filter (KF), Generalized Linear Model (GLM), Vector Reconstruction (VR), Wiener Filter (WF), Wiener Cascade (WC), Support Vector Regression (SVR), XGBoost (XGB), Feedforward Neural Network (FFNN), Recurrent Neural Network (RNN), Gated Recurrent Unit (GRU), and Long Short-Term Memory (LSTM).*

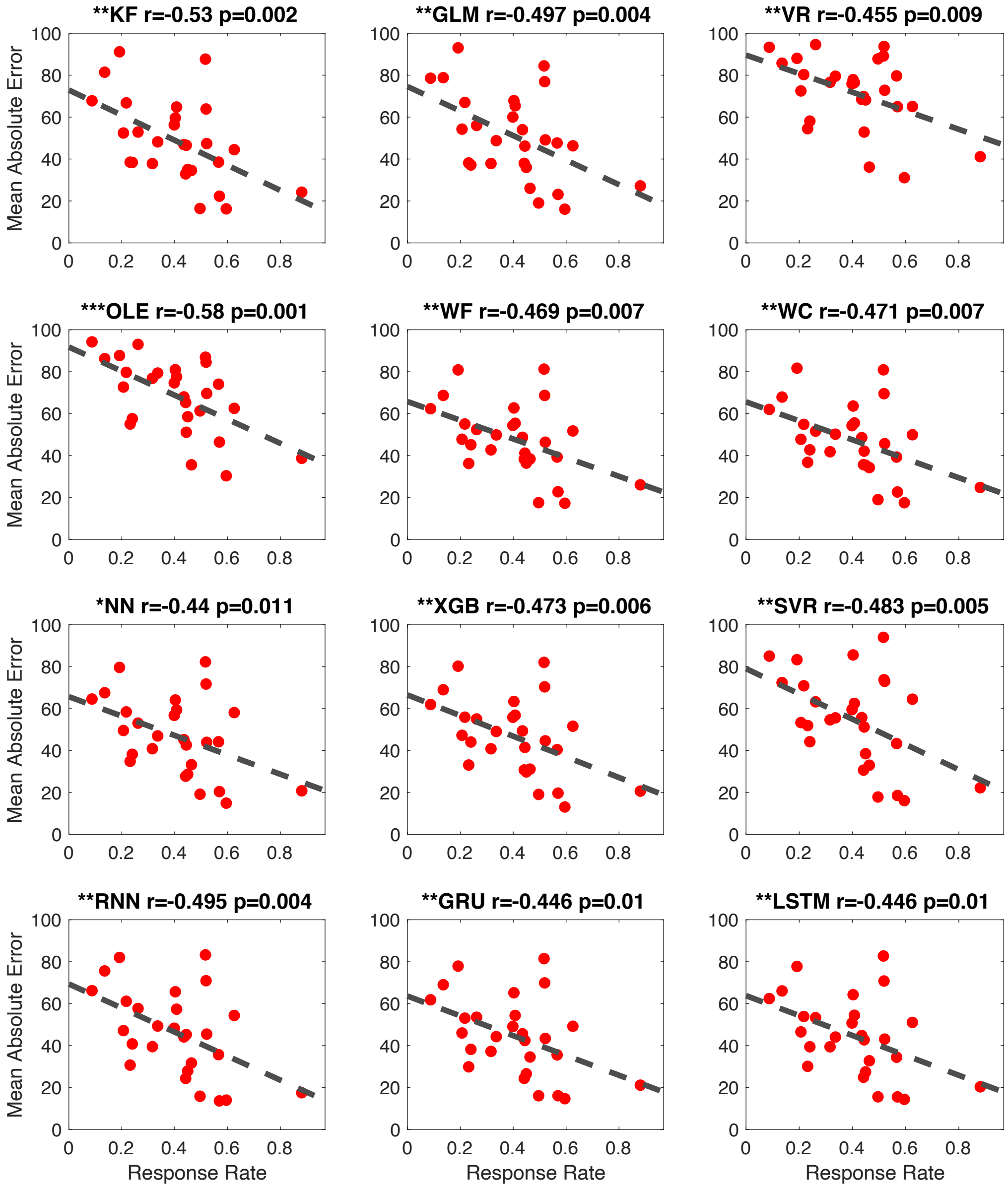

S14.15. *Scatterplots of average absolute error vs. response rate for all the 12 methods, UT version. The dashed line is the fitted linear regression. The correlation coefficient (r) and the corresponding p-value are shown on the top-right corner of each panel. The significance levels are shown with symbols on the top-left corner (***: p-value < 0.001; **: p-value < 0.01; *: p-value < 0.05). All correlations are significant (p<0.05). Kalman Filter (KF), Generalized Linear Model (GLM), Vector Reconstruction (VR), Wiener Filter (WF), Wiener Cascade (WC), Support Vector Regression (SVR), XGBoost (XGB), Feedforward Neural Network (FFNN), Recurrent Neural Network (RNN), Gated Recurrent Unit (GRU), and Long Short-Term Memory (LSTM).*

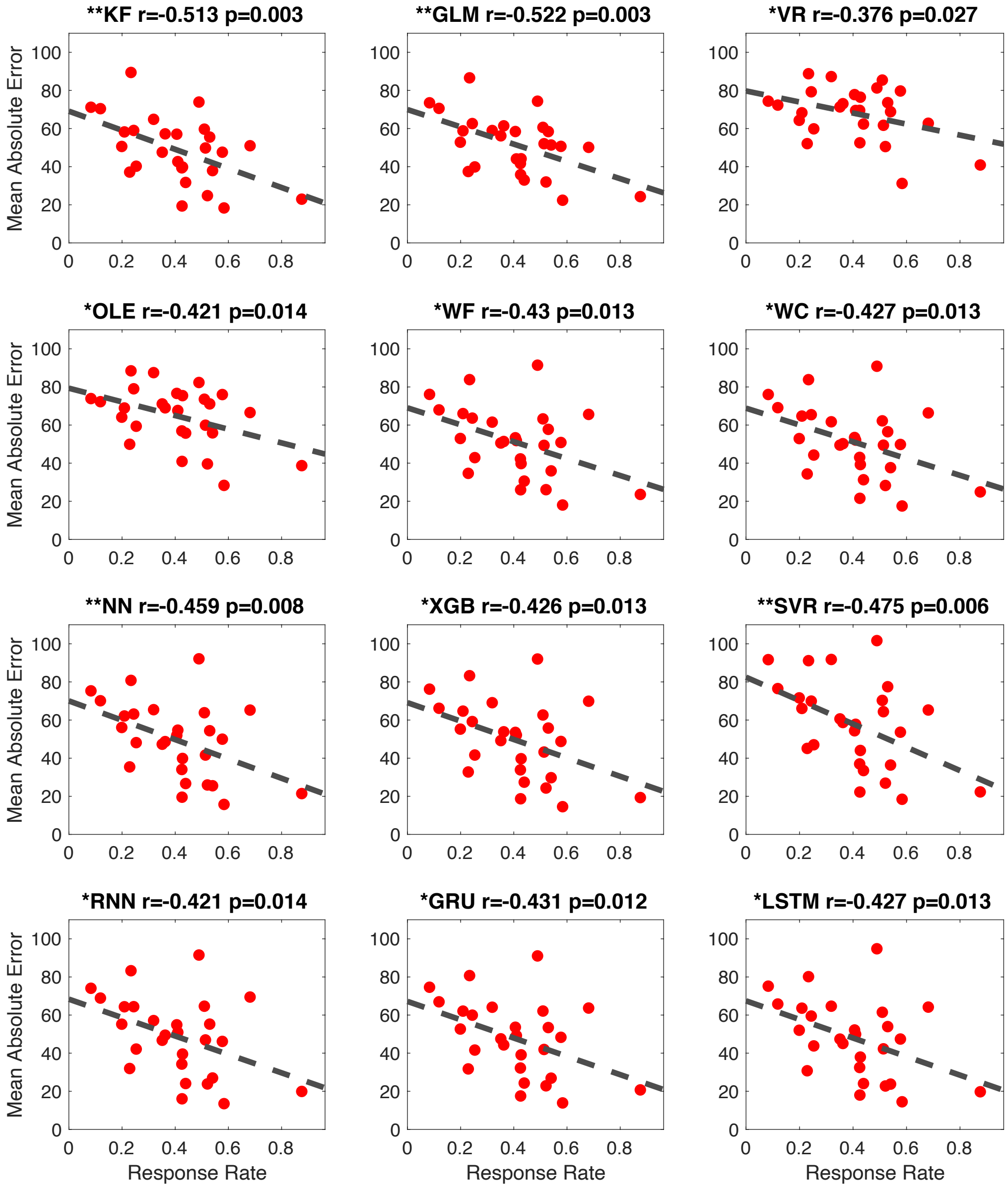

S14.16. *Scatterplots of average absolute error vs. response rate for all the 12 methods, LT version. The significance levels are shown with symbols on the top-left corner (***: p-value < 0.001; **: p-value < 0.01; *: p-value < 0.05). All correlations are significant (p<0.05). Kalman Filter (KF), Generalized Linear Model (GLM), Vector Reconstruction (VR), Wiener Filter (WF), Wiener Cascade (WC), Support Vector Regression (SVR), XGBoost (XGB), Feedforward Neural Network (FFNN), Recurrent Neural Network (RNN), Gated Recurrent Unit (GRU), and Long Short-Term Memory (LSTM).*

S15. To control for potential influence of the number of cells on the finding that ATN performed significantly better than other brain regions, we repeated the analyses that led to this conclusion but randomly sub-sampled so that all data sets now consisted of 3 cells. Since sub-sampling will result in more data sets, due to the large computational demands for machine learning methods, this repeated analysis was only performed on the model-based methods. As shown in Fig. 7 machine-learning methods should produce similar results. Similarly, cross-validation was applied so the results are separated into UT and LT two categories.

Median Absolute Error Table								
Datasets		Method						Datasets
Area	#	KF	GLM	VR	OLE	WF	WC	Mean
PoS	1	34.02	34.26	57.41	54.52	33.91	34.54	**41.44**
	2	36.71	45.11	55.58	56.31	39.01	37.24	**44.99**
	3	33.00	34.39	70.35	66.80	34.49	35.38	**45.73**
	4	33.23	39.15	71.07	66.13	34.66	34.63	**46.48**
	5	33.60	23.94	52.61	53.13	35.50	32.54	**38.55**
	6	40.99	31.07	60.63	59.96	73.97	63.63	**55.04**
	7	34.36	37.82	105.78	103.79	36.66	34.67	**58.84**
	8	50.65	49.50	104.04	104.93	46.71	48.71	**67.42**
	9	32.77	38.32	78.42	80.11	35.82	38.29	**50.62**
	10	42.50	40.43	75.05	75.07	40.10	40.94	**52.35**
PaS	1	63.37	61.91	90.00	90.00	58.43	59.21	**70.49**
	2	62.75	62.90	74.05	75.07	55.12	52.30	**63.70**
	3	47.78	49.75	71.63	71.63	38.36	39.28	**53.07**
	4	74.40	74.86	77.90	81.12	64.22	63.74	**72.71**
	5	52.34	54.28	77.11	78.77	50.03	51.30	**60.64**
	6	55.22	80.82	100.31	100.62	55.52	58.96	**75.24**
	7	55.07	57.64	99.46	100.30	58.01	58.28	**71.46**
	8	45.19	45.40	56.37	54.46	50.24	48.14	**49.97**
	9	37.15	37.74	58.00	56.27	37.39	37.82	**44.06**
	10	29.67	25.94	63.43	60.95	30.07	26.79	**39.47**
	11	53.48	55.06	70.02	68.20	49.15	50.61	**57.75**
	12	40.31	39.16	70.02	69.44	41.56	41.49	**50.33**
ATN	1	20.63	18.96	114.78	79.24	24.67	21.69	**46.66**
	2	15.09	19.07	93.14	90.00	19.73	22.28	**43.22**
	3	24.53	26.78	36.81	32.84	28.07	26.76	**29.30**
	4	27.71	29.72	35.29	35.07	28.61	27.50	**30.65**
	5	15.01	15.84	26.87	24.71	15.14	14.51	**18.68**
	6	23.92	21.33	36.70	28.30	22.13	21.28	**25.61**
	7	23.04	29.73	56.38	43.41	23.61	22.72	**33.15**
	8	18.43	19.65	45.00	30.94	19.65	17.92	**25.26**
	9	26.22	27.29	48.57	38.95	34.58	24.24	**33.31**
	10	26.28	21.53	30.94	31.99	29.12	26.76	**27.77**
MEC	1	61.71	64.09	73.17	73.26	51.98	52.06	**62.71**
	2	57.11	57.99	85.24	85.02	54.90	53.61	**65.64**
	3	81.53	76.51	93.58	93.58	75.61	74.17	**82.49**
	4	71.73	69.39	98.13	100.01	70.30	68.85	**79.74**
	5	50.94	50.21	98.44	98.58	55.80	50.49	**67.41**
	6	34.06	35.89	100.16	99.73	41.98	34.87	**57.78**
	7	29.99	34.91	79.60	74.83	35.58	33.43	**48.06**
	8	43.40	38.64	99.43	104.10	43.51	42.06	**61.86**
	9	56.04	56.30	90.00	85.92	54.51	59.55	**67.05**
	10	24.66	25.31	129.29	129.29	34.53	36.62	**63.28**
	11	32.95	33.37	72.90	73.34	35.56	35.88	**47.33**
PC	1	42.14	55.95	67.94	68.96	52.32	51.27	**56.43**
	2	91.88	96.15	88.73	86.75	77.09	77.41	**86.33**
	3	88.63	86.73	89.48	84.76	77.01	76.80	**83.90**
	4	40.28	42.84	61.61	57.13	35.84	34.92	**45.44**
	5	36.87	36.80	43.66	54.10	33.40	35.74	**40.09**
	6	56.94	61.71	106.00	102.59	42.20	44.73	**69.03**
	7	47.14	73.30	92.19	82.00	56.16	57.15	**67.99**
	8	31.80	32.00	53.54	50.97	33.84	32.61	**39.13**
Method Mean		42.92	44.66	74.25	71.92	43.26	42.48	**53.25**

Median Absolute Error Table								
Datasets		Method						Datasets
Area	#	KF	GLM	VR	OLE	WF	WC	Mean
PoS	1	47.45	52.09	57.53	57.97	43.95	45.10	**50.68**
	2	37.27	35.56	60.95	60.94	42.52	38.57	**45.97**
	3	35.36	38.14	72.47	68.58	40.11	40.16	**49.14**
	4	35.37	39.13	68.20	66.25	38.41	39.14	**47.75**
	5	28.42	31.46	42.05	41.18	33.04	32.82	**34.83**
	6	30.56	31.57	56.31	52.13	32.36	33.38	**39.39**
	7	44.45	46.56	74.05	75.04	52.74	51.11	**57.33**
	8	47.38	52.42	71.56	71.57	57.45	59.21	**59.93**
	9	46.99	45.20	63.43	60.98	48.90	44.89	**51.73**
	10	37.58	39.10	67.49	64.99	49.98	46.51	**50.94**
PaS	1	74.40	78.41	71.77	72.90	69.87	67.88	**72.54**
	2	53.88	50.55	60.60	59.74	60.21	57.20	**57.03**
	3	60.97	63.82	61.70	61.39	72.17	69.01	**64.84**
	4	60.66	65.00	73.63	74.70	55.79	55.81	**64.27**
	5	61.00	62.53	70.02	70.35	53.75	52.64	**61.71**
	6	69.33	77.66	72.10	74.05	77.45	77.24	**74.64**
	7	66.51	66.50	72.90	73.30	82.21	80.15	**73.59**
	8	51.06	58.61	77.47	75.07	46.75	48.11	**59.51**
	9	33.19	37.29	73.82	67.49	33.17	32.88	**46.31**
	10	29.46	30.79	59.67	55.31	30.83	30.55	**39.43**
	11	52.02	48.59	69.49	67.28	52.95	53.64	**57.33**
	12	51.40	50.77	77.00	68.20	52.87	52.84	**58.85**
ATN	1	24.05	26.48	45.92	29.68	23.44	15.43	**27.50**
	2	25.48	21.50	34.69	37.14	41.09	30.17	**31.68**
	3	24.10	26.65	47.90	46.36	24.63	25.09	**32.45**
	4	25.02	23.74	32.05	34.13	29.05	30.76	**29.13**
	5	16.25	14.89	29.65	26.77	16.16	16.78	**20.08**
	6	20.12	20.23	38.92	28.42	18.66	17.78	**24.02**
	7	35.79	46.51	65.50	61.20	39.75	40.80	**48.26**
	8	23.82	26.03	42.80	28.50	21.35	19.97	**27.08**
	9	23.46	23.96	66.97	43.14	26.42	27.57	**35.25**
	10	26.13	22.78	36.75	41.82	25.85	27.03	**30.06**
MEC	1	44.92	52.67	53.97	53.13	52.29	54.18	**51.86**
	2	50.41	52.20	67.38	67.38	45.35	43.68	**54.40**
	3	77.50	72.99	70.35	68.39	68.88	69.59	**71.28**
	4	79.97	87.04	63.43	63.44	72.18	70.70	**72.79**
	5	42.32	50.19	90.00	89.96	46.14	44.81	**60.57**
	6	38.91	36.66	83.21	83.60	37.49	37.58	**52.91**
	7	34.36	31.64	67.12	63.46	32.59	33.25	**43.74**
	8	48.68	44.63	99.41	98.75	49.39	46.99	**64.64**
	9	38.10	37.71	68.34	61.17	46.29	47.59	**49.86**
	10	46.00	42.66	85.24	83.14	47.42	46.58	**58.51**
	11	40.58	39.61	59.77	60.93	42.90	42.12	**47.65**
PC	1	53.33	44.22	84.65	86.84	47.08	46.40	**60.42**
	2	86.12	89.09	89.10	87.08	80.17	79.77	**85.22**
	3	64.86	64.76	73.14	73.73	91.71	89.22	**76.24**
	4	44.01	45.56	63.07	61.26	43.59	42.41	**49.98**
	5	45.91	47.88	56.22	53.27	48.32	48.28	**49.98**
	6	35.37	34.40	56.49	53.00	51.27	50.64	**46.86**
	7	48.24	46.76	78.43	65.48	50.67	50.15	**56.62**
	8	44.51	39.26	53.22	58.69	56.04	55.59	**51.22**
Method Mean		44.37	45.38	64.27	61.75	47.13	46.27	**51.53**

S15.1 *The **MAE** values for all the datasets organized by brain region (rows) and methods (columns). Separate tables were generated for UT (left) and LT (right) cross validation approaches.*

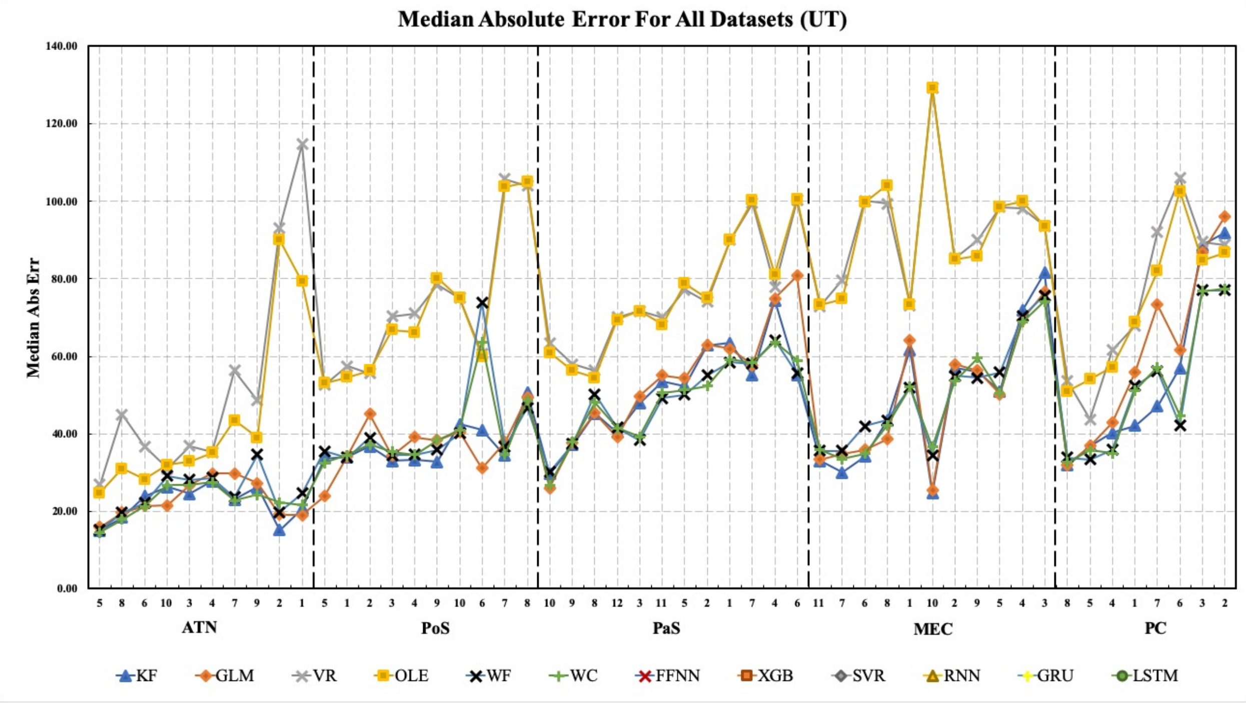

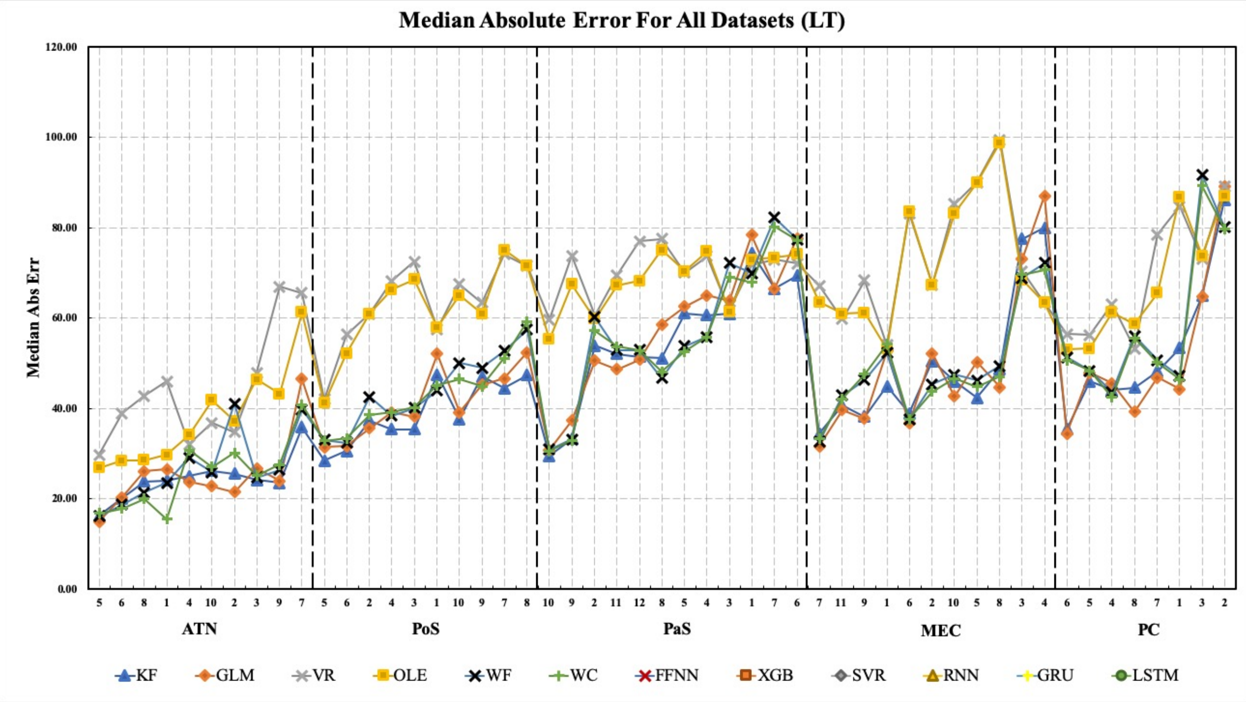

S15.2 *The **MAE** values are shown for each data set and each decoding method (as in **Fig. 7**). Separate figures were generated for UT (top) and LT (bottom) cross validation approaches.*

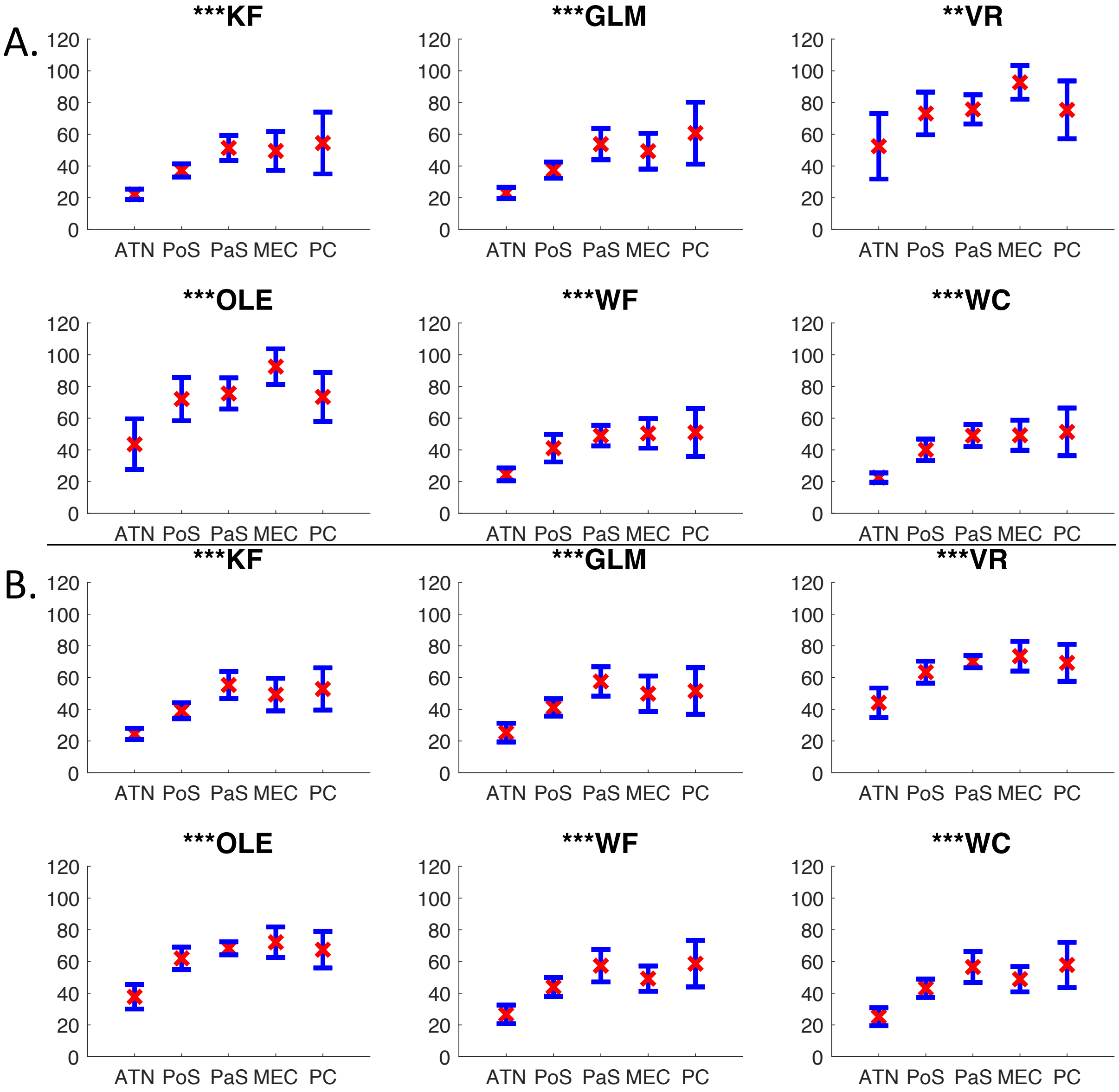

S15.3 *The average MAE collapsed across all datasets within each area for each decoding method (as in* ***Fig. 9*** *right). The error bars represent the 95% Confidence-intervals of the average MAE values. For all the methods, accuracy significantly varied across brain region ($F_{(4, 46)}$>2.57, p<0.05). Removing ATN results in non-significant ANOVA for KF, WF and WC ($F_{(3, 37)}$<2.86, p>0.05), but not for the other three methods. For GLM, removing both ATN and PoS gives a non-significant ANOVA ($F_{(2, 28)}$<3.34, p>0.05). For VR and OLE, removing both ATN and MEC gives a non-significant ANOVA ($F_{(2, 27)}$<3.34, p>0.05). Separate figures were generated for UT (**A**) and LT (**B**) cross validation approaches. The significance levels are shown with symbols on the top-left corner (***: p-value < 0.001; **: p-value < 0.01; *: p-value < 0.05). All correlations are significant (p<0.05). Kalman Filter (KF), Generalized Linear Model (GLM), Vector Reconstruction (VR), Wiener Filter (WF), Wiener Cascade (WC), Support Vector Regression (SVR), XGBoost (XGB), Feedforward Neural Network (FFNN), Recurrent Neural Network (RNN), Gated Recurrent Unit (GRU), and Long Short-Term Memory (LSTM).*

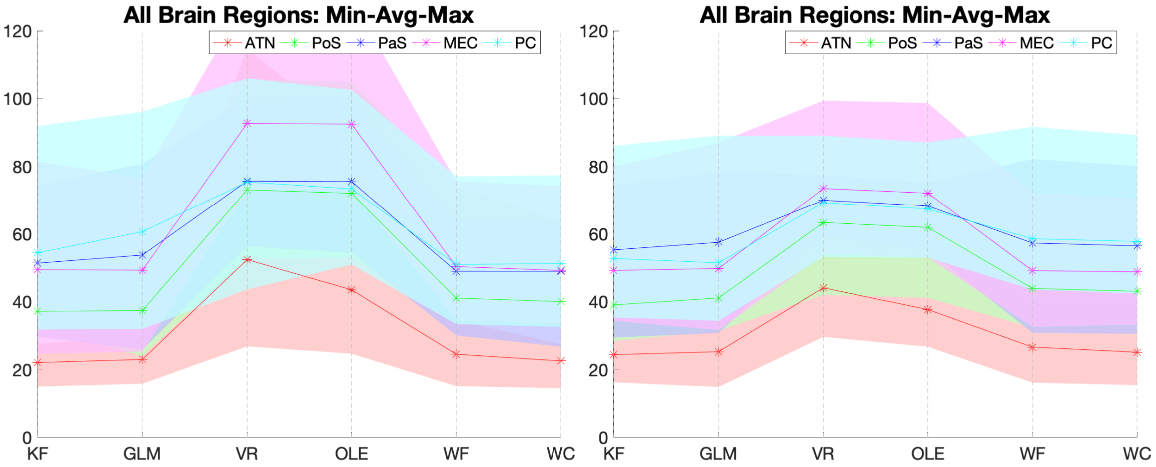

S15.4 *Decoding accuracy varies across brain regions (as in* ***Fig. 9*** *left). The shading represents the range of the MAE values. Separate figures were generated for UT (left) and LT (right) cross validation approaches.*

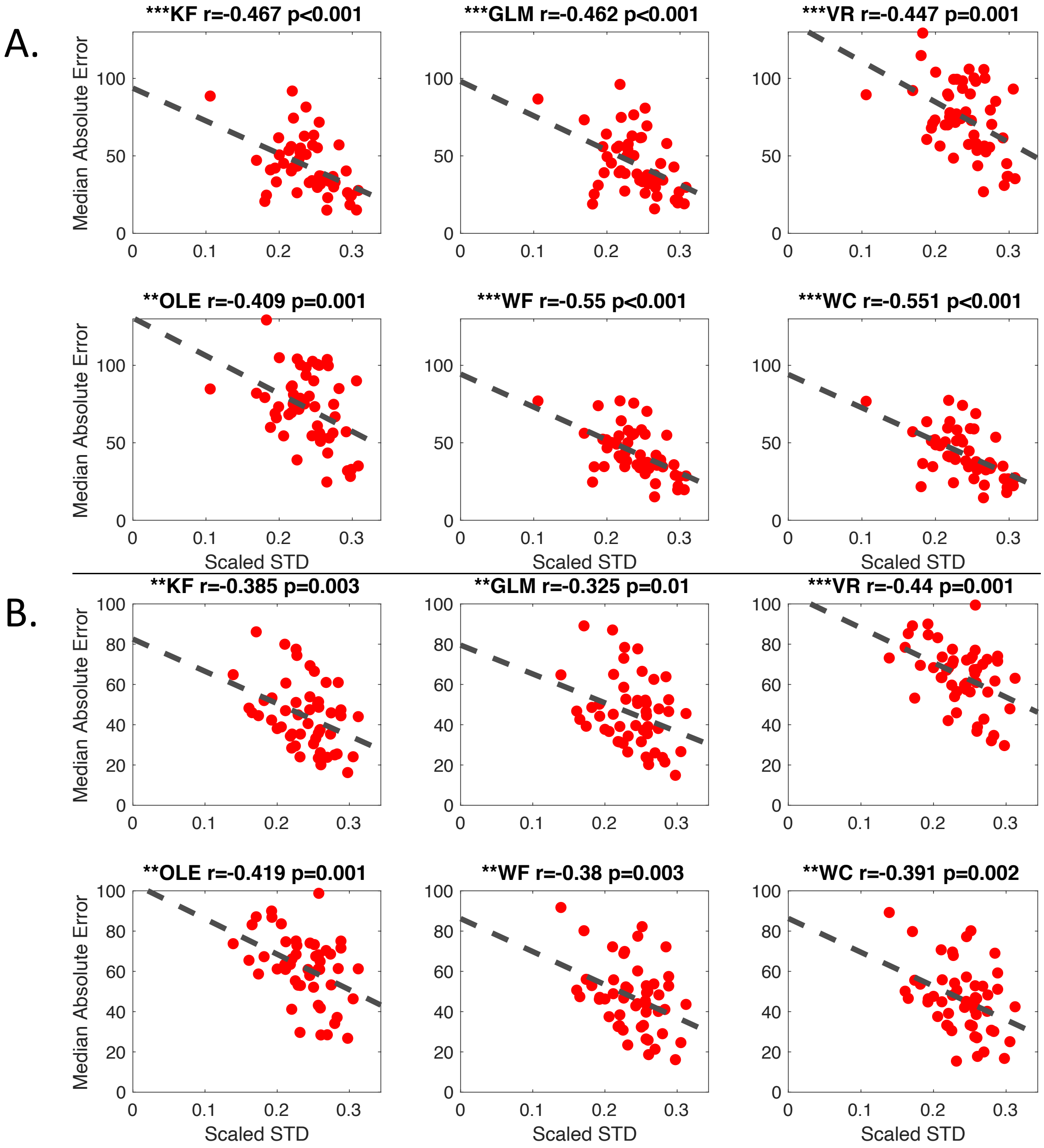

S15.5 *Tuning's effect on decoding accuracy (as in* ***Fig. 11*** *bottom). Linear regression data is shown for each decoding method as a function of scaled STD. All correlations are significant (p<0.01). Separate figures were generated for UT (****A****) and LT (****B****) cross validation approaches. The significance levels are shown with symbols on the top-left corner (***: p-value < 0.001; **: p-value < 0.01; *: p-value < 0.05). All correlations are significant (p<0.05). Kalman Filter (KF), Generalized Linear Model (GLM), Vector Reconstruction (VR), Wiener Filter (WF), Wiener Cascade (WC), Support Vector Regression (SVR), XGBoost (XGB), Feedforward Neural Network (FFNN), Recurrent Neural Network (RNN), Gated Recurrent Unit (GRU), and Long Short-Term Memory (LSTM).*

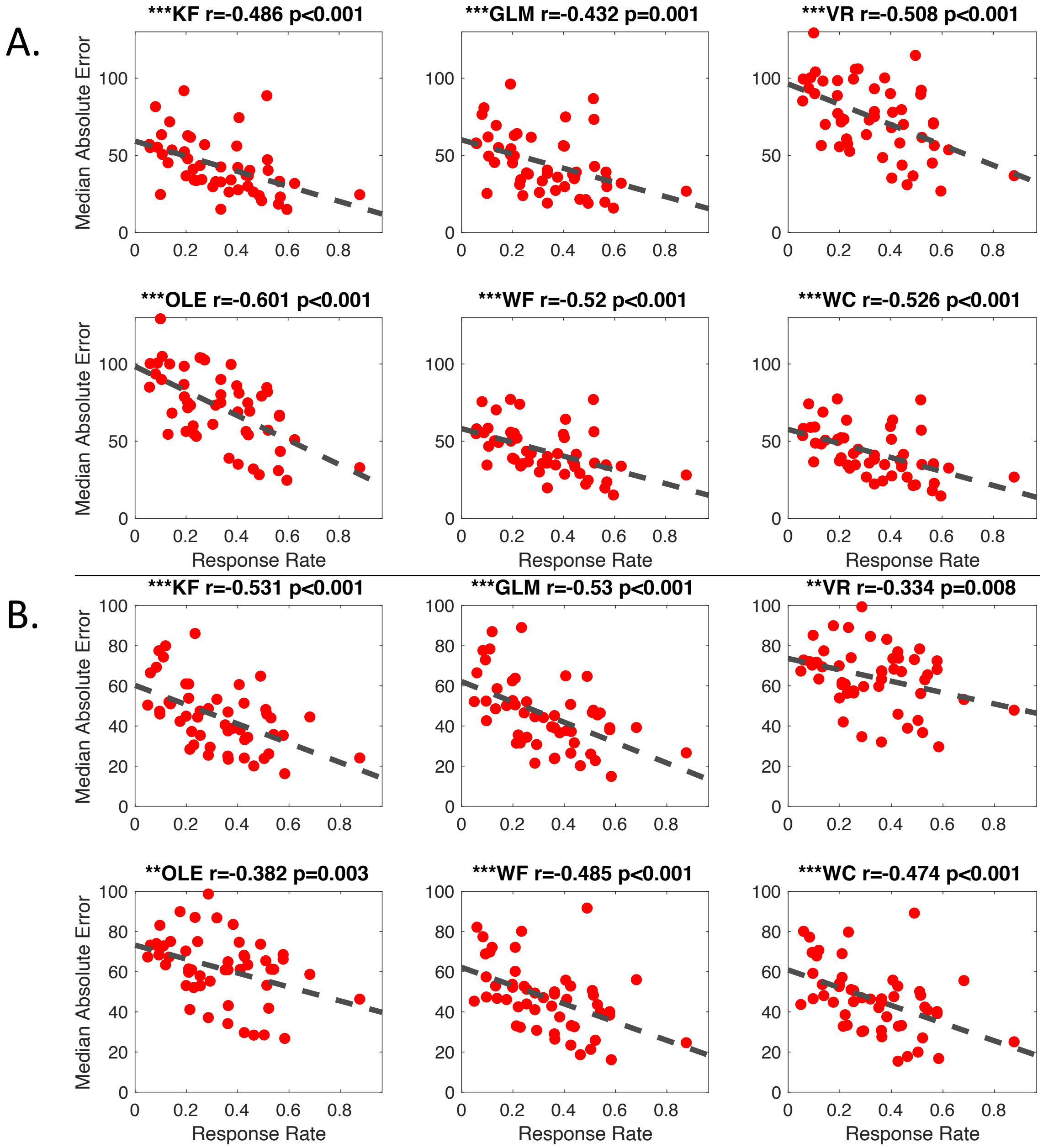

S15.6 *Firing rate's effect on decoding accuracy (w.r.t. Fig. 12 bottom right). Each figure is the scatterplot of average absolute error vs. response rate including the regression line. All correlations are significant (p<0.01). Separate figures were generated for UT (**A**) and LT (**B**) cross validation approaches. The significance levels are shown with symbols on the top-left corner (***: p-value < 0.001; **: p-value < 0.01; *: p-value < 0.05). All correlations are significant (p<0.05). Kalman Filter (KF), Generalized Linear Model (GLM), Vector Reconstruction (VR), Wiener Filter (WF), Wiener Cascade (WC), Support Vector Regression (SVR), XGBoost (XGB), Feedforward Neural Network (FFNN), Recurrent Neural Network (RNN), Gated Recurrent Unit (GRU), and Long Short-Term Memory (LSTM).*
